# Single‐base methylome profiling of the giant kelp *Saccharina japonica* reveals significant differences in DNA methylation to microalgae and plants

**DOI:** 10.1111/nph.16125

**Published:** 2019-09-27

**Authors:** Xiao Fan, Wentao Han, Linhong Teng, Peng Jiang, Xiaowen Zhang, Dong Xu, Chang Li, Matteo Pellegrini, Chunhui Wu, Yitao Wang, Michelle Joyce Slade Kaczurowski, Xin Lin, Leila Tirichine, Thomas Mock, Naihao Ye

**Affiliations:** ^1^ Yellow Sea Fisheries Research Institute Chinese Academy of Fishery Sciences Qingdao China; ^2^ Function Laboratory for Marine Fisheries Science and Food Production Processes Qingdao China; ^3^ Key Laboratory of Exploration and Utilization of Aquatic Genetic Resources Ministry of Education Shanghai Ocean University Shanghai 201306 China; ^4^ College of Life Science Dezhou University Dezhou 253023 China; ^5^ Shandong Key Laboratory of Biophysics Dezhou University Dezhou 253023 China; ^6^ Institute of Oceanology Chinese Academy of Sciences Qingdao 266071 China; ^7^ University of Chinese Academy of Sciences Shenzhen China; ^8^ Department of Molecular, Cell and Developmental Biology Institute for Genomics and Proteomics University of California Los Angeles CA 90095 USA; ^9^ Biological Sciences Flinders University GPO Box 2100 Adelaide SA 5001 Australia; ^10^ State Key Laboratory of Marine Environmental Science College of Ocean & Earth Sciences Xiamen University Xiamen China; ^11^ CNRS UMR 6286 Faculté des Sciences et des Techniques Université de Nantes 2 rue de la Houssinière 44322 Nantes France; ^12^ School of Environmental Sciences University of East Anglia Norwich Research Park Norwich NR4 7TJ UK

**Keywords:** brown algae, BS‐PCR, DNA methylation, DNMT2, gene expression, life‐cycle stages, MeDIP‐seq, WGBS‐seq

## Abstract

- Brown algae have convergently evolved plant‐like body plans and reproductive cycles, which in plants are controlled by differential DNA methylation. This contribution provides the first single‐base methylome profiles of haploid gametophytes and diploid sporophytes of a multicellular alga.
- Although only *c*. 1.4% of cytosines in *Saccharina japonica* were methylated mainly at CHH sites and characterized by 5‐methylcytosine (5mC), there were significant differences between life‐cycle stages. DNA methyltransferase 2 (DNMT2), known to efficiently catalyze tRNA methylation, is assumed to methylate the genome of *S. japonica* in the structural context of tRNAs as the genome does not encode any other DNA methyltransferases. Circular and long noncoding RNA genes were the most strongly methylated regulatory elements in *S. japonica*.
- Differential expression of genes was negatively correlated with DNA methylation with the highest methylation levels measured in both haploid gametophytes. Hypomethylated and highly expressed genes in diploid sporophytes included genes involved in morphogenesis and halogen metabolism.
- The data herein provide evidence that cytosine methylation, although occurring at a low level, is significantly contributing to the formation of different life‐cycle stages, tissue differentiation and metabolism in brown algae.

## Introduction

Cytosine DNA methylation is a common epigenetic mark essential for genomic imprinting, X‐chromosome inactivation and silencing of transposable elements, as well as regulation of gene expression in many species (Meissner *et al*., 2008; Feng *et al*., 2010; Law & Jacobsen, 2010; Stelzer *et al*., 2015). Genome‐wide methylation studies with many different plant and animal species but also unicellular eukaryotes (e.g. fungi, algae) over the last few years have revealed significant inter‐ and intraspecific variations in cytosine DNA methylation (Cokus *et al*., 2008; Lee *et al*., 2010; Molaro *et al*., 2011; Greaves *et al*., 2012; Ziller *et al*., 2013). For example, vertebrate genomes are characterized by significant levels of cytosine methylation, whereas genomes of invertebrates, plants and fungi are characterized by sparse DNA methylation (Sturgill *et al*., 2007; Feng *et al*., 2010). Generally, the degree of DNA methylation is positively correlated with the complexity of organisms; thus, prokaryotes and unicellular eukaryotes have much lower levels of DNA methylation compared to organisms with complex life cycles, developmental stages and cell‐type differentiation (Molaro *et al*., 2011; Lopez *et al*., 2015). Furthermore, there are significant differences in establishing, maintaining and modifying DNA methylation in plants vs animals. For instance, in plants, DNA methylation has been observed for GC, CHG and CHH contexts with H being any nucleotide but G. DNA methylation in plants occurs predominantly on repetitive DNA elements (e.g. transposons) regardless of their life cycle and developmental stages (Lopez *et al*., 2015; Takuno *et al*., 2016). Similar DNA methylation patterns have been observed for unicellular photosynthetic eukaryotes (e.g. microalgae) (Veluchamy *et al*., 2013). By contrast, DNA methylation in mammals mostly occurs in the GC context throughout the genome except in clusters near promoters (CpG islands) (Molaro *et al*., 2011; Smith *et al*., 2012; Hon *et al*., 2013; Kundaje *et al*., 2015). Generally, DNA methylation in plants and animals is dynamic with genome‐wide reduction during both male and female gametogenesis as well as development (Molaro *et al*., 2011; Shao *et al*., 2014; Lopez *et al*., 2015). However, in plants, many DNA methylation patterns seem to be inherited over many generations whereas transgenerational DNA methylation in animals is much more variable (Hon *et al*., 2013; Lister *et al*., 2013; Shao *et al*., 2014).

Brown algae, although only distantly related to plants and animals, have convergently evolved plant‐like body plans and reproductive cycles including male and female gametogenesis (Charrier *et al*., 2012). Furthermore, many brown algae have an alternative life cycle with two life stages, termed gametophytes (1N) and sporophytes (2N) (Charrier *et al*., 2012; Cock *et al*., 2014). Gametophytes from some kelp species, such as *Saccharina japonica,* can even develop into larger multicellular organisms but rarely reach tissue differentiation (Ye *et al*., 2015). Some other brown algae, such as *Ectocarpus siliculosus,* have isomorphic life‐cycle stages that are filamentous and phenotypically indistinguishable from one another (Luthringer *et al*., 2014).

Brown algae comprise a group of *c*. 2000 species, possessing a large variety of phenotypes including the largest multicellular photosynthetic organisms in the ocean with distinct and specialized tissue differentiation, such as holdfast, blade and stripe. Despite their convergently evolved plant‐like body plans, their ecological (e.g. main primary producers of temperate and polar rocky shores) and commercial (e.g. alginate, fucoidan) significance, genomes of only three brown algal species are available to date: *E. siliculosus* (Cock *et al*., 2010), *S. japonica* (Ye *et al*., 2015) and *Cladosiphon okamuranus* (Nishitsuji *et al*., 2016). A comparative study based on the genomes of *E. siliculosus* and *S. japonica* revealed that they share 4309 gene families, which comprise 17 379 genes in *S. japonica* and 14 136 genes in *E. siliculosus*, covering 93% and 86% of the gene content of each genome, respectively. About 40% of the assembled *S. japonica* genome comprises repetitive elements, which is nearly twice as much as for the *E. siliculosus* genome (*c*. 23%) (Ye *et al*., 2015).

DNA methylation in brown algal genomes was considered to be negligible based on preliminary high performance liquid chromatography (HPLC) analyses of deoxycytosine methylation (5mdC) of hydrolyzed DNA from *E. siliculosus* (Cock *et al*., 2010). These data indicated that the percentage of 5mdC in *E. siliculosus* is < 0.035%. However, the present study using single‐base DNA methylome profiling of a fully developed *S. japonica* sporophyte (SP), female (FG) and male (MG) gametophyte revealed that *c*. 1.4% of all cytosines in *S. japonica* were methylated in GC, CHG and CHH contexts potentially mediated by a DNA methyltransferase 2 (DNMT2) as there is no other DNA methyltransferase encoded in the genome of *S. japonica*, which is different to most plants, animals and unicellular algae such as the green alga *Chlamydomonas reinhartii* (Lopez *et al*., 2015) and even the more closely related diatom *Phaeodactylum tricornutum* (Takuno *et al*., 2016; Tirichine *et al*., 2017). Among the three life‐cycle stages of *S. japonica*, the highest level of DNA methylation was found in both gametophytes. Furthermore, the highest methylated elements of any life‐cycle stage were found to be genes encoding noncoding RNAs (circular and long noncoding), which is different to other photosynthetic eukaryotes as they mostly methylate repetitive elements (e.g. transposons) (Sleutels *et al*., 2002; Dinger *et al*., 2008; Law & Jacobsen, 2010). Differentially methylated genes in MG and in FG were significantly enriched for cellular processes, cell‐wall organization and cell–cell junctions, whereas genes in SP were more enriched for rRNA modification and RNA methylation. However, for all life‐cycle stages, an overall negative correlation was found between DNA methylation and gene expression. Thus, despite an overall low level of cytosine methylation in the genome of *S. japonica*, there is evidence that it may play a significant role for the development of life‐cycle stages and regulation of metabolism via the control of gene expression and noncoding RNAs, which is different to what has been observed so far in plants, animals and many microbes.

## Materials and Methods

### Strain selection, DNA and RNA extraction and purification

The *Saccharina japonica* strain Ye‐c12 was used for methylated DNA immunoprecipitation (MeDIP), bisulfite and RNA sequencing. The haploid male (MG) and female (FG) gametophytes were collected from a blade of the diploid Ye‐c12 sporophyte (SP) and expanded via the application of gametophyte cloning technology (Wang *et al*., 2013). The diploid sporophyte of Ye‐c12 was obtained from sexual reproduction of FG and MG, and collected when the blade length was *c*. 3 cm. Genomic DNA from haploid gametophytes and diploid sporophytes was extracted using the Plant Genomic DNA kit (Tiangen, Beijing, China), and RNA was removed by incubating the DNA solution at 37°C with a DNase‐free RNase A (Tiangen) (Supporting Information Table S1) for 30 min. DNA integrity was assessed using agarose gels and a NanoPhotometer^®^ spectrophotometer (IMPLEN, Westlake Village, CA, USA). DNA was quantified with a Qubit^®^ 2.0 Flurometer (Life Technologies, Carlsbad, CA, USA) using the Qubit^®^ DNA Assay Kit.

Total RNA was extracted using the RNAprep Pure Plant Kit (Tiangen) and DNA was removed using an RNase‐Free DNase I treatment according the instructions by the manufacturer (Tiangen). RNA integrity was assessed on 1% agarose gels and by using the RNA Nano 6000 Assay Kit of the Bioanalyzer 2100 system (Agilent Technologies, Palo Alto, CA, USA). RNA was quantified with a Qubit^®^ 2.0. Flurometer (Life Technologies). A total amount of 3 μg RNA per sample was used as input material. Ribosomal RNA was depleted by Epicentre Ribo‐zeroTM rRNA Removal Kit (Epicentre, NB, USA), and the rRNA‐free residue was cleaned up by ethanol precipitation. Subsequently, libraries were generated using the rRNA‐depleted RNA by NEBNext^®^ UltraTM Directional RNA Library Prep Kit for Illumina^®^ (NEB, USA). The Illumina HiSeq 2500 paired‐end platform was used to sequence the libraries with a read length of 125 bp.

### Identification of genes encoding circular, long noncoding and transfer RNAs (circRNAs, lncRNAs and tRNAs)

Unmapped RNA‐seq reads were kept and 20‐mers from 5′ and 3′ ends of these reads were extracted and aligned independently to reference sequences by bowtie v.2.0.6. Anchor sequences were extended by nd_circ such that the complete reads aligned and the breakpoints were anchored by GU/AG splice sites. The back‐spliced reads with at least two supporting reads were annotated as circRNAs. CNCI (Coding‐Non‐Coding‐Index) (v.2) with default parameters was used to effectively distinguish protein‐coding and noncoding sequences independent of known annotations CPC (Coding Potential Calculator) (0.9‐r2) . mainly was used to assess the extent and quality of the ORF in a transcript and search the sequences with the NCBI eukaryotes’ protein database with the e‐value of ‘1e‐10’. Each transcript was translated in all three possible reading frames and used pfam scan (v.1.3) to identify occurrence of any of the known protein family domains documented in the Pfam database (Pfam A and Pfam B), by default parameters of −E 0.001 –domE 0.001. Transcripts predicted with coding potential by either/all of the three tools above were filtered out, and those without coding potential were the candidate set of lncRNAs. Genome‐wide tRNA genes were predicted using the tRNAscan‐SE web server (Lowe & Chan, 2016). The genomic loci for circRNA, lncRNA and tRNA were deposited at CNGB Nucleotide Sequence Archive (https://db.cngb.org/cnsa/, project CNP0000364).

### Determining the level of heterozygosity between gametophytes and the reference genome

Both parental haploid gametephytes were used to perform whole‐genome sequencing (WGS) employing an Illumina Hiseq 2500 platform, and the reads of each parent were mapping to the reference *S. japonica* genome using BWA (Li & Durbin, 2010). The heterozygosity sites and indel sites were called using samtools (Li *et al*., 2009), which also were used to calculate the heterozygosity between individual haplotypes and the reference genome.

### Methylated DNA immunoprecipitation (MeDIP)

Extracted genomic DNA was sonicated to obtain fragments from 100 to 500 bp. The fragmented DNA was end‐repaired, subjected to A‐tailing and PE adapters were ligated. Subsequently, the treated DNA fragments were immunoprecipitated with an antibody that specifically recognizes 5‐methylcytosine using the MagMeDIP Kit (Diagenode, Liege, Belgium). The specificity of the enrichment was confirmed by quantitative real‐time polymerase chain reaction (qRT‐PCR). After PCR amplification of the enriched fragments, they were quantified with the Agilent 2100 Analyzer (Agilent Technologies). Sequencing libraries of MeDIP fragments were constructed by adopting the Illumina paired‐end protocol. The negative control (input) MeDIP experiment was performed using the same procedure except for the absence of DNA immunoprecipitation with the 5‐methylcytosine recognizing antibody.

### MeDIP‐Seq sequence alignments and data analysis

**Figure 1:**
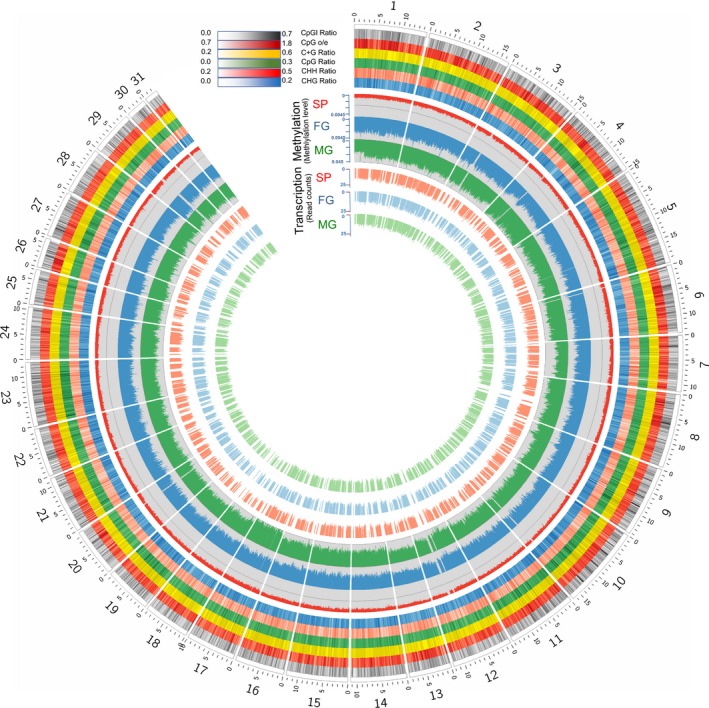
Methylome and transcriptome profiles for 31 chromosomes of the *Saccharina japonica* sporophyte (SP), male (MG) and female (FG) gametophyte genomes. All calculations were conducted by applying a 10‐kbp sliding window. The sliding step length was set at 10 kbp ensuring no region was accounted for twice. The CpGI ratio (grey track), CpG_o/e_ (observed value/expected value, dark red track), C + G Ratio (yellow track), CpG ratio (green track), CHH ratio (light red track) and the CHG ratio (blue track) are displayed by the six outermost circles in the graph. Differential transcription (normalized read counts) between the different life‐cycle stages in relation to DNA methylation (MLgf) is shown by the six innermost circles.

Raw sequencing data were filtered by removing the adaptors and discarding low‐quality reads. Clean reads were aligned to the reference genome of *S. japonica* with bowtie's (Langmead & Salzberg, 2012) best mode using default parameters. Read depths of each sample were normalized to eliminate the influence via differences of total read numbers between samples (Fig. 1a). After using input reads to eliminate the background noise in three MG samples, peaks were called using ‘MACS2 callpeak’ (Zhang *et al*., 2008) (Fig. S2A). According to GTF annotation, the normalized absolute read depth (NARD) between genomic components were calculated, including GENOME (genome‐wide), TSSUP (transcript start site), TESDOWN (transcript stop site), exon, intron, transposable elements (TEs), genes encoding for lncRNAs, circRNAs and tRNAs. Absolute read depths were normalized to genome read depths in order to be comparable between any two classes. Absolute read depth (ARD) for each class of genomic components was calculated as following: ARD = (absolute read counts (ARC) × 150)/total length of this class. Finally, ARDs of each class were normalized to the NARD of its genome (Fig. S2B). The NARDs across the genomic components were represented via metaplot (Fig. S2C). The regions 2 kbp upstream and downstream of the components were split into 20 nonoverlapping bins, whereas genes were split into 40 equal windows.

### Library preparation and quantification of whole‐genome bisulfite sequencing (WGBS)

A total amount of 5.2 μg genomic DNA spiked with 26 ng lambda DNA were fragmented to 200–300 bp by sonication (Covaris S220), followed by end‐repair and adenylation. Cytosine‐methylated barcodes were ligated to sonicated DNA. DNA fragments were treated twice with bisulfite using EZ DNA Methylation‐Gold^TM^ Kit (Zymo Research, Irvine, CA, USA), before the resulting single‐stranded DNA fragments were PCR‐amplified using KAPA HiFi HotStart Uracil + ReadyMix (2×). Libraries were quantified by Qubit^®^ 2.0 Flurometer (Life Technologies) and qRT‐PCR, and the insert size was assessed on the Agilent Bioanalyzer 2100 system.

### Clustering and data analysis of WGBS

Clustering of the index‐coded samples was performed on a cBot Cluster Generation System using TruSeq PE Cluster Kit v.3‐cBot‐HS (Illumina) according to the manufacturer's instructions. After cluster generation, the library preparations were sequenced on an Illumina Hiseq 2500 platform and 125‐bp paired‐end reads were generated. Image analysis and base calling were performed with the Illumina CASAVA pipeline, and finally 125 bp paired‐end reads were generated. bismark software (v.0.16.1) (Krueger & Andrews, 2011) was used to perform alignments of bisulfite‐treated reads to a reference genome using default parameters. Results of the methylation extractor were transformed to bigWig format for visualization using the IGV browser (Thorvaldsdóttir *et al*., 2013). The bisulfite nonconversion rate was calculated as the percentage of cytosines sequenced at cytosine reference positions in the lambda genome.

### Calculation of methylation levels of mCs using WGBS

The methylation level of methylated cytosines (MLmc) was calculated by the read count of methylated cytosines mapped to their genomic locus divided by the read count of all cytosines mapped to the same locus:

$$\text{ML}\left(C\right)=\frac{\text{reads}(\text{mC})}{\text{reads}(\text{mC})+\text{reads}(C)}$$

**Figure 2:**
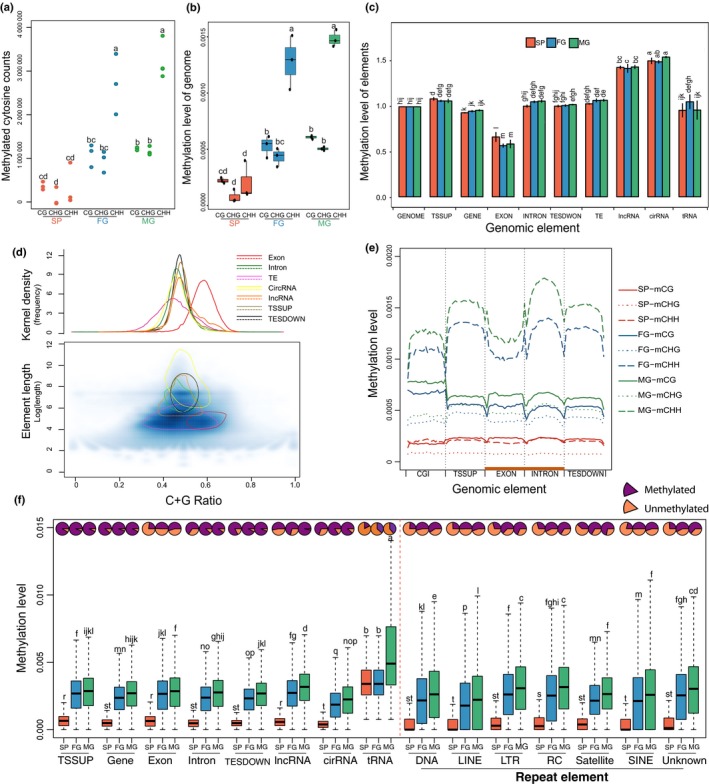
Genome‐wide methylation landscapes and NMLgc of *Saccharina japonica* sporophyte (SP), male (MG) and female (FG) gametophytes. (a) The counts of methylated cytosines (mCs) in CpG, CHG and CHH contexts for SP, FG and MG. The three dots in each column are results of replicates. (b) Distribution of methylation level of the whole genome (MLwg) in CpG, CHG and CHH contexts for SP, FG and MG. See the Materials and Methods section for the calculation of MLwg. The data of methylation level of each mC (MLmc) and its context attribute is obtained from the direct result Bismark (Supporting Information Datasets S1–S9). The white spots of the violin plots stand for the median value. (c) Methylation level of genomic components (MLgc) of TSSUP (transcript start site), TESDOWN (transcript stop site), exon, intron, transposable elements (TEs), genes encoding for long noncoding RNAs (lncRNAs), circular RNAs (circRNAs) and transfer RNAs (tRNAs). To eliminate the variability among samples, all MLgc are normalized by the intrasample MLwg (GENOME). Error bars indicate ± SD. (d) The distribution of CG ratios (number of G and C residues/total number of residues) of all elements for all life stages. Upper panel: density plot lines of different types (in different colours) show the distribution of CG ratios of all element types. The density command was used with parameter bw = 0.01 to draw these plots. Lower panel: scatter plots show the distribution of the lengths of individual elements (*y*‐axis) and their CG ratios (*y*‐axis). (e) Metaplots of methylation levels of genomic fragments (MLgf) across genomic elements of SP, FG and MG in three contexts (CpG, CHG and CHH). Each single element is divided into 20 equal bins and numbered by order of position, and the MLgf for each bin is calculated in three contexts (CpG, CHG and CHH, respectively). The MLgf of bins in the same order in the same genomic class are averaged. (f) Methylation levels of methylated genomic elements (MLgf). The pie charts on top describe the percentage of methylated (purple) and unmethylated (orange) elements, whereas box plots display their Mlgf (regardless of unmethylated genomic elements). Statistics were based on Duncan's and Student's *t*‐tests, the lowercase letters notations associated with plots in (a–c, f) indicate the level of significance among different groups.

**Figure 3:**
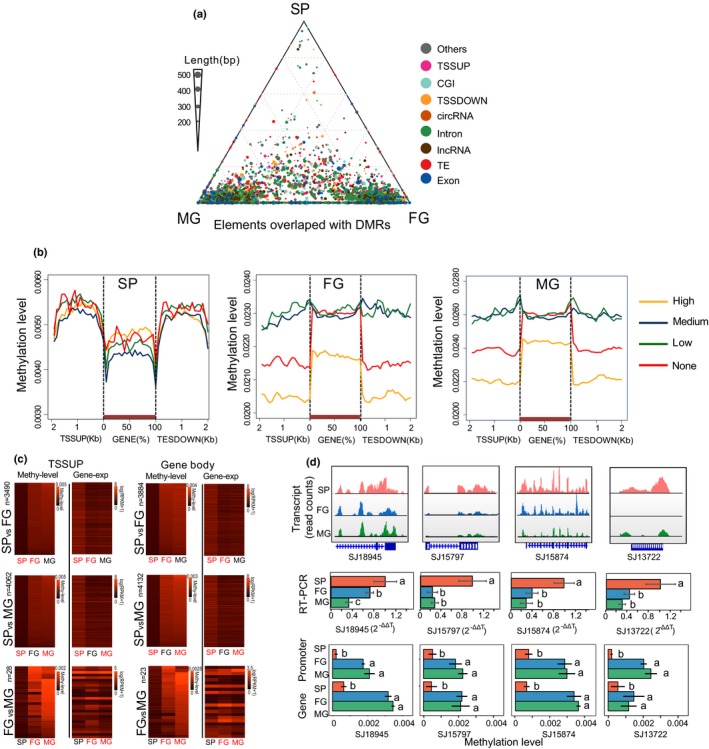
Genomic elements overlapped with differentially methylated regions (DMRs) for all *Saccharina japonica* life‐cycle stages (Sporophyte (SP), male (MG) and female (FG) gametophytes) and their relationship to gene expression. (a) Methylation differences of genomic elements (different colours) that overlapped with DMRs (see the Materials and Methods section) for SP, FG and MG. Ternary plots were drawn according to the methylation levels of genomic fragments (MLgf). (b) MLgf of genes for SP, FG and MG including 2‐kbp up‐ (TSSUP) and down‐stream (TESDOWN) flanking regions. All 18 733 genes were classified into four quantiles according to their expression levels (high, medium, low and none). The 2‐kbp regions up‐ and downstream of genes were divided into 100‐bp intervals for screening. Each gene was divided into 20 intervals (5% of total coding length per interval). Plots show the MLgf of each interval for both coding and noncoding regions up‐ and down‐stream of genes. (c) Heat maps of genes and their promoter regions (TSSUP) with significant negative correlation (*P* < 0.05) between their MLgf and expression levels for pairwise comparisons between all life‐cycle stages. Heat maps are coloured according to MLgf whereas gene expression levels are coloured according to log(RPKM+1). Abbreviations used in the figure: methy‐level (MLgf), gene‐exp (gene expression). (d) Significant negative correlation (*P* < 0.05) between the MLgf and the expression of genes (including their promoter regions) which moved from the sex determination region to autosomal loci. Upper row of panels: transcript read counts of four genes (SJ18945: glycosyl transferase, SJ15975: villin, SJ15874: GTPase activating protein, SJ13722: memo‐like protein) from SP, FG and MG. Middle row of panels: real‐time (RT)‐PCR results of the same four genes using SP as control. Lower row of panels: Methylation levels of the four genes and their promoters. *n* = 3; Statistics were based on Duncan's and Student's *t*‐tests. Error bars indicate ± SD.

The methylation level of a specific genomic fragment (MLgf) (including every single gene, intron, circRNA, lncRNA, transcriptional start (TSSUP) and stop (TESDOWN) CpG island (CGI), tRNA and 10k bin of chromosomes or the entire chromosome) was calculated based on the methylation levels of all mCs of this genomic fragment divided by the sum of cytosines and guanines of the same fragment (Figs 2f, 3) (Xiang *et al*., 2010).

In order to identify genomic compomemts that were preferentially methylated, the methylation level of a class of genomic component (MLgc such as tRNAs, lncRNAs) was calculated as following: the sum of MLmc of all mCs belonging to a specific component (e.g. tRNAs, lncRNAs) divided by the sum of cytosines and guanines of this component. The approach also was used to calculate the methylation level of the whole genome (MLwg; Fig. 2b). To remove experimental artifacts, noise and any batch effects, MLgc were normalized according to MLwg, which is defined as the normalized methylation level of genomic components (NMLgc; Fig. 2c) (Xiang *et al*., 2010).

### Differentially methylated sites (DMSs: a DMS corresponds to a single nucleotide), regions (DMRs) and promoters (DMPs) of WGBS

The DMRs were identified using the swdmr software (http://122.228.158.106/swDMR/), which uses a sliding‐window approach. The window size of 1000 bp using a step length of 100 bp was used. To avoid overlap of neighbouring DMRs, the sliding‐window always started downstream of already identied DMRs. Fisher's exact test was implemented to detect DMRs. The areaStat value that combines both the *P*‐values and fold‐change between two experimental groups were introduced to identify the DMR (see details for areaStat: http://www.bioconductor.org/packages/release/bioc/vignettes/DSS/inst/doc/DSS.html#11_background). A larger absolute value of the areaStat is more likely to be a DMR. The minimal absolute value of areaStat in the present study is 40, resulting in a greater than six‐fold difference in methylation level between two experimental groups (*n* = 3) with *P* < 0.05. Customized scripts were used to identify DMSs using Fisher's exact test with FDR multiple test correction. DMSs with *P* < 0.05 and a fold‐change of > 2 in methylation level between two experimental groups (*n* = 3) were considered candidate DMSs. The identification of DMPs was conducted for 2000 bp upstream of each gene using the same significance criteria as for DMRs.

### Validation of DNA methylation by bisulfite sequencing PCR (BS‐PCR)

The MLgf of specific genomic regions were validated by BS‐PCR. Genomic DNA (500 ng) of MG, FG and SP were converted using EpiTect Fast Bisulfite Conversion Kit (Qiagen) according to the manufacturer's instruction. CpG islands were identified using the MethPrimer (http://www.urogene.org/methprimer/), which also was used to design specific BSP methylation primers. PCRs were carried out with the following program: initial denaturation at 95°C for 30 s followed by 40 cycles of 95°C for 5 s, 60°C for 10 s, and 72°C for 40 s. The PCR products were purified and recovered by a DNA Universal DNA Purification Kit (Tiangen). Next, purified PCR products were cloned into the pMD18‐T vector (Takara, Beijing, China) and transformed into *Escherichia coli* DH5α‐competent cells (Yixin, Shanghai, China) for further replication. Positive clones were screened and sequenced (Sunny, Shanghai, China). Up to 30 randomly picked clones for each amplified locus were sequenced using the ABI3730xl platform.

#### Phylogenetic analysis

Given the large diversity of DNA methyl transferases (DNMTs), only the DNA methylase motif region (PF00145) was used for constructing phylogenetic trees after sequence alignment using muscle 3.8.31 (Edgar, 2004). Maximum‐likehood (ML) phylogentic trees were constructed using mega6 with the LG + G + F model, predicted as best model by the ‘Find best DNA/protein models’ module of mega6 (Tamura, 2013). Motif structures of genes were visualized using the Interactive Tree of Life (iTOL) (Ivica & Peer, 2016). 3D structures of DNMT proteins were visualized using X‐ray structure models from Swiss‐Model https://www.swissmodel.expasy.org/.

### Quantitative real‐time PCR

Total RNA was extracted using TRIzol (Invitrogen) according to the user manual. Purified RNA was dissolved in diethypyrocarbonate (DEPC)‐treated water. cDNA was synthesized from the total RNA using Moloney murine leukemia virus reverse transcriptase (Promega Biotech). The qRT‐PCR reactions were performed with the ABI StepOne Plus Real‐Time PCR System (Applied Biosystems, Foster City, CA, USA) using SYBR Green fluorescence (TaKaRa Bio, Kusatsu, Japan) according to the manufacturer's instruction. The 18S rDNA gene was used as reference. For each of the selected genes, three biological replicates were assayed independently. The qRT‐PCR amplifications were carried out in a total volume of 20 μl containing 10 μl of 26 SYBR Premix Ex TaqTM II (TaKaRa Bio), 0.6 μL (10 mM) of each primer, 2.0 μl of the diluted cDNA mix and 6.8 μl de‐ionized water. The qRT‐PCR protocol was as follows: initial denaturation at 95°C for 30 s followed by 40 cycles of 95°C for 5 s, 60°C for 10 s, and 72°C for 40 s. The 2^−△△CT^ method was used to analyze the qRT‐PCR data.

### Data availability

Datasets S1–S9 and their source Illumina clean data for methylome have been deposited in NCBI's Gene Expression Omnibus and are accessible through GEO Series accession no. GSE117191 (https://www.ncbi.nlm.nih.gov/geo/query/acc.cgi?acc=GSE117191). The Illumina raw data for transcriptomes have been deposited at the NCBI database (the SRA accession numbers are SRR5860561–SRR5860568). The Illumina data of the MeDIP‐seq experiments and the WGS data were deposited at CNGB Nucleotide Sequence Archive (https://db.cngb.org/cnsa/, with project accession no. CNP0000364), as were the reassembled genome and genomic loci for TEs, CGIs, genes, circRNAs, lncRNAs, tRNAs and the detailed methylation data for genome‐wide visualization (visualization data is also deposited in GEO Series accession no. GSE117191). The methylation data based on WGBS and the gene expression based on RNA‐seq can be visualized using the online genome browser at the OrcAE database (https://bioinformatics.psb.ugent.be/orcae/).

## Results

### Assessment of DNA methylation based on an improved genome assembly

In order to obtain evidence of cytosine methylation in the genome of *S. japonica,* MeDIP was performed using a mixture of DNA from all three life stages of the *S. japonica* strain Ye‐c12 (SP, FG, MG). Methylated DNA was recognized by a 5‐methylcytosine antibody. After PCR amplification, 220–520‐bp fragments were selected to construct the sequencing libraries. The libraries revealed a positive signal of the excepted length (220–520 bp; Fig. S1A), indicating the presence of 5‐methylcytosines in the DNA of *S. japonica*. However, the positive control (Bos DNA) gave near six‐fold stronger signals (Fig. S1B), suggesting that the concentration of 5‐methylcytosines was significantly higher in Bos DNA (average MLwg of 8.43%) compared to *S. japonica* (Dechow & Liu, 2018).

In order to provide first insights into the methylome landscape on a chromosome‐wide scale, pseudo‐chromosomes were constructed by combining a genetic linkage map (Zhang *et al*., 2015) with scaffold information. A total of 1576 scaffolds were anchored to 31 linkages of the genetic map (Fig. 1; Methods S1, part 1), accounting for 64.69% (35 293 Mb) of the assembled kelp scaffolds. The remaining scaffolds were concatenated to construct an artificial chromosome (Figs S2, S3; Methods S1, part 1).

### Cytosine methylome landscapes based on MeDIP‐seq

In order to obtain insights into the global methylation landscape between life stages, independent MeDIP experiments were performed for FG, MG and SP with three biological replicates each. However, only MG produced enough immunoprecipitated DNA after PCR amplification. The negative results for FG and SP were verified by additional MeDIP experiments, suggesting reduced levels of methylation in FG and SP. Then WGB‐seq were performed for three MG replicates and one negative control (also named as input). A total of 20 Gbp paired‐end reads were generated from three MG MeDIP‐seq libraries and one input library (Table S1). The MeDIP‐seq reads were mapped across chromosomal regions in three MG and one control sample (Fig. S2A); 8824 peaks were obtained in a total length (three replicates) of 8 764 952 bp, covering 1.61% of the genome (Table S2). The distribution of MeDIP‐seq reads (normalized absolute read depth = NARD) showed that the reads were distributed across all of the genomic feature regions (Fig. S2B). Interestingly, lncRNA genes and cirRNA genes had the highest level of NARDs (*P* < 0.05), whereas exons and genes encoding tRNAs had the lowest level of methylation (*P* < 0.05). The distribution of NARDs across lncRNA genes and CpG islands (CGIs) had a higher level of methylation than regions 2 kbp up‐ and downstream (Fig. S2C).

### Cytosine methylome landscapes based on WGBS

Overall, only *c*. 1.4% of cytosines in the genome of *S. japonica* were methylated based on WGBS. At such low levels of DNA methylation, a few sequencing errors can significantly impact the results. Thus, to minimize false positives and negatives in the present dataset, three biological replicates were sequenced for each life‐cycle stage of *S. japonica* using WGBS (Fig. 1; Table S1). By calculating the percentage of cytosines sequenced at cytosine reference positions in the lambda genome, a final conversion rate of > 99.95% was identified for unmethylated cytosines. By combining nine individual methylome profiles, it was possible to obtain a high‐quality, high‐resolution kelp methylome with a final sequence yield of 92.4 Gbp covering 86% of all cytosines in the genome (Table S1). To minimize the number of false positives, the lower threshold for identification of methylated cytosines was set at four sequence reads per methylated cytosine (Table S1; Datasets S1–S9).

Most of the chromosomes had the highest MLgf in MG with additional hypermethylated chromosomes 4, 17 and regions on chromosomes 9, 12, making MG the most methylated life‐cycle stage, followed by FG and SP (Figs 1, S4, S5B; Table S3). Although chromosomes 7 and 29 had a higher MLgf in FG compared to MG (Figs 1, S4, S5B), sex‐determining genes were not found to be localized on these chromosomes (Lipinska *et al*., 2017) (Tables S3–S4). Approximately 57% of the methylated cytosines in the genome were methylated in a CHH context, whereas 19% and 24% were in CHG or CpG contexts, respectively (Fig. 2a; Table S4). Even though more cytosines were methylated in a CHH context, the median methylation level of methylated CpG (MLmc) is highest for all three contexts. (*P* < 0.05; Fig. S6). Interestingly, MLgfs in 10 000 bps bins of chromosomes show a significantly negative correlation with the CpG O/E (observed/excepted value), CpG ratio (nucleotide counts of all CpGs in bin/bin length) and CGI (CpG Island) ratio (nucleotide counts of all CGIs in bin/bin length), and a positive correlation with the CHH ratio (nucleotide counts of all CHHs in bin/bin length) (Figs S7–12), indicating that the CHH sites are preferentially methylated.

Among all life‐cycle stages, SP had the lowest counts of methylated cytosines and the lowest MLwg for the whole genome and MLgc for all classes of components (CpG, CHG and CHH) (*P* < 0.05; Figs 2a,b, S6, S13). However, NMLgc fluctuated significantly across the genome (Fig. 2c), reflecting a mosaic methylation pattern where nonmethylated regions were interspersed with methylated regions. Notably, the most highly methylated genetic elements in the genome of *S. japonica* were loci encoding for circRNAs and lncRNAs (Figs 2c, S14; Tables S5–S15) with 81% and 66% of all elements methylated, respectively (Fig. 2f; Tables S4–S14). Coding genes and their regulatory parts (TSSUP and TESDOWN) sites) were significantly less methylated than noncoding circRNA and lncRNA genes (*P* < 0.05; Fig. 2c). Exons had the lowest NMLgc (*P* < 0.05; Fig. 2d). Most of the exons were methylated in the CHH context with a concave methylation pattern. Introns, however, showed a convex methylation pattern (Figs 2e, S15). Convex methylation patterns also were found in the core regions of genes encoding for lncRNAs and circRNAs (Fig. S15).

Predicted numbers of tRNA genes for the mitochondrion, chloroplast and nuclear genomes of *S. japonica* were 24, 29 and 757*,* respectively. On average, 29.1% of all tRNA genes were methylated (Fig. 2f; Table S14). In plants and algae, repetitive elements such as TEs are often most highly methylated (Cokus *et al*., 2008; Law & Jacobsen, 2010; Su *et al*., 2014; Takuno *et al*., 2016). However, in *S. japonica*, methylation of TEs does not seem to play a significant role (Figs. 2c, S12–S13), as only 47.8% TEs methylated, which is lower compared to genes (93.1%), introns (81.3%), circRNAs (80.9%), lncRNAs (66.4%), TSSUPS (90.3%), TESDOWNS (89.6%) and CGIs (60.5%), but just a little higher than exons (42.4%) and tRNA genes (29.1%) (Fig. 2f; Tables S5–S14). Although only 42% (Table S7) of exons were methylated, methylated exons had above average MLgf (Fig. 2f).

### Functional analysis of DMRs between life‐cycle stages

Identification of DMRs was done using a sliding‐window approach with a window size of 1000 bp and a step length of 100 bp (see the Materials and Methods section). Despite the overall low level of DNA methylation in *S. japonica*, the number of DMRs varied considerably between life‐cycle stages. By systematically surveying the regions that were differentially methylated between all life‐cycle stages, 12 hypermethylated DMRs were found in sporophytes (Table S15a,d), 646 hypermethylated DMRs in female (Table S15b, e) and 790 hypermethylated DMRs in male gametophytes (Table S15c, f). DMRs were identified to overlap with exons, introns, TEs, TSSUPs, TESDOWNs, circRNA genes, lncRNA genes and intergenic DNA, and the length of overlapping fragments ranged from 10 to 500 bp (Fig. 3a). By clustering the overlapped elements with DMRs according to their MLgf for each life‐cycle stage, they were found to be most highly methylated in MG and FG (Fig. 3a), reflecting the genome‐wide trend (Fig. 2a,b). GO and KEGG enrichments were conducted for all DMRs and all life‐cycle stages representing protein‐coding genes (Table S16). Differentially methylated genes (genes overlapped with DMRs) in SP were enriched in rRNA modification (GO:0000154), RNA methylation (GO:0001510), generation of precursor metabolites and energy (GO:0006091), methyltransferase activity (GO:0008168), transferase activity (GO:0016758, GO:0016758) (Table S15). Differentially methylated genes in MG were enriched for the following GO terms: cellular process (GO:0009987), cell wall organization (GO:0071555), cellular metabolic process (GO:0044237), metabolic process (GO:0008152) and Pentose phosphate pathway (ko00030) (Figs S16–18; Tables S16–17). Differentially methylated genes specific to FG were enriched for GO terms of cell–cell junctions (GO:0005911), transition metal ion binding (GO:0046914), cellular developmental processes (GO:0048869) and plant hormone signal transduction pathways (ko04075) (Figs S16–18; Tables S16–17).

### DNA methylation and gene expression

A greater number of highly expressed genes were identified in sporophytes than in gametophytes (RPKM > 10). Generally, there was a negative correlation between gene expression and MLgfs (Figs 3b, S19). In SP, 3884 genes and 3490 TSSUPs were significantly hypomethylated and more highly expressed than in FG, whereas 4132 genes and 4062 TSSUPs were significantly hypomethylated and more highly expressed than in MG (Duncan's and Student's *t*‐test, *P* < 0.05; Fig. 3c; Tables S17–19). Only 51 genes in SP were hypermethylated with significantly lower expression than in either FM or MG (Student's *t*‐test, *P* < 0.05; Fig. 3c; Tables S18–20). In particular, some of the hypomethylated and highly expressed genes in SP with their respective GO term categories in comparison to FG and MG revealed some insights into processes that might be important for developing fully grown kelp sporophytes (Tables S21–22). For instance, enriched GO terms for this category in SP vs FG included structural molecule activity (GO:0005198, *P* < 0.01) and carbohydrate derivative binding (GO:0097367, *P* < 0.05). The same category for SP vs MG includes the GO term membrane protein complex (GO:0098796, *P* < 0.01). Interestingly, four genes that were reported to have moved from sex‐determining chromosomal regions to auto chromosomes (Lipinska *et al*., 2017) were more highly expressed in SP and had significantly reduced MLgf in all life‐cyle stages (Figs 3d, S20; Table S23; Methods S1, part 2). Other hypomethylated and strongly expressed genes in SP included a cellulose synthase, a mannuronate C5‐epimerases (MC5E) and an iodoperoxidase (vIPO) (Fig. S21).

Although there was no significant correlation between MLgf and expression levels of lncRNAs and circRNAs genes (Figs S22–23) (|Pearson correlation index| < 0.01, *P* > 0.05), lncRNAs genes with expression levels in the top 10% had the lowest level of MLgf, and lncRNAs with MLgf in the top 10% had the lowest expression levels. The similar trends also were found in circRNAs. However, there was no significant correlation between MLgf and the expression of TEs (Fig. S24).

### A comparison between MeDIP‐seq, BS‐PCR and WGBS

Evidence for DNA methylation in *S. japonica* although at a relatively low level is provided by MeDIP‐seq and WGBS. Furthermore, there is a high consistency between results from both methods (Pearson correlation index > 0.5, *P* < 0.001; Fig. S25). For example, both methods revealed independently that methylation preferentially took place at genes encoding lncRNAs and circRNAs. These results were confirmed by BS‐PCR with subsequent cloning and Sanger sequencing. Ten genes (Figs 3d, S21) were assessed and good agreement was obtained between the WGBS and BS‐PCR (Figs S26–35). Thus, the independent validation of DNA methylation based on three different methods suggests that the DNA of *S. japonica* indeed appears to be methylated albeit a low level.

### Genome‐wide heterozygosity and its influence on estimating the level of DNA methylation

The level of heterozygosity between the reference genome and the DNA obtained from the individuals used for the experiments can have an influence on estimating methylated cytosines. This is particularly critical if the overall level of methylation is low at a considerable level of heterozygosity between the reference genome and the genome of the individuals used for the experiments. To address this potential issue, Illumina WGS was performed for both male (MG) and female (FG) parents to determine differences in the level of heterozygosity. After mapping *c*. 50 Gbp of FG and MG to the reference genome, it was calculated that the overall level of heterozygosity was ≤ 0.14% (Table S24). Of all identified heterozygosity sites, C‐T/G‐A polymorphic sites (168 560 for FG and 118 421 for MG) overlapped with only 0.02% ~–0.04% of all mCs site for FG and MG samples (Table S24b), indicating that heterozygosity was not impacting > 0.04% of all identified mCs in FG and MG.

### DNA methyltransferases and demethylases in *S. japonica*

**Figure 4:**
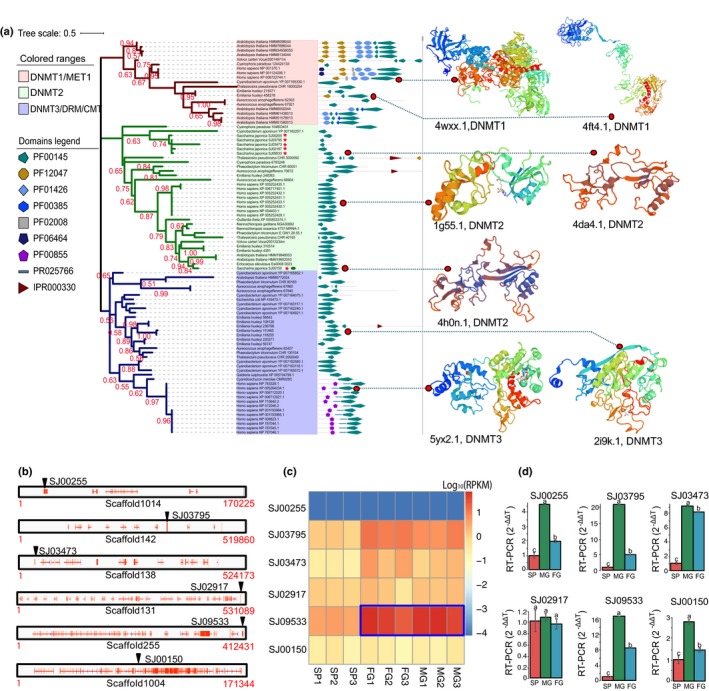
DNA methyltransferases (DNMTs) and their evolution and expression in *Saccharina japonica* life‐cycle stages (Sporophyte (SP), male (MG) and female (FG) gametophytes) (a) Phylogeny of DNMTs including characteristic 3D structures for members of each clade. DNMTs of *S. japonica* were marked using red pentagrams in the tree. Green ellipse marked gene SJ00150 were identified to maintenance chloroplast targeting signal peptide. Homologous X‐ray structure models in SWISS‐MODEL (https://www.swissmodel.expasy.org/) were used to compare the 3D structure of DNMTs 1–3. Seven X‐ray structure models (PDM accession nos. 4wxx.1, 4ft4.1, 1g55.1, 4da4.1, 4h0n.1, 5yx2.1, 2i9k.1) were used as they had the highest coverage and similarity to seven genes marked by red points in the tree. (b) Scaffold locations of DNMT2s in *S. japonica*. Red marks represent genes in scaffolds, and the black triangle indicates the scaffold location of DNMTs. Red numbers indicate the start and end sites of the scaffolds. (c) Heat‐map showing the expression levels of DNMT2s for each life‐cycle stage (SP, FG and MG) and biological replicates (*n* = 3). A significant positive correlation (*P* < 0.05) between the level of DNA methylation and the expression of DNMT2s was shown for FG and MG (blue frame). (d) Quantitative (q)RT‐PCR result of the six DNMT2s for SP, MG and FG. *n* = 3; Statistics were based on Duncan's and Student's *t*‐tests, error bars indicate ± SD, the lowercase letters notations associated with plots in (d) indicate the level of significance among different groups.

The DNMTs and demethylases (DNDMTs) are involved in the establishment of tissue and cell‐type‐specific methylation patterns during developmental processes in most multicellular organisms (Meissner *et al*., 2008; Kohli & Zhang, 2013; Satgé *et al*., 2016; Iurlaro *et al*., 2017). As the overall DNA methylation was relatively low in *S. japonica*, a homology‐based search was performed in the *S. japonica* genome to identify all possible DNMTs and DNDMTs (Figs S36–40). Although the DNA‐methylase domain PF00145 is common in all DNMTs, effective DNA methylation requires the following additional domains, which occur only in the DNMT1/3/4/5/6 family: ADD (IPR025766), PWWP (PF00855), PHD (IPR001965) and a bromo‐domain (PF00439) (Ponger & Li, 2005). All of the six DNMTs found in *S. japonica* had the highest similarity to members of the DNMT2 family without any of the domains required for efficient DNA methylation (Figs 4a,b, S38). There were no homologues found with significant similarity to any of the other DNMTs such as 1, 3, 4, 5 or 6 (Fig. S37). Interestingly, ADD, PWWP, PHD and bromo‐domains were found at other loci in the genome of *S. japonica*, but none of them were found to be DNMT2 (Figs S39–40).

In order to identify proteins involved in active DNA demethylation (DNDMTs), a search was carried out for RRM‐fold domains (PF15628), DNA glycosylase domains (IPR011257), DME, DML, ROS, TET and MBD domains, all of which were reported previously to catalyse the removal of the 5mC base (Meissner *et al*., 2008; Kohli & Zhang, 2013; Su *et al*., 2014; Lipinska *et al*., 2017; Teng *et al*., 2017). Only one potential candidate was found in the genome, which included a DNA glycosylase and the methyl‐CpG‐binding domain protein 2 (MBD2, PF01429) (Figs S41–42). The MBD2 domain was reported to catalyze the removal of a methyl group (Wu & Zhang, 2014).

To have six genes from the DNMT2 family encoded in the *S. japonica* genome with all other DNMTs missing may indicate that DNMT2 methylates DNA in the structural context of tRNAs (Kaiser *et al*., 2017). Thus, the MLgf of tRNA genes encoded in the mitochondrial, chloroplast and nuclear genomes of *S. japonica* were analyzed for all three life stages individually (Fig. S43; Table S25). Overall, the sporophyte had the lowest MLgf of tRNA genes for all three genomes. However, tRNA genes of the nuclear genome of the sporophyte were significantly more methylated than in the organellar genomes, which was not noticeable in the other life stages as tRNA genes in their organellar genomes were much more methylated than their counterparts in sporophytes. The ratio between unmethylated to methylated tRNA genes is highest for SP (Fig. S43). The MLgf of tRNA genes in the nucleus was positively correlated with the MLgf of nucelar genes only for MG. These results suggest possible DNMT2‐mediated DNA methylation, with RNAs as natural substrates of DNMT2 acting as guides as shown previously (Kaiser *et al*., 2017).

## Discussion

Differential DNA methylation is the hallmark of cell‐type specific development and life‐cycle regulation in multicellular organisms (Law & Jacobsen, 2010; Takuno *et al*., 2016). For most multicellular organisms, DNMT2 is not considered to be the enzyme for DNA methylation as it primarily catalyzes transfer (t)RNA methylation and only has very weak DNA methyltransferase activity (Kunert *et al*., 2003; Goll *et al*., 2006; Shanmugam *et al*., 2014; Francesca *et al*., 2015; Kaiser *et al*., 2017). However, recent data (Kaiser *et al*., 2017) have provided the first *in vitro* evidence that DNMT2 can efficiently methylate DNA when DNA fragments are presented as covalent DNA‐RNA hybrids in the structural context of tRNAs. Thus, methylated tRNAs can serve as substrates for methylating DNA presented as covalent hybrids with RNA. Although the data herein do not provide direct evidence for this process to be responsible for DNA methylation *in vivo* in *Saccharina japonica*, it is intriguing to see that there are significantly more methylated tRNA genes in both gametophytes which might serve as a substrate to methylate genes via DNMT2. In male gametophytes (MG), a significantly positive correlation between the methylation level of a specific genomic fragment (MLgf) of tRNA genes and both the expression level of DNMT2s (Fig. 4c,d) and the MLgf of genes corroborates this assumption (Fig. S43). However, if DNMT2 does indeed methylate the DNA of *S. japonica in vivo*, it is being done inefficiently as the overall DNA methylation level (of methylated cytosines (MLmc) and the whole genome (MLwg)) is still very low even though six different DNMT2s are encoded in the genome and expressed. DNMT2 appears to have evolved from a DNA methyltransferase precursor, which is considered an evolutionary ‘relict’ in terms of biochemical catalysis (Jurkowski & Jeltsch, 2011; Raddatz *et al*., 2013) and therefore might explain its inefficiency compared to other DNMT enzymes such as 1 and 3. Although it is possible to have missed DNMTs because of gaps in the genome assembly, the fact that multiple DNMT2 genes are encoded in the *S. japonica* genome, and the positive correlation of their expression with an elevated level of C5 DNA methylation (Pearson correlation test, *P* < 0.05) makes DNMT2s likely candidates for C5 DNA methylation. Support for the role of DNMT2 in C5 DNA methylation comes from reverse‐genetics studies with *Drosophila melanogaster* where knockdown and overexpression of DNMT2 resulted in lower and higher levels of 5 methylcytosine in embryos, respectively (Kunert *et al*., 2003). Likewise, human DNMT2 was shown to have a residual DNA C5 methyltransferase activity in a particular sequence context (Hermann *et al*., 2003).

Although the overall DNA methylation level in *S. japonica* is lower than observed for most multicellular organisms with complex life cycles (Takuno *et al*., 2016), DNA methylation still appears to contribute to the regulation of gene expression and particularly noncoding regulatory RNAs. Furthermore, significant differences between life‐cycle stages suggest that differential DNA methytlation is involved in their formation, which is similar to other multicellular organisms although most of them have much higher and more dynamic levels of DNA methylation (Lopez *et al*., 2015; Willing *et al*., 2015; Gaunt *et al*., 2016; Kawakatsu *et al*., 2016; Mayasich *et al*., 2016; Panikar *et al*., 2017).

Another striking difference is that genome‐wide patterns and mechanisms of cytosine methylation in *S. japonica* are neither more similar to plants or animals, nor similar to other stramenopile species for which single‐base methylome data are available such as *Phaeodactylum tricornutum* (Veluchamy *et al*., 2013). In many photosynthetic organisms including plants, green algae and diatoms, transposable elements (TEs) are the main target of cytosine methylation whereas animals methylate genes and TEs more equally (Cokus *et al*., 2008; Law & Jacobsen, 2010; Takuno *et al*., 2016; Tirichine *et al*., 2017). However, in *S. japonica,* the percentage of methylated TEs is < 48% (Table S5), which is lower than the percentage of methylated genes (93% Table S6). Surprisingly, the highest methylated elements in *S. japonica* were found to be genes encoding circular and long noncoding RNAs (circRNAs and nlcRNAs) (*P* < 0.05 in female gametophytes (FG) and MG; Figs 2c, S14). Significant differences in methylation of both groups of genes encoding noncoding RNAs between sporophytes (SP), FG and MG indicate that they appear to have a role in regulating life‐cycle stages of *S. japonica*, which, to the best of our knowledge, has not been observed before in any organism (Figs S22–23). Further differences to plants and animals have been revealed by comparing mechanisms of DNA methylation. Animals mainly methylate cytosines in the CpG context, whereas plants methylate CHG and CHH sites and many algae methylate in the CpG context (Cokus *et al*., 2008; Lopez *et al*., 2015). *Saccharina japonica* methylates cytosines mainly in the CHH context with much lower but almost equal methylation of CHG and CpG sites.

The fact that a significant number of genes is methylated in *S. japonica,* and that the level of their methylation is negatively correlated with gene expression is in common with many plants and animals. Hence, there is evidence for methylation‐regulated gene expression. Furthermore, methylated genes in MG and FG were negatively correlated with gene expression (Fig. 3b). GO enrichment for these genes revealed that many were involved in transport, membrane stucture and function (Tables S16, S17), which suggests that: these processes are under epigenetic control in MG; and that they are downregulated in comparison to SP and FG. Interestingly, methylated genes in SP and FG were enriched, for example, for small molecule metabolic process and organo‐nitrogen compound metabolic processes. Hypomethylated and highly expressed genes involved in morphogenesis (e.g. MC5E, cellulose synthase) and halogene metabolism (e.g. vIPO) were found in SP (Fig. S21), which suggests that cytosine methylation contributes to regulating fundamental processes underpinning cell differentiation, growth and stress response (Pear *et al*., 1996; Colin *et al*., 2005; Ye *et al*., 2015; Inoue *et al*., 2016). Genes of the sex‐determination system do not show a negative correlation between their level of methylation and gene expression (Fig. S20), suggesting that 5mC DNA methylation might not significantly contribute to their regulation. Interestingly, the four genes that are characterized by different protein domains (MEMO, glycosyltransferase, RING‐type zinc finger, Rab‐GTPase‐TBC) and which moved from the sex‐determination locus in the genome to the autosomal loci were hypomethylated and highly expressed in SP (Lipinska *et al*., 2017) (Fig. S20).

The first single‐base methylome study of a brown alga has revealed that cytosine methylation might be mediated by DNMT2. Although the overall level of DNA methylation is low, it appears to play a significant role in these multicellular algae mainly for controlling regulatory elements (noncoding RNAs) and the expression of genes. Significant differences in overall DNA methylation and methylation‐mediated gene expression between life‐cycle stages suggests that cytosine methylation is involved in regulating growth, tissue differentiation, and several different physiological responses. However, it remains to be seen if DNMT2 indeed is responsible for DNA methylation in *S. japonica*.

## Author contributions

NHY designed the project with contributions from TM, LT and XF; WTH, LHT, PJ, XWZ, DX, CHW, CL and YTW performed the research; XF, NHY, WTH, TM, LT, CL and LHT analyzed the data; TM wrote the manuscript with contributions from XF, NHY, and LT; MP, MJSK and XL revised and edited the manuscript. All authors read and approved the manuscript before submission. The authors declare no conflict of interest.

## Supporting information

Please note: Wiley Blackwell are not responsible for the content or functionality of any Supporting Information supplied by the authors. Any queries (other than missing material) should be directed to the *New Phytologist* Central Office.

**Fig. S1** MeDIP‐seq libraries.
**Fig. S2** Methylation profiles of MG based on MeDIP‐seq.
**Fig. S3** Profiles of SP, MG and FG methylomes and transcriptomes of Chromosome 0.
**Fig. S4** Chromosome‐specific profiles for SP, MG and FG methylomes and transcriptomes.
**Fig. S5** GC characteristics and methylation levels of chromosomes.
**Fig. S6** Comparison between methylation levels of individual cytosines.
**Fig. S7** Correlation between CpGo/e and the methylation level.
**Fig. S8** Correlation between C+G percentage and the methylation level.
**Fig. S9** Correlation between CpG percentage and the methylation level.
**Fig. S10** Correlation between CHG percentage and the methylation level.
**Fig. S11** Correlation between CHH percentage and the methylation level.
**Fig. S12** Correlation between CpG islands ratio and the methylation level.
**Fig. S13** Difference in methylation levels for different types of genomic elements between life‐cyle stages.
**Fig. S14** Normalized methylation level for genomic elements.
**Fig. S15** Methylation for all methylation contexts of lncRNA genes and cirRNA genes.
**Fig. S16** Most enriched (top 20) KEGG pathways for methylated genes in FG.
**Fig. S17** Most enriched (top 20) KEGG pathways for methylated genes in MG.
**Fig. S18** Most enriched (top 20) KEGG pathways for methylated genes in SP.
**Fig. S19** Genome‐wide gene expression profiling and DNA methylation profiles.
**Fig. S20** Methylation profiles of the sex‐determining region.
**Fig. S21** Base‐specific methylation and transcriptional coverage of selected metabolic genes.
**Fig. S22** Correlation between MLgf and gene expression for lncRNA genes.
**Fig. S23** Correlation between MLgf and gene expression for circRNA genes.
**Fig. S24** The correlation ship between MLgf of TEs and their expression level.
**Fig. S25** Consistency assessment of MeDIP‐seq vs WGBS data.
**Fig. S26** BS‐PCR for SJ18945.
**Fig. S27** BS‐PCR for SJ15797.
**Fig. S28** BS‐PCR for SJ15874.
**Fig. S29** BS‐PCR for SJ13722.
**Fig. S30** BS‐PCR for SJ21435.
**Fig. S31** BS‐PCR for SJ22030.
**Fig. S32** BS‐PCR for SJ15673.
**Fig. S33** BS‐PCR for SJ19510.
**Fig. S34** BS‐PCR for SJ22283.
**Fig. S35** BS‐PCR for SJ20815.
**Fig. S36** Phylogeny of methytransferases based on Pfam PF00145.
**Fig. S37** Domain architecture of DNMT4/5/6.
**Fig. S38** DNA methylase domain PF000145 and their expression profile.
**Fig. S39** Gene structure, expression patterns and chromosomal locations of MBT, PWWD, DNMT1, Zinc finger PHD‐type.
**Fig. S40** Gene structure, expression patterns and chromosomal locations of ADD, Pre_SET, SET, SNF, helicase and AP2/ERF.
**Fig. S41** Gene structure and phylogeny of demethyltransferases.
**Fig. S42** Gene structure of MBD domains and their phylogeny.
**Fig. S43** Methylation levels of tRNA genes.

**Table S1** Summary of methylation and RNA sequencing data and mapping results.

**Table S2** Peak‐specific information.

**Table S3** Chromosome‐specific gene methylation and expression.

**Table S4** Features of methylated sites for all methylation contexts and life‐cycle stages.

**Table S5** Features of TE methylation.

**Table S6** Features of gene methylation.

**Table S7** Features of exon methylation.

**Table S8** Features of intro methylation.

**Table S9** Features of lncRNA gene methylation.

**Table S10** Features of circle RNA gene methylation.

**Table S11** Features of TSSUP methylation.

**Table S12** Features of TESDOWN methylation.

**Table S13** Features of CGI methylation.

**Table S14** Features of tRNA gene methylation.

**Table S15** Information of DMRs.

**Table S16** GO enrichment of genes located in DMRs.

**Table S17** KEGG enrichment of the genes located in DMRs.

**Table S18** Expression and statistical tests of all genes and life‐cycle stages.

**Table S19** Methylation level and statistical tests of all TSSUPs and life‐cycle stages.

**Table S20** Methylation level and statistical tests of all genes and life‐cycle stages.

**Table S21** KEGG enrichment of genes and TSSUPs for Fig. 3(c).

**Table S22** GO enrichment of genes and TSSUPs for Fig. 3(c).

**Table S23** Methylation and expression of genes located in the sex‐determining‐region for all life‐cyle stages.

**Table S24** Heterozygosity between FG and MG vs the reference genome.

**Table S25** Methylation levels of tRNA genes.

**Methods S1** 1, Constructing the pseudo‐chromosomes using a genetic linkage map; 2, methylation profiles of sex‐determining regions and their gene expression; 3, DNA methyltransferases evolution and their putative function; 4, other methods in this article.
