# Supplementary material for: Single‐base methylome profiling of the giant kelp Saccharina japonica reveals significant differences in DNA methylation to microalgae and plants
Source: New Phytol. 2019 Sep 27;225(1):234–49. doi: 10.1111/nph.16125 (PMC6916402; doi:10.1111/nph.16125)
Supplement: Supplementary file 1 — Fig. S1 MeDIP‐seq libraries. Fig. S2 Methylation profiles of MG based on MeDIP‐seq. Fig. S3 Profiles of SP, MG and FG methylomes and transcriptomes of Chromosome 0. Fig. S4 Chromosome‐specific profiles for SP, MG and FG methylomes and transcriptomes. Fig. S5 GC characteristics and methylation levels of chromosomes. Fig. S6 Comparison between methylation levels of individual cytosines. Fig. S7 Correlation between CpGo/e and the methylation level. Fig. S8 Correlation between C+G percentage and the methylation level. Fig. S9 Correlation between CpG percentage and the methylation level. Fig. S10 Correlation between CHG percentage and the methylation level. Fig. S11 Correlation between CHH percentage and the methylation level. Fig. S12 Correlation between CpG islands ratio and the methylation level. Fig. S13 Difference in methylation levels for different types of genomic elements between life‐cyle stages. Fig. S14 Normalized methylation level for genomic elements. Fig. S15 Methylation for all methylation contexts of lncRNA genes and cirRNA genes. Fig. S16 Most enriched (top 20) KEGG pathways for methylated genes in FG. Fig. S17 Most enriched (top 20) KEGG pathways for methylated genes in MG. Fig. S18 Most enriched (top 20) KEGG pathways for methylated genes in SP. Fig. S19 Genome‐wide gene expression profiling and DNA methylation profiles. Fig. S20 Methylation profiles of the sex‐determining region. Fig. S21 Base‐specific methylation and transcriptional coverage of selected metabolic genes. Fig. S22 Correlation between MLgf and gene expression for lncRNA genes. Fig. S23 Correlation between MLgf and gene expression for circRNA genes. Fig. S24 The correlation ship between MLgf of TEs and their expression level. Fig. S25 Consistency assessment of MeDIP‐seq vs WGBS data. Fig. S26 BS‐PCR for SJ18945. Fig. S27 BS‐PCR for SJ15797. Fig. S28 BS‐PCR for SJ15874. Fig. S29 BS‐PCR for SJ13722. Fig. S30 BS‐PCR for SJ21435. Fig. S31 BS‐PCR for SJ22030. Fig. S32 BS‐PCR for SJ15673. Fig. [file NPH-225-234-s001.pdf]

New Phytologist Supporting Information

Single-base methylome profiling of the giant kelp Saccharina japonica reveals significant differences in DNA methylation to microalgae and plants

Xiao Fan, Wentao Han, Linhong Teng, Peng Jiang, Xiaowen Zhang, Dong Xu, Chang Li, Matteo Pellegrini, Chunhui Wu, Yitao Wang, Michelle Joyce Slade Kaczurowski, Xin Lin, Leila Tirichine, Thomas Mock, Naihao Ye

Acceptance date: 6 August 2019

Fig. S1

A

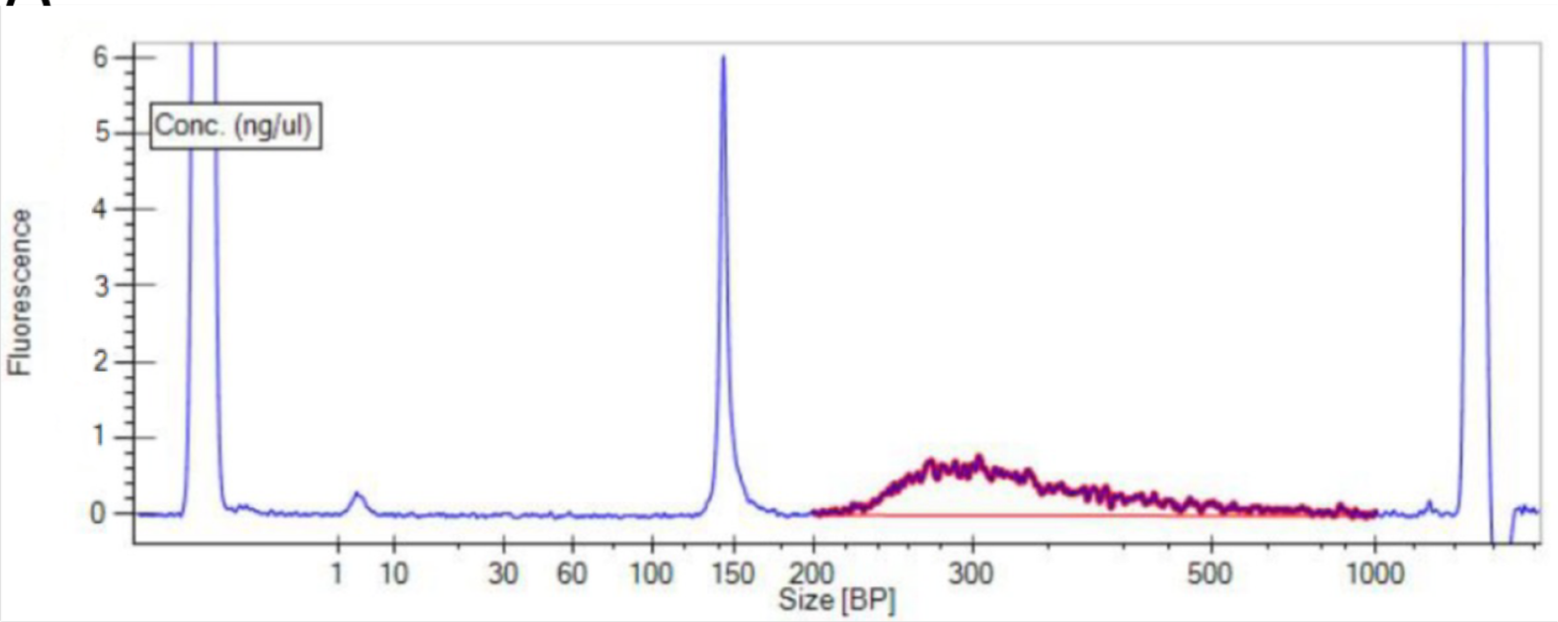

Conc. (ng/ul)
Fluorescence
6
5
4
3
2
1
0
1
10
30
60
100
150
200
300
500
1000
Size [BP]

B

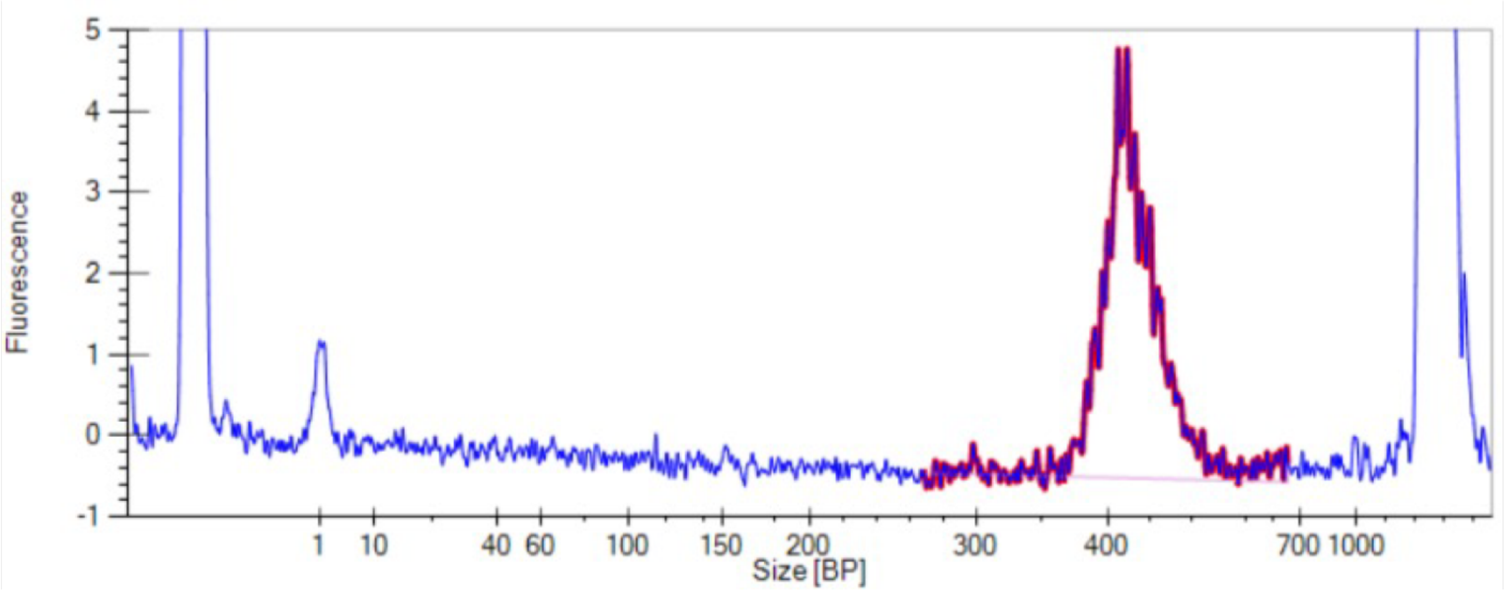

Fluorescence
5
4
3
2
1
0
-1
1
10
40
60
100
150
200
300
400
700
1000
Size [BP]

Fig. S2

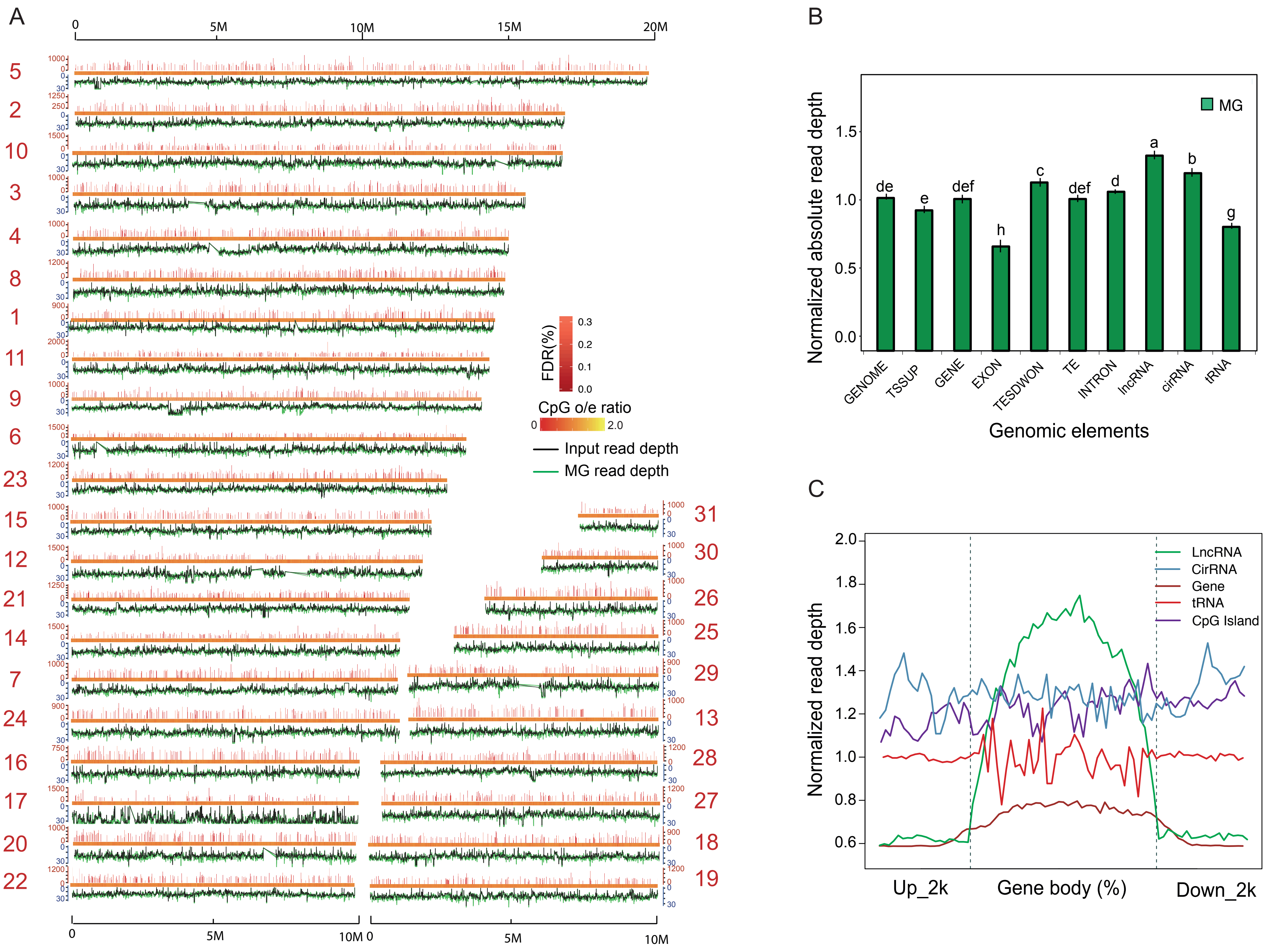
A
B
C
0
5M
10M
15M
20M
5
2
10
3
4
8
1
11
9
6
23
15
12
21
14
7
24
16
17
20
22
31
30
26
25
29
13
28
27
18
19
FDR(%)
0.3
0.2
0.1
0.0
CpG o/e ratio
0
2.0
Input read depth
MG read depth
MG
Normalized absolute read depth
Genomic elements
GENOME
TSSUP
GENE
EXON
TESDWON
TE
INTRON
lncRNA
cirRNA
tRNA
de
e
def
h
c
def
d
a
b
g
LncRNA
CirRNA
Gene
tRNA
CpG Island
Normalized read depth
Up_2k
Gene body (%)
Down_2k

Fig. S3

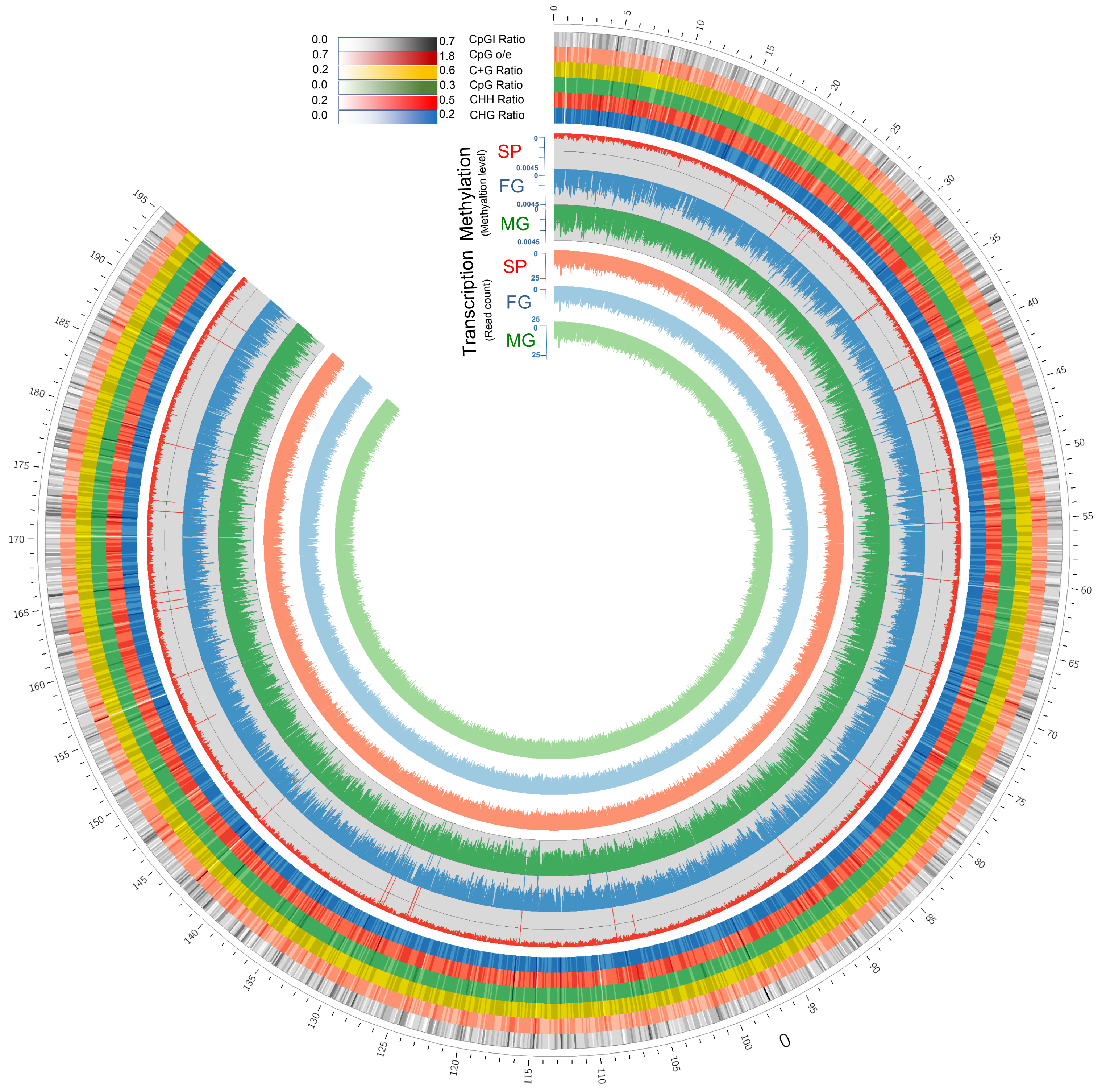

0.0 0.7 CpGI Ratio
0.7 1.8 CpG o/e
0.2 0.6 C+G Ratio
0.0 0.3 CpG Ratio
0.2 0.5 CHH Ratio
0.0 0.2 CHG Ratio
Methylation
(Methyaltion level)
SP
FG
MG
Transcription
(Read count)
SP
FG
MG

Fig. S4

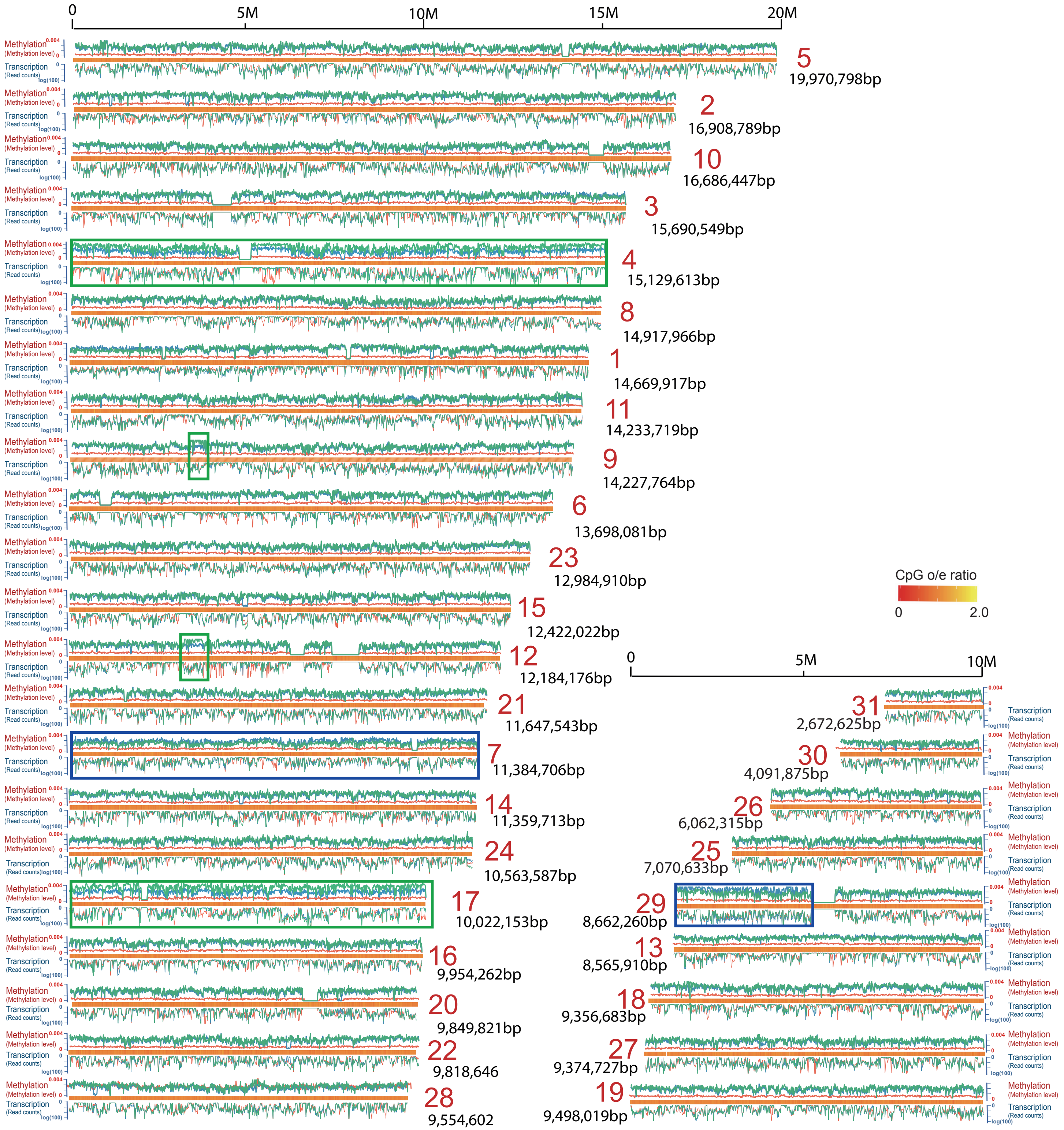
0
5M
10M
15M
20M
Methylation
(Methylation level)
Transcription
(Read counts)
0.004
0
log(100)
5
19,970,798bp
2
16,908,789bp
10
16,686,447bp
3
15,690,549bp
4
15,129,613bp
8
14,917,966bp
1
14,669,917bp
11
14,233,719bp
9
14,227,764bp
6
13,698,081bp
23
12,984,910bp
15
12,422,022bp
12
12,184,176bp
21
11,647,543bp
7
11,384,706bp
14
11,359,713bp
24
10,563,587bp
17
10,022,153bp
16
9,954,262bp
20
9,849,821bp
22
9,818,646
28
9,554,602
CpG o/e ratio
0
2.0
31
2,672,625bp
30
4,091,875bp
26
6,062,315bp
25
7,070,633bp
29
8,662,260bp
13
8,565,910bp
18
9,356,683bp
27
9,374,727bp
19
9,498,019bp

Fig. S5

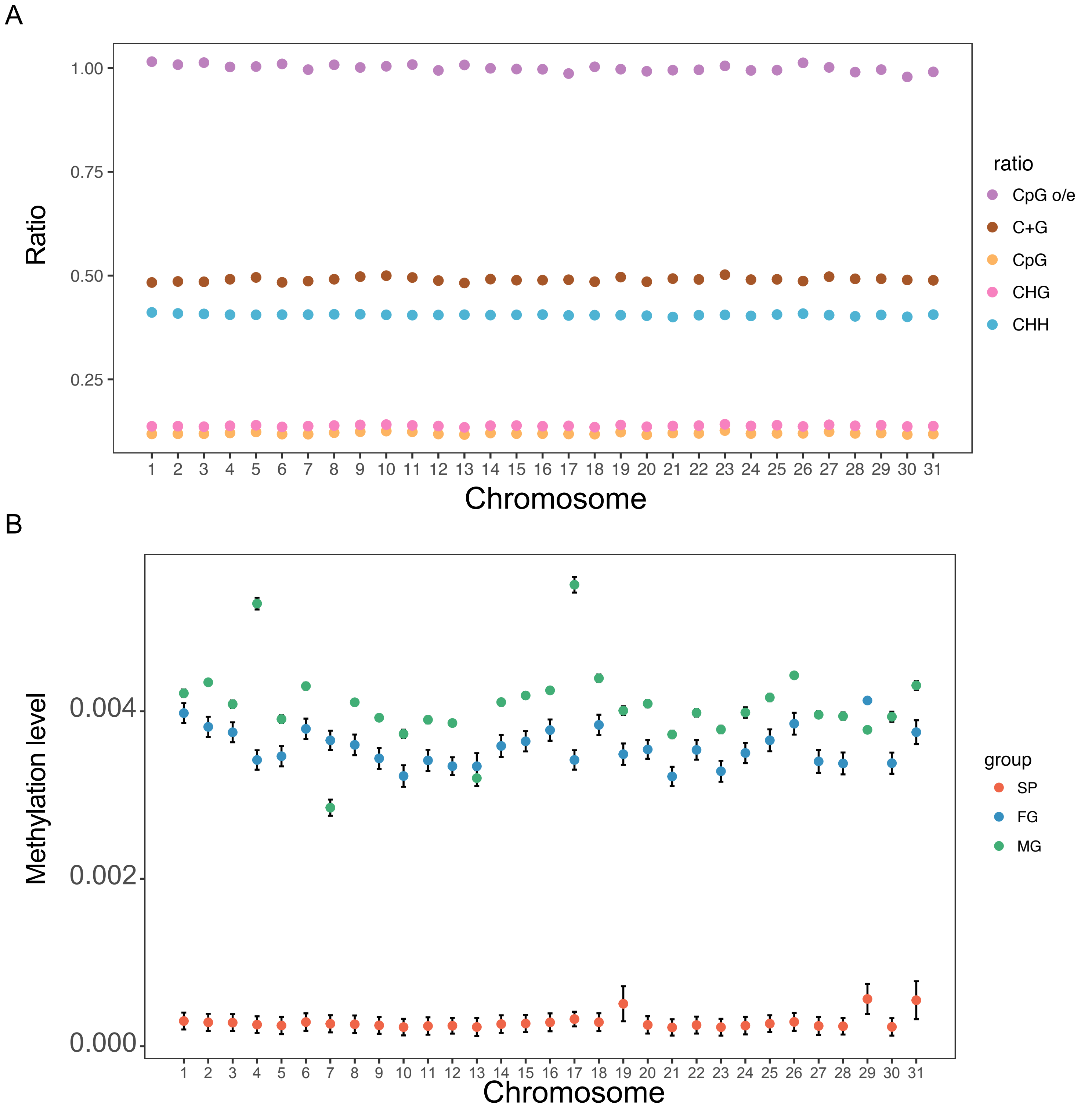
A
Ratio
1.00
0.75
0.50
0.25
Chromosome
1 2 3 4 5 6 7 8 9 10 11 12 13 14 15 16 17 18 19 20 21 22 23 24 25 26 27 28 29 30 31
ratio
CpG o/e
C+G
CpG
CHG
CHH
B
Methylation level
0.004
0.002
0.000
Chromosome
1 2 3 4 5 6 7 8 9 10 11 12 13 14 15 16 17 18 19 20 21 22 23 24 25 26 27 28 29 30 31
group
SP
FG
MG

Fig. S6

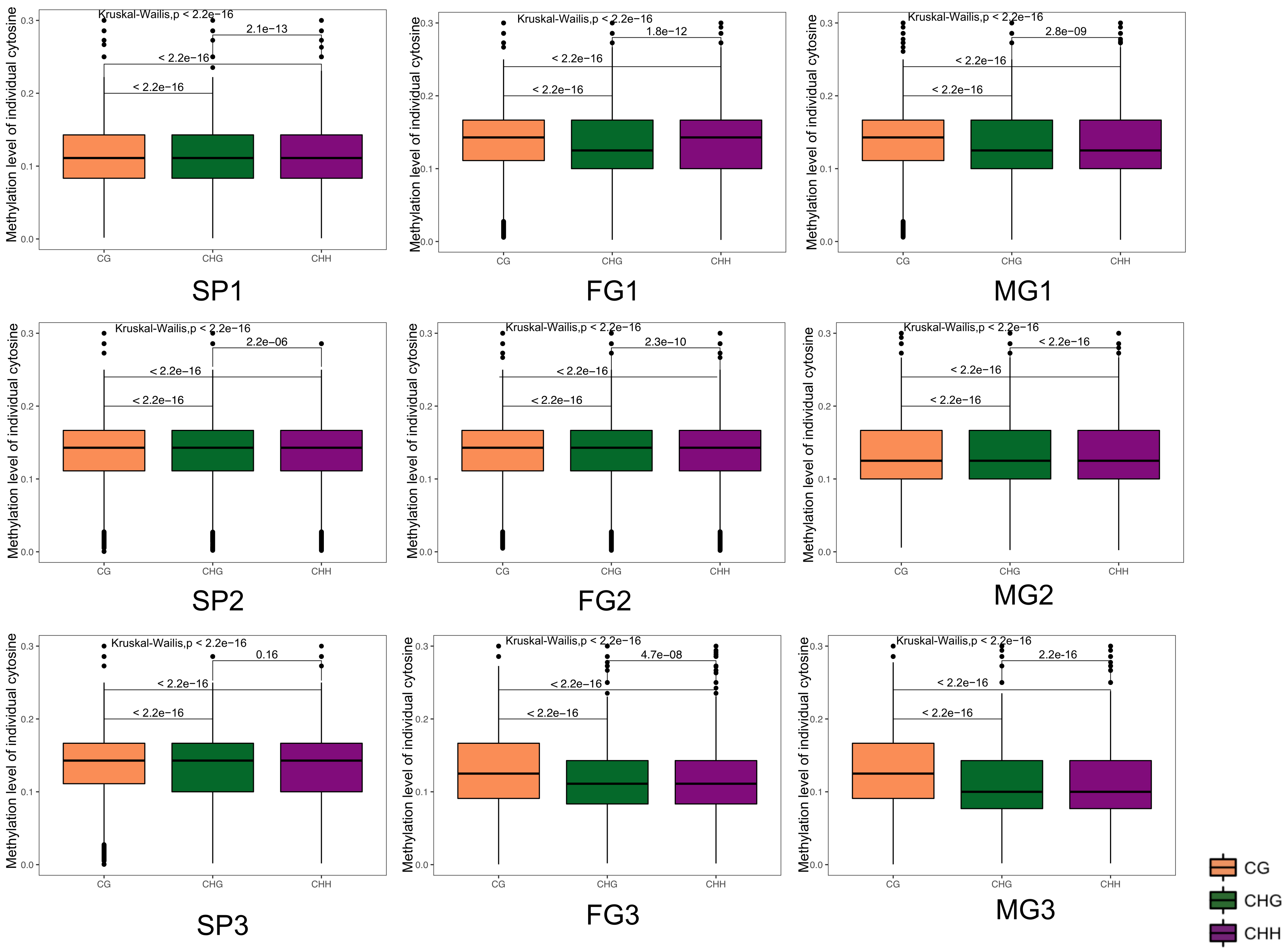
Methylation level of individual cytosine
Kruskal-Wailis,p < 2.2e−16
2.1e−13
< 2.2e−16
CG
CHG
CHH
SP1
1.8e−12
FG1
2.8e−09
MG1
2.2e−06
SP2
2.3e−10
FG2
MG2
0.16
SP3
4.7e−08
FG3
2.2e-16
MG3

Fig. S7

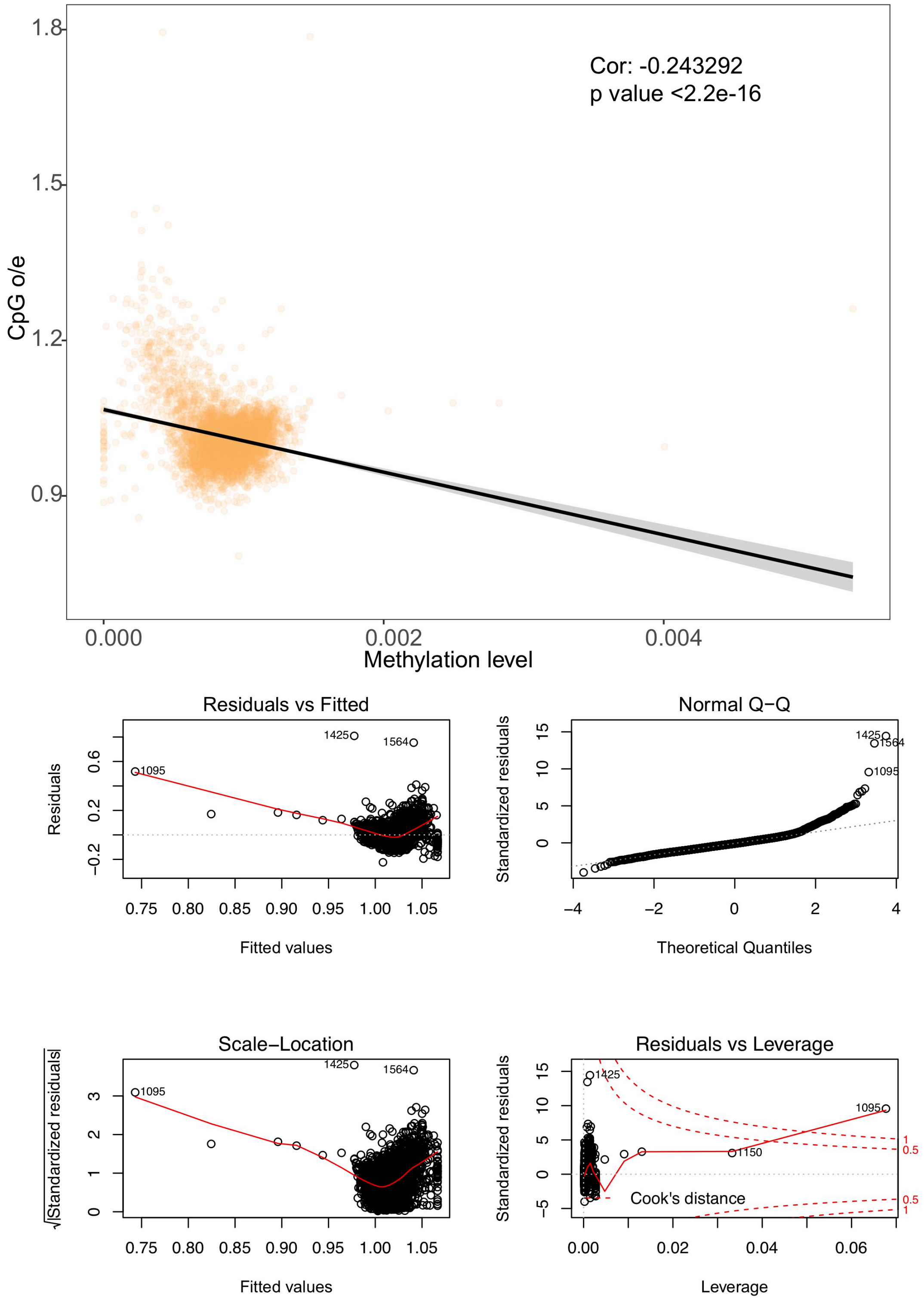
Cor: -0.243292
p value <2.2e-16
CpG o/e
Methylation level
Residuals vs Fitted
Residuals
Fitted values
Normal Q−Q
Standardized residuals
Theoretical Quantiles
Scale−Location
√|Standardized residuals|
Fitted values
Residuals vs Leverage
Standardized residuals
Cook's distance
Leverage

Fig. S8

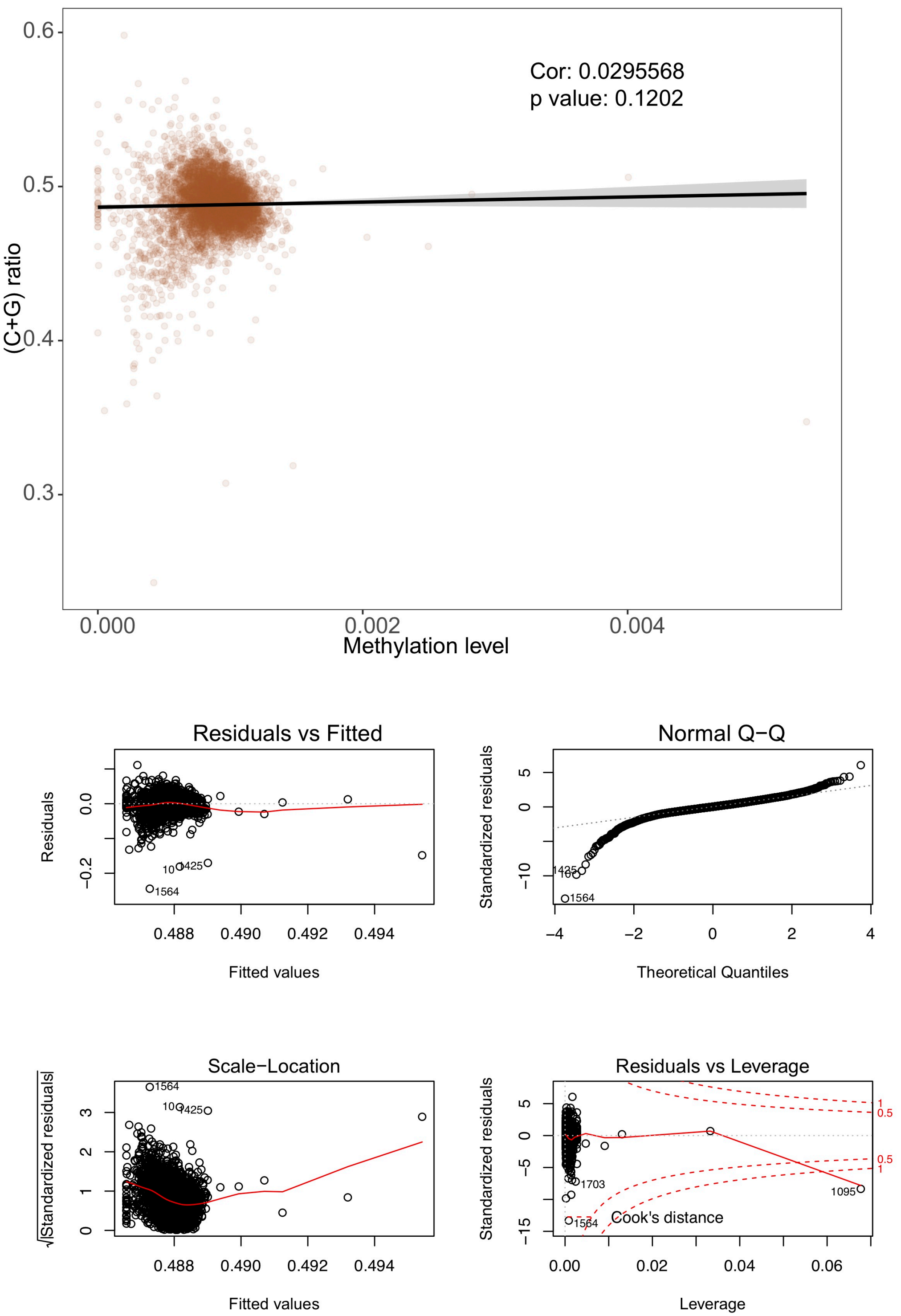
Cor: 0.0295568
p value: 0.1202
(C+G) ratio
Methylation level
0.6
0.5
0.4
0.3
0.000
0.002
0.004
Residuals vs Fitted
Residuals
Fitted values
Normal Q−Q
Standardized residuals
Theoretical Quantiles
Scale−Location
√|Standardized residuals|
Residuals vs Leverage
Cook's distance
Leverage
1564
1425
1703
1095

Fig. S9

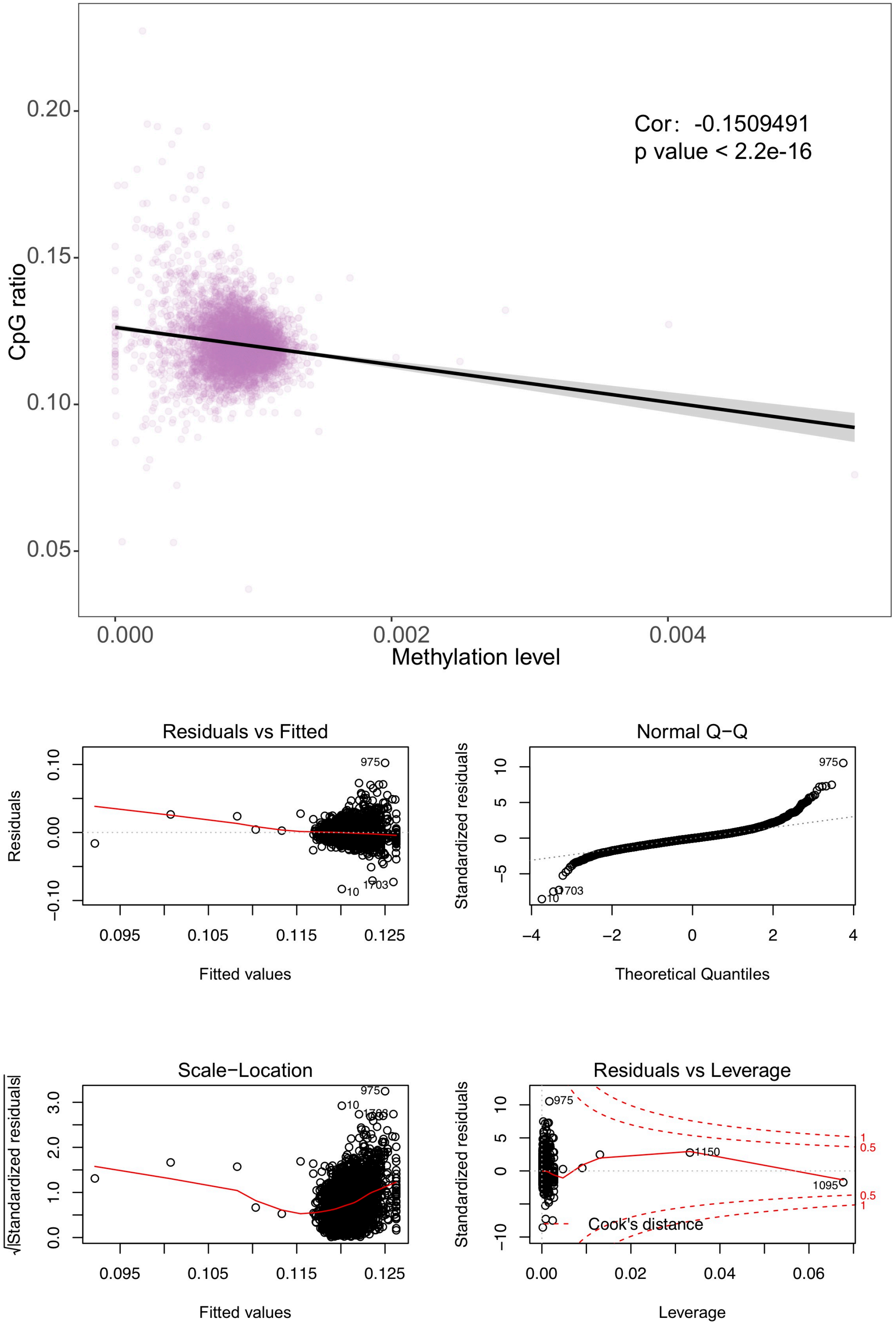
Cor: -0.1509491
p value < 2.2e-16
CpG ratio
Methylation level
Residuals vs Fitted
Residuals
Fitted values
Normal Q−Q
Standardized residuals
Theoretical Quantiles
Scale−Location
√|Standardized residuals|
Fitted values
Residuals vs Leverage
Standardized residuals
Leverage
Cook's distance

Fig. S10

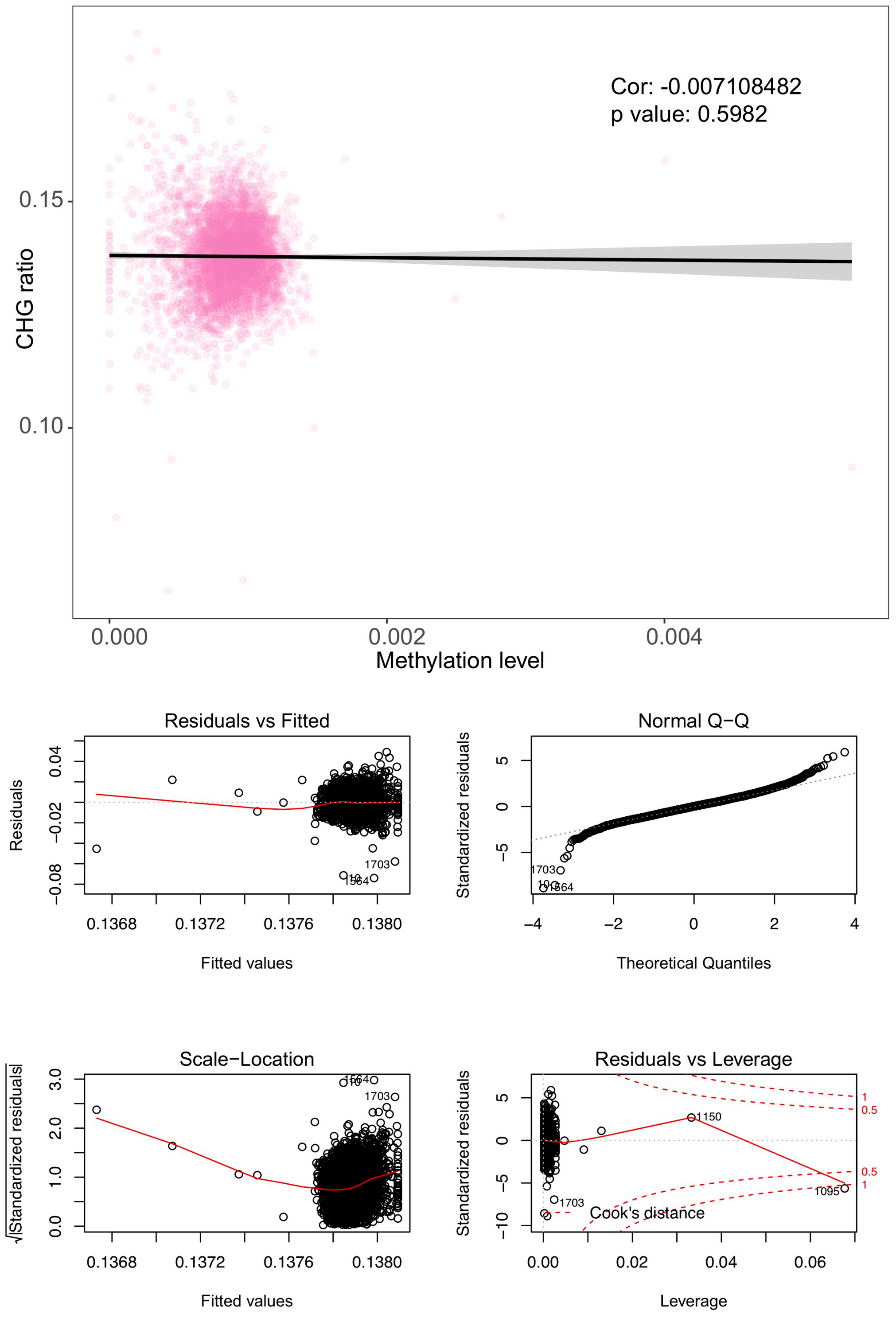
Cor: -0.007108482
p value: 0.5982
CHG ratio
Methylation level
Residuals vs Fitted
Residuals
Fitted values
Normal Q−Q
Standardized residuals
Theoretical Quantiles
Scale−Location
√|Standardized residuals|
Residuals vs Leverage
Cook's distance
Leverage

Fig. S11

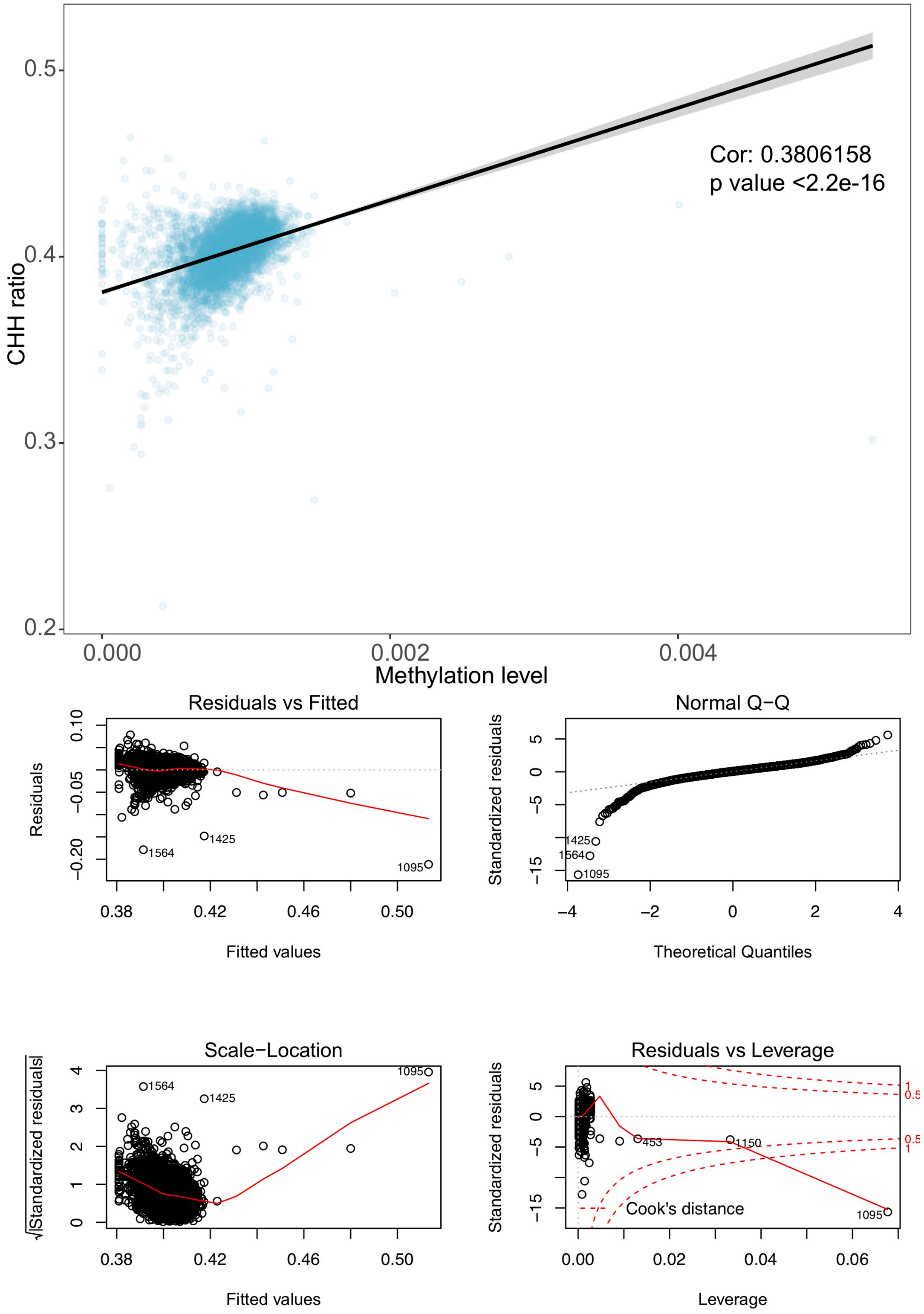

Cor: 0.3806158
p value <2.2e-16
CHH ratio
Methylation level
0.5
0.4
0.3
0.2
0.000
0.002
0.004
Residuals vs Fitted
Residuals
Fitted values
1564
1425
1095
Normal Q−Q
Standardized residuals
Theoretical Quantiles
Scale−Location
√|Standardized residuals|
Residuals vs Leverage
Leverage
453
1150
Cook's distance

Fig. S12

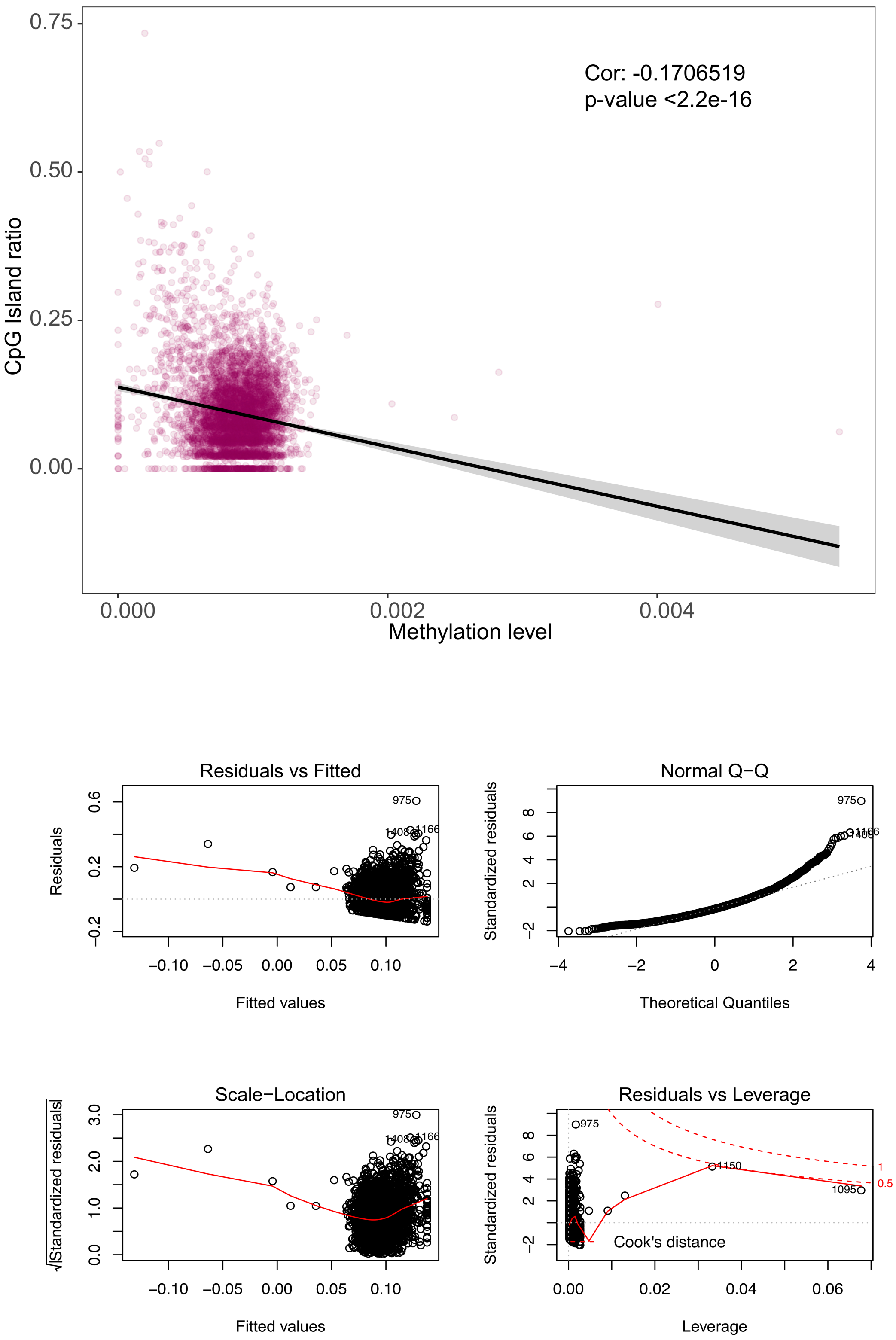
Cor: -0.1706519
p-value <2.2e-16
CpG Island ratio
Methylation level
Residuals vs Fitted
Residuals
Fitted values
Normal Q−Q
Standardized residuals
Theoretical Quantiles
Scale−Location
√|Standardized residuals|
Fitted values
Residuals vs Leverage
Standardized residuals
Cook's distance
Leverage

Fig. S13

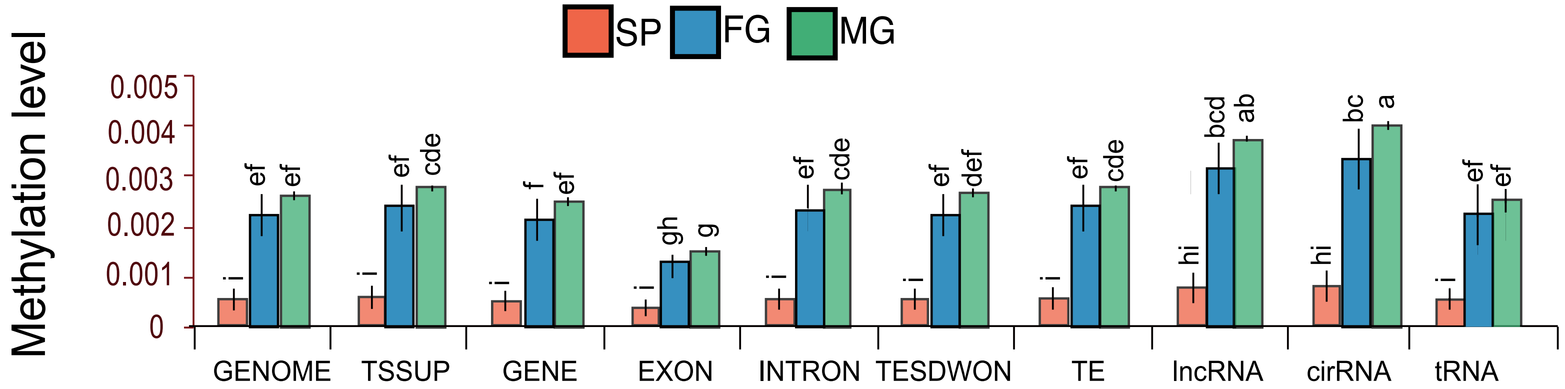
SP
FG
MG
Methylation level
0.005
0.004
0.003
0.002
0.001
0
GENOME
TSSUP
GENE
EXON
INTRON
TESDWON
TE
lncRNA
cirRNA
tRNA
i
ef
ef
i
ef
cde
i
f
ef
i
gh
g
i
ef
cde
i
ef
def
i
ef
cde
hi
bcd
ab
hi
bc
a
i
ef
ef

Fig. S14

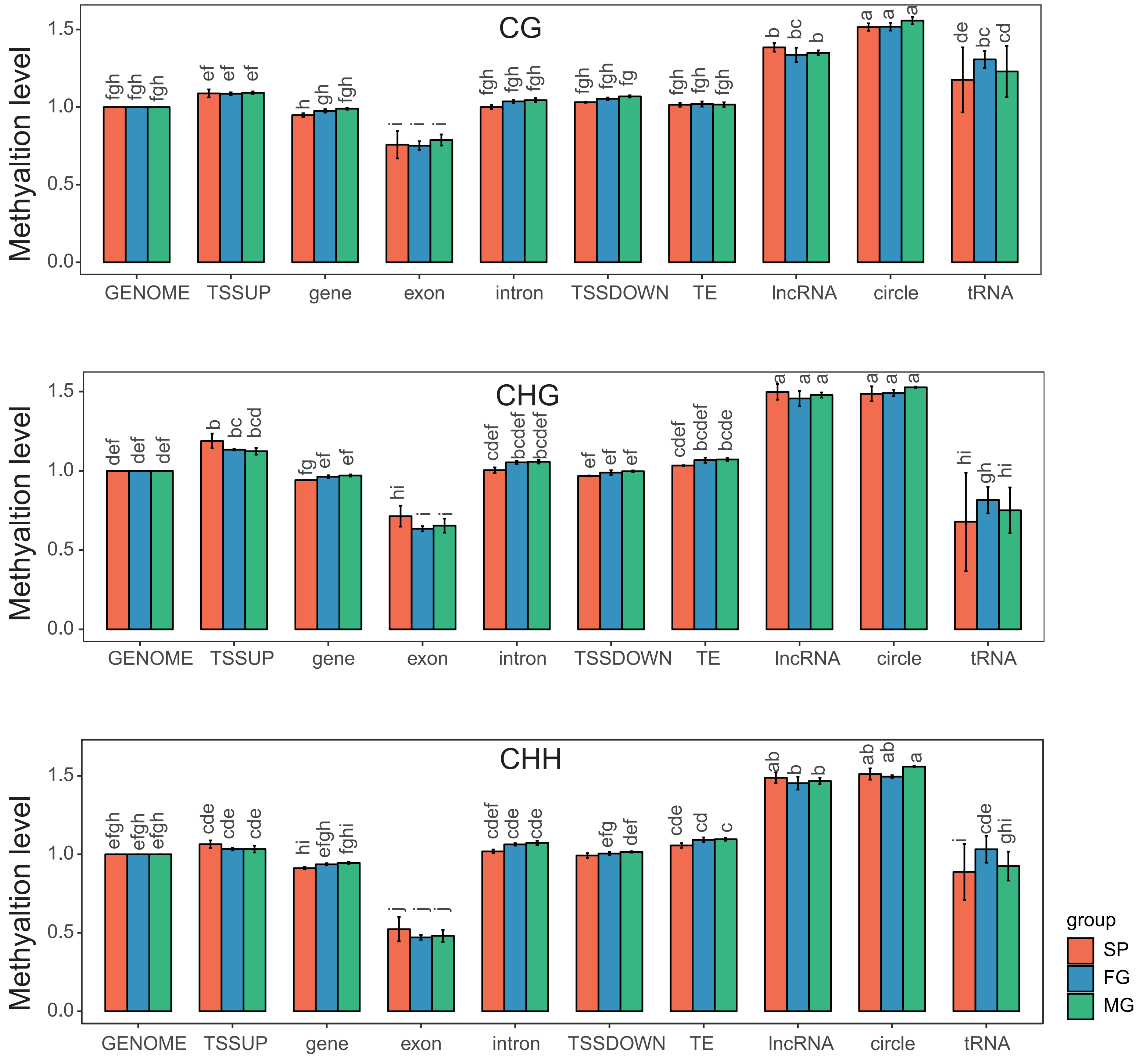
CG
CHG
CHH
Methyaltion level
0.0
0.5
1.0
1.5
GENOME
TSSUP
gene
exon
intron
TSSDOWN
TE
lncRNA
circle
tRNA
group
SP
FG
MG

Fig. S15

A

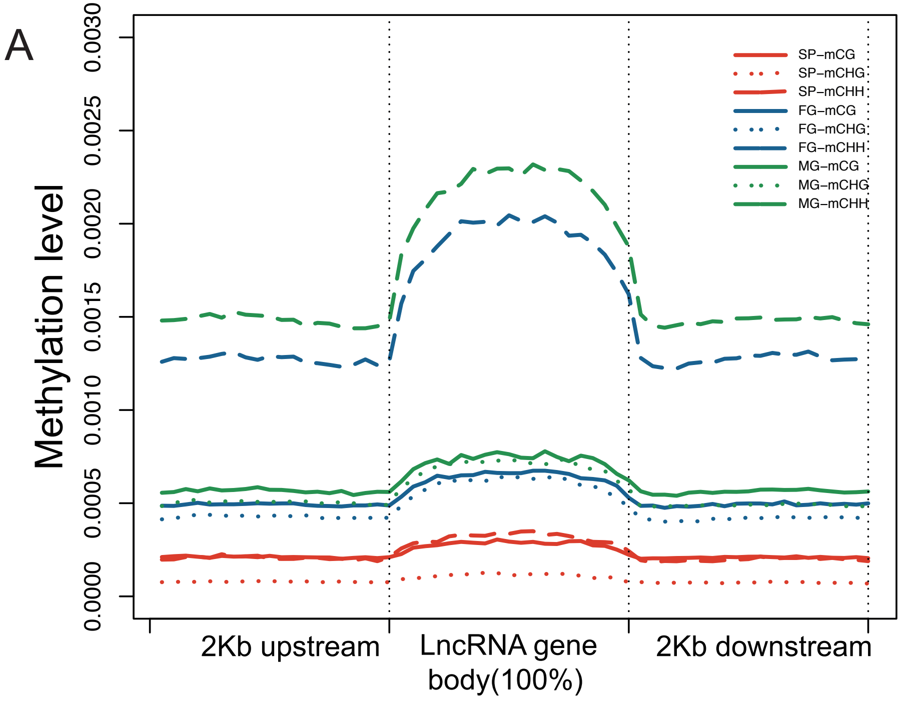

Methylation level
0.0000
0.0005
0.0010
0.0015
0.0020
0.0025
0.0030
SP–mCG
SP–mCHG
SP–mCHH
FG–mCG
FG–mCHG
FG–mCHH
MG–mCG
MG–mCHG
MG–mCHH
2Kb upstream
LncRNA gene body(100%)
2Kb downstream

B

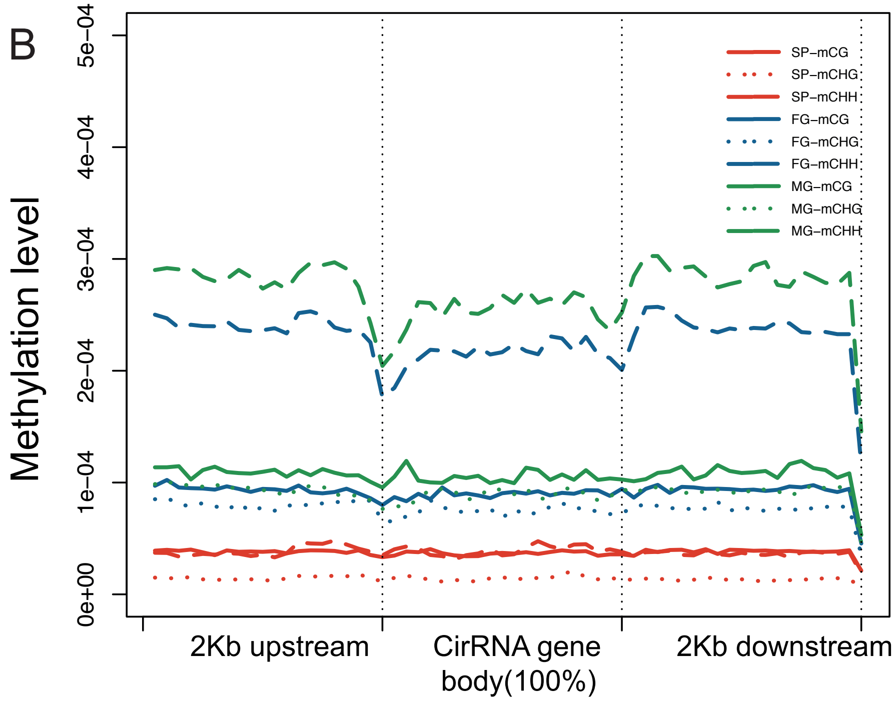

Methylation level
0e+00
1e−04
2e−04
3e−04
4e−04
5e−04
SP–mCG
SP–mCHG
SP–mCHH
FG–mCG
FG–mCHG
FG–mCHH
MG–mCG
MG–mCHG
MG–mCHH
2Kb upstream
CirRNA gene body(100%)
2Kb downstream

Fig. S16

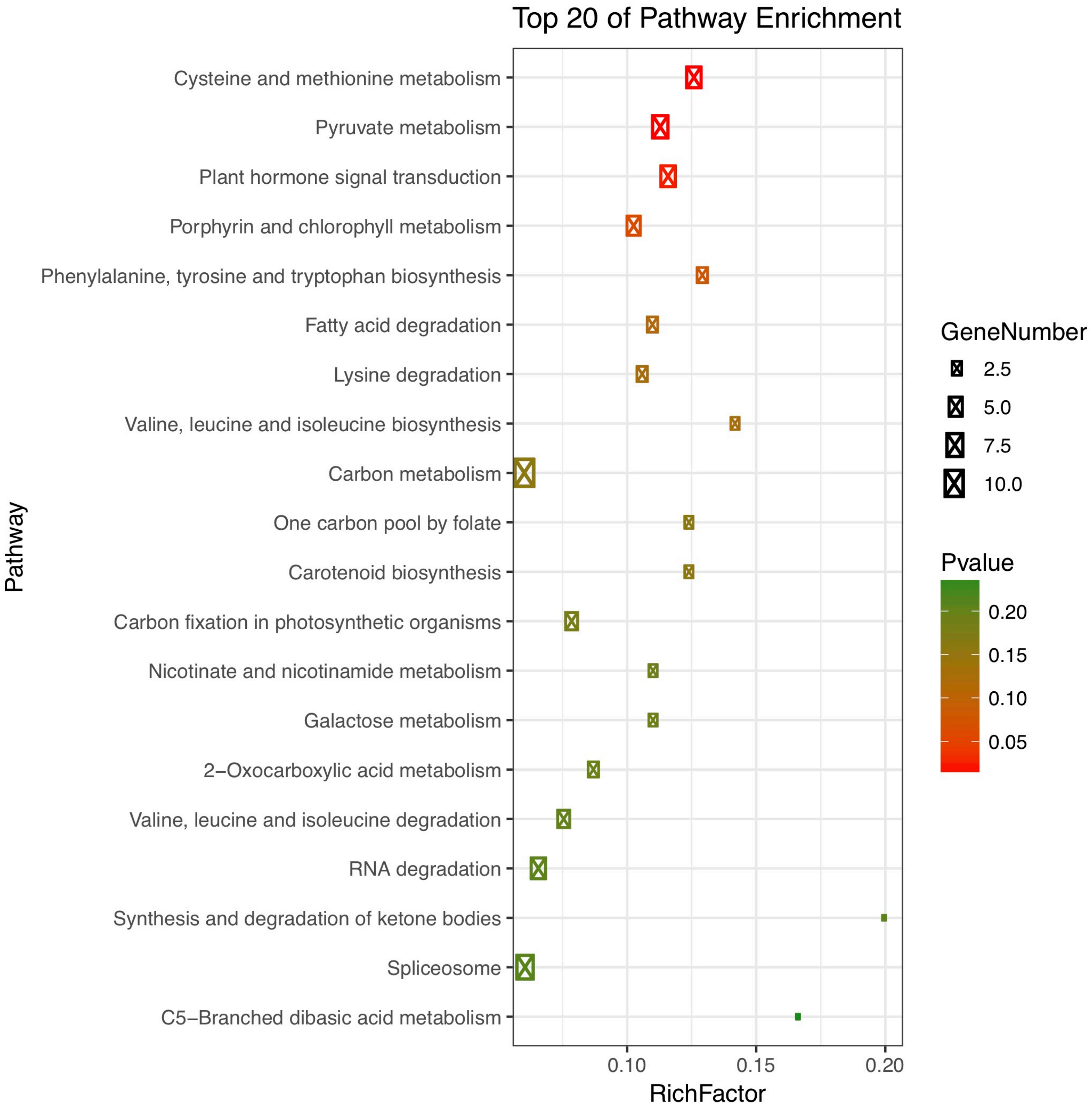
Top 20 of Pathway Enrichment
Cysteine and methionine metabolism
Pyruvate metabolism
Plant hormone signal transduction
Porphyrin and chlorophyll metabolism
Phenylalanine, tyrosine and tryptophan biosynthesis
Fatty acid degradation
Lysine degradation
Valine, leucine and isoleucine biosynthesis
Carbon metabolism
One carbon pool by folate
Carotenoid biosynthesis
Carbon fixation in photosynthetic organisms
Nicotinate and nicotinamide metabolism
Galactose metabolism
2−Oxocarboxylic acid metabolism
Valine, leucine and isoleucine degradation
RNA degradation
Synthesis and degradation of ketone bodies
Spliceosome
C5−Branched dibasic acid metabolism
Pathway
0.10
0.15
0.20
RichFactor
GeneNumber
2.5
5.0
7.5
10.0
Pvalue
0.20
0.15
0.10
0.05

Fig. S17

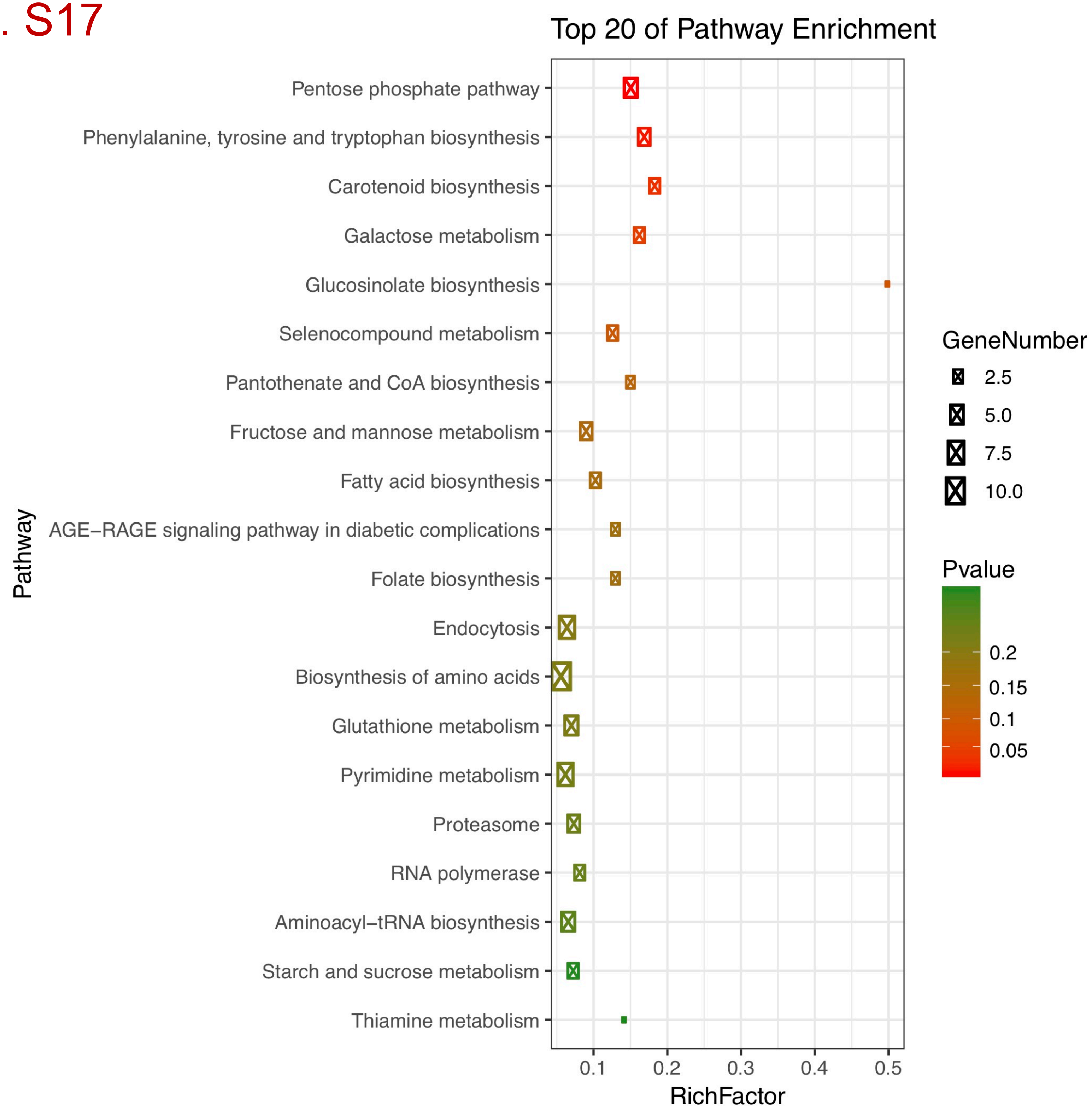

Top 20 of Pathway Enrichment
Pentose phosphate pathway
Phenylalanine, tyrosine and tryptophan biosynthesis
Carotenoid biosynthesis
Galactose metabolism
Glucosinolate biosynthesis
Selenocompound metabolism
Pantothenate and CoA biosynthesis
Fructose and mannose metabolism
Fatty acid biosynthesis
AGE−RAGE signaling pathway in diabetic complications
Folate biosynthesis
Endocytosis
Biosynthesis of amino acids
Glutathione metabolism
Pyrimidine metabolism
Proteasome
RNA polymerase
Aminoacyl−tRNA biosynthesis
Starch and sucrose metabolism
Thiamine metabolism
Pathway
0.1
0.2
0.3
0.4
0.5
RichFactor
GeneNumber
2.5
5.0
7.5
10.0
Pvalue
0.2
0.15
0.1
0.05

Fig. S18

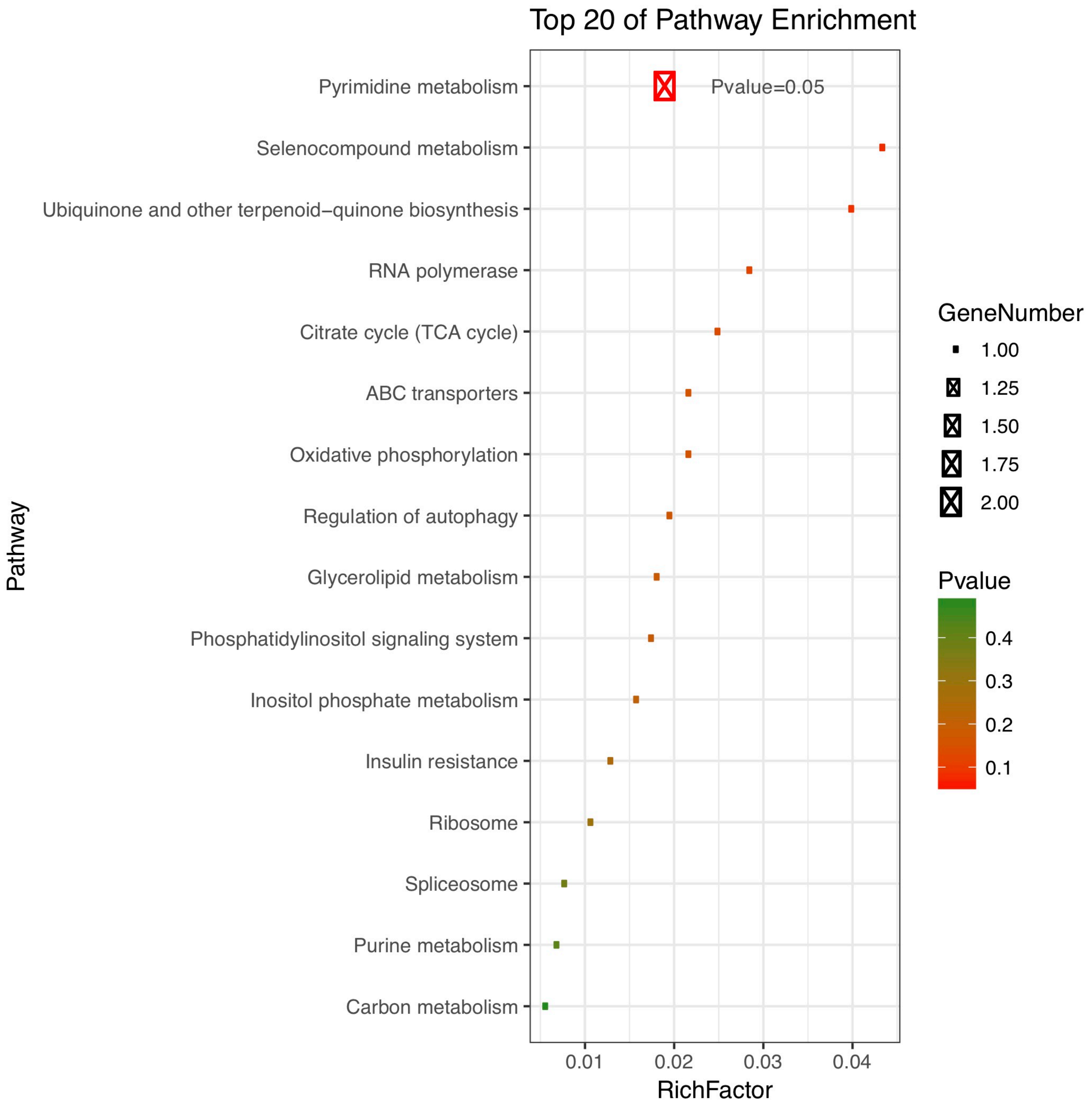

Top 20 of Pathway Enrichment
Pvalue=0.05
Pyrimidine metabolism
Selenocompound metabolism
Ubiquinone and other terpenoid−quinone biosynthesis
RNA polymerase
Citrate cycle (TCA cycle)
ABC transporters
Oxidative phosphorylation
Regulation of autophagy
Glycerolipid metabolism
Phosphatidylinositol signaling system
Inositol phosphate metabolism
Insulin resistance
Ribosome
Spliceosome
Purine metabolism
Carbon metabolism
Pathway
0.01
0.02
0.03
0.04
RichFactor
GeneNumber
1.00
1.25
1.50
1.75
2.00
Pvalue
0.4
0.3
0.2
0.1

Fig. S19

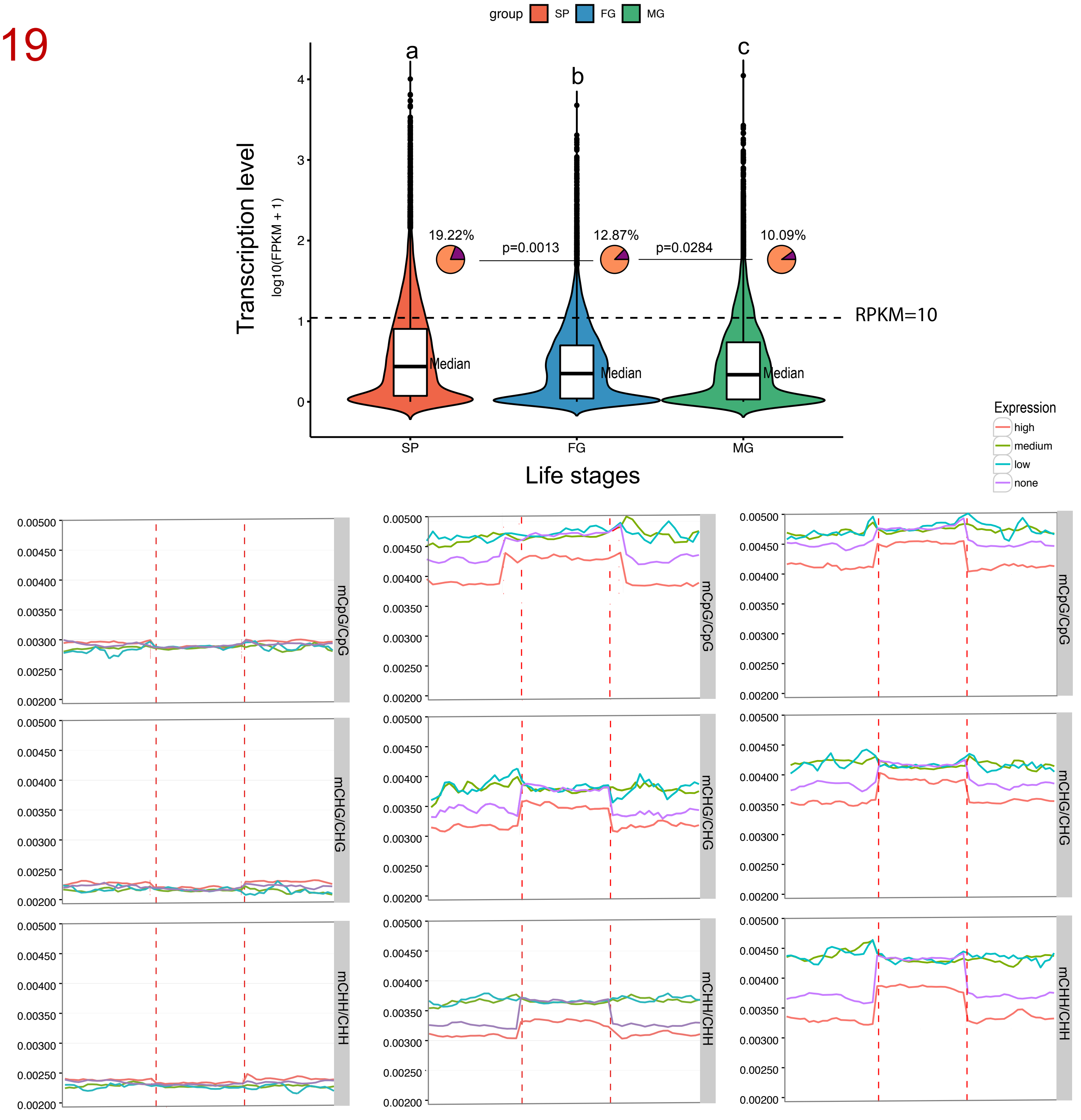
group SP FG MG
a
b
c
Transcription level
log10(FPKM + 1)
19.22%
12.87%
10.09%
p=0.0013
p=0.0284
RPKM=10
Median
SP
FG
MG
Life stages
Expression
high
medium
low
none
mCpG/CpG
mCHG/CHG
mCHH/CHH

Fig. S20

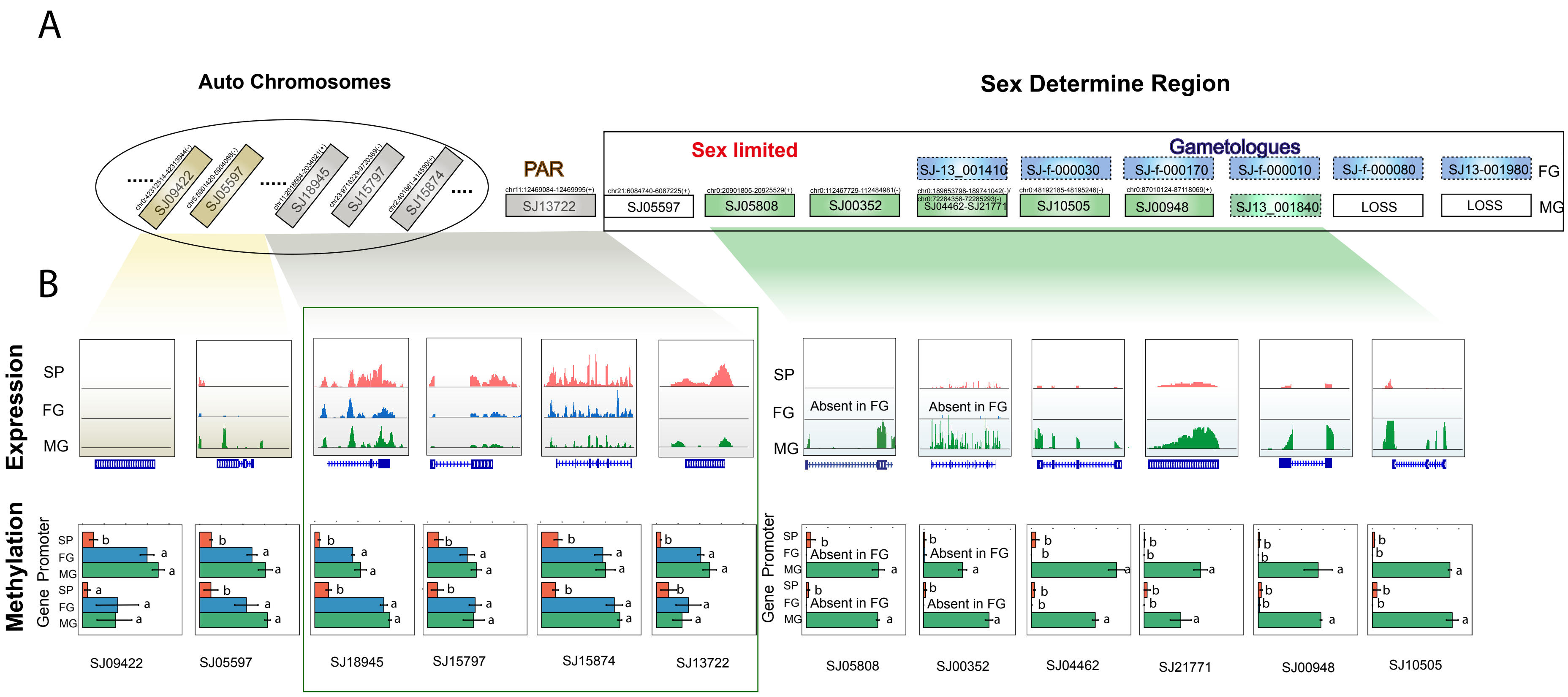
A
Auto Chromosomes
SJ09422
SJ05597
SJ18945
SJ15797
SJ15874
PAR
SJ13722
Sex Determine Region
Sex limited
Gametologues
SJ-13_001410
SJ-f-000030
SJ-f-000170
SJ-f-000010
SJ-f-000080
SJ13-001980
FG
SJ05597
SJ05808
SJ00352
SJ04462-SJ21771
SJ10505
SJ00948
SJ13_001840
LOSS
LOSS
MG
B
Expression
SP
FG
MG
Absent in FG
Methylation
Gene Promoter
SJ09422
SJ05597
SJ18945
SJ15797
SJ15874
SJ13722
SJ05808
SJ00352
SJ04462
SJ21771
SJ00948
SJ10505

Fig. S21

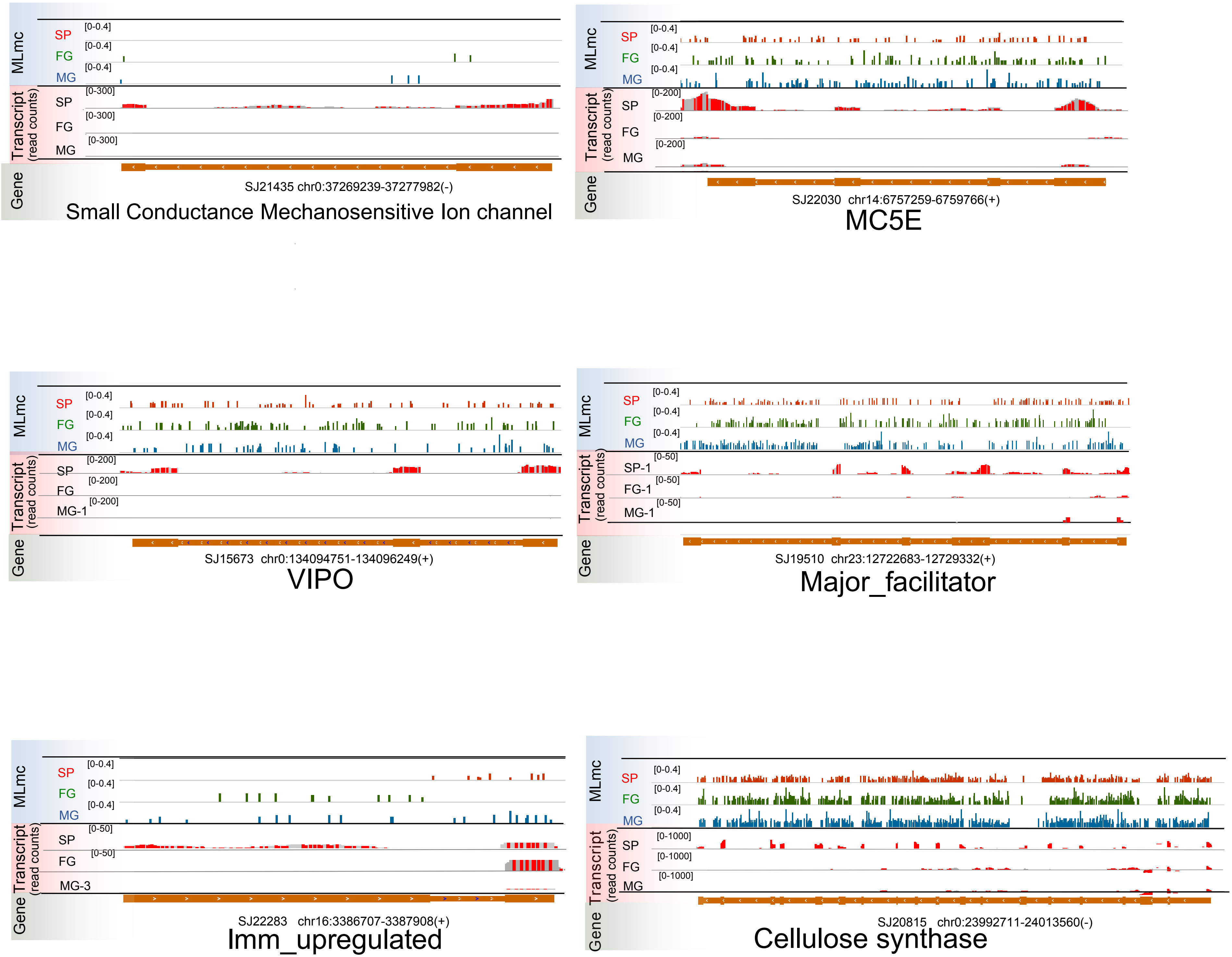
MLmc
SP
FG
MG
[0-0.4]
Transcript (read counts)
[0-300]
Gene
SJ21435 chr0:37269239-37277982(-)
Small Conductance Mechanosensitive Ion channel
[0-200]
SJ22030 chr14:6757259-6759766(+)
MC5E
MG-1
SJ15673 chr0:134094751-134096249(+)
VIPO
SP-1
FG-1
[0-50]
SJ19510 chr23:12722683-12729332(+)
Major_facilitator
MG-3
SJ22283 chr16:3386707-3387908(+)
Imm_upregulated
[0-1000]
SJ20815 chr0:23992711-24013560(-)
Cellulose synthase

Fig. S22

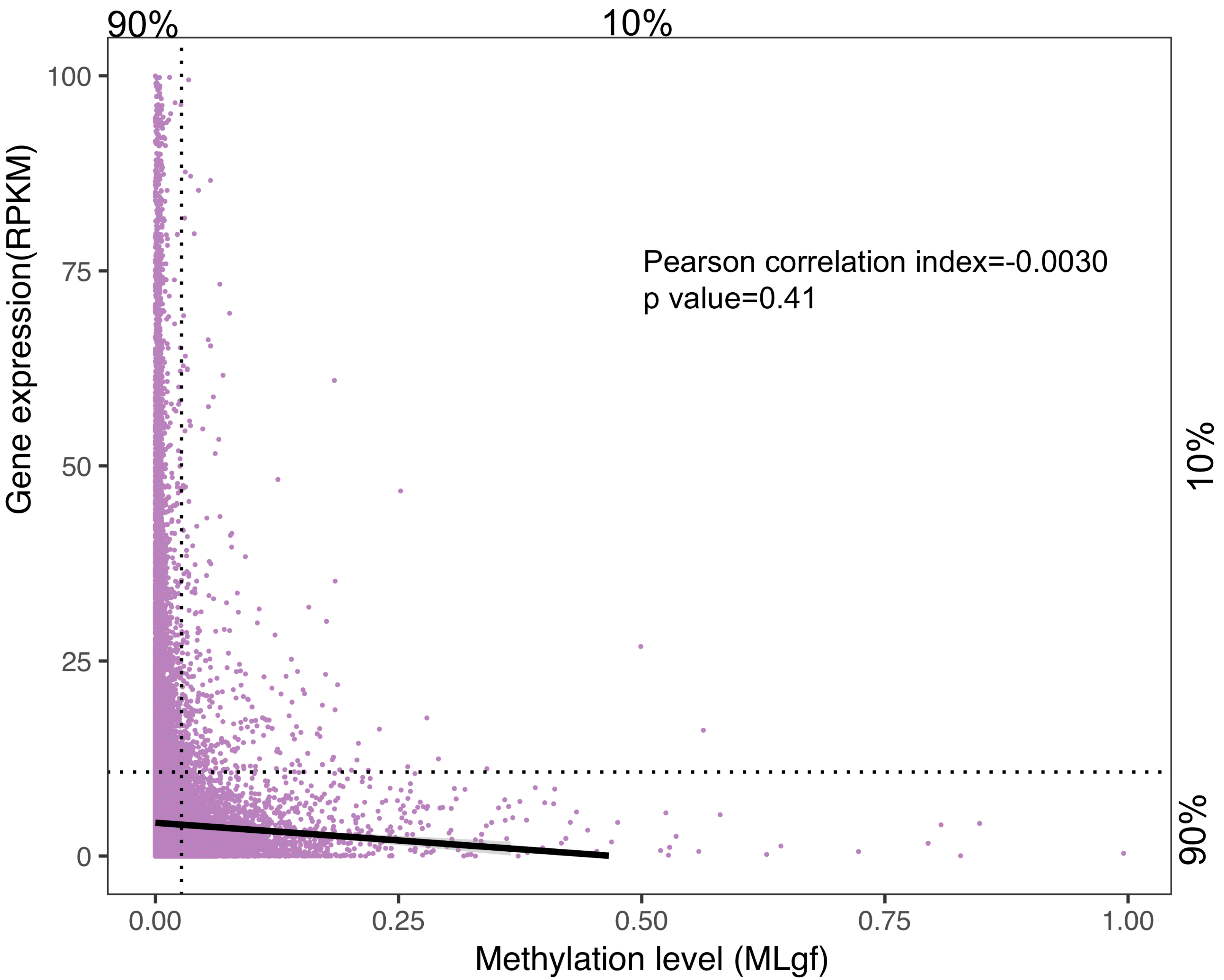

90%
10%
Pearson correlation index=-0.0030
p value=0.41
Gene expression(RPKM)
100
75
50
25
0
10%
90%
0.00
0.25
0.50
0.75
1.00
Methylation level (MLgf)

Fig. S23

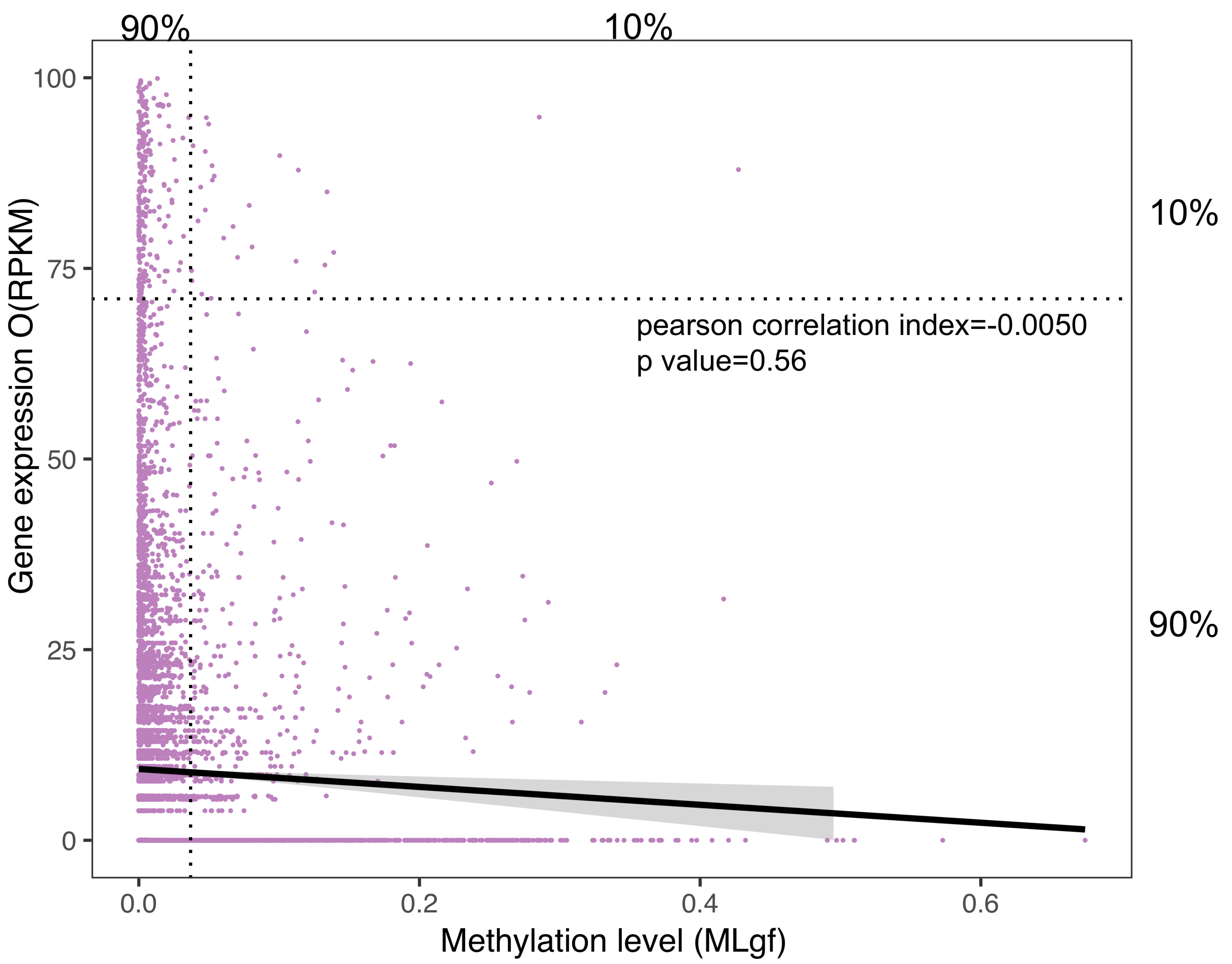
90%
10%
10%
90%
pearson correlation index=-0.0050
p value=0.56
Gene expression O(RPKM)
100
75
50
25
0
0.0
0.2
0.4
0.6
Methylation level (MLgf)

Fig. S24

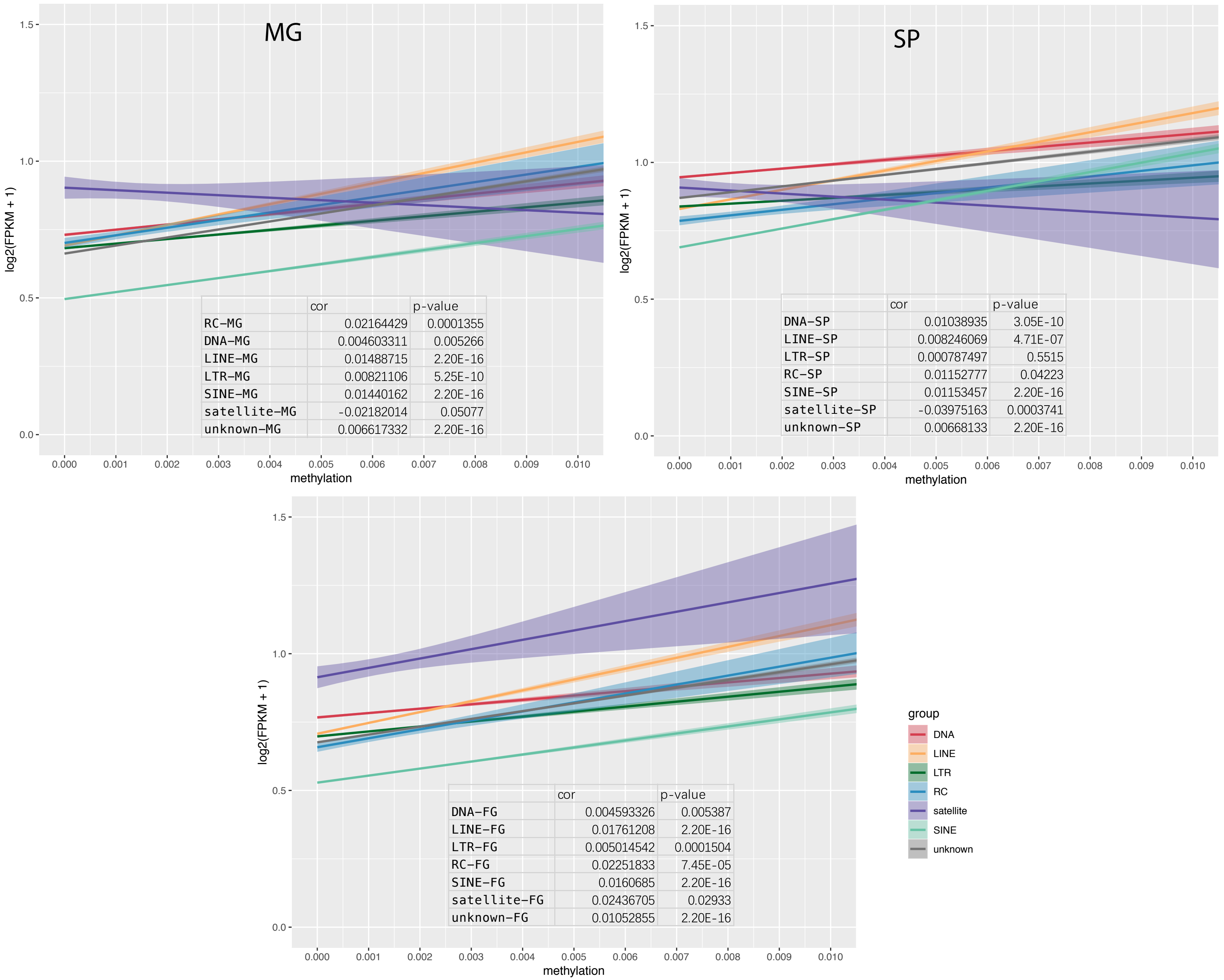
MG
log2(FPKM + 1)
methylation
cor p-value
RC−MG 0.02164429 0.0001355
DNA−MG 0.004603311 0.005266
LINE−MG 0.01488715 2.20E-16
LTR−MG 0.00821106 5.25E-10
SINE−MG 0.01440162 2.20E-16
satellite−MG -0.02182014 0.05077
unknown−MG 0.006617332 2.20E-16
SP
log2(FPKM + 1)
methylation
cor p-value
DNA−SP 0.01038935 3.05E-10
LINE−SP 0.008246069 4.71E-07
LTR−SP 0.000787497 0.5515
RC−SP 0.01152777 0.04223
SINE−SP 0.01153457 2.20E-16
satellite−SP -0.03975163 0.0003741
unknown−SP 0.00668133 2.20E-16
log2(FPKM + 1)
methylation
cor p-value
DNA−FG 0.004593326 0.005387
LINE−FG 0.01761208 2.20E-16
LTR−FG 0.005014542 0.0001504
RC−FG 0.02251833 7.45E-05
SINE−FG 0.0160685 2.20E-16
satellite−FG 0.02436705 0.02933
unknown−FG 0.01052855 2.20E-16
group
DNA
LINE
LTR
RC
satellite
SINE
unknown

Fig. S25

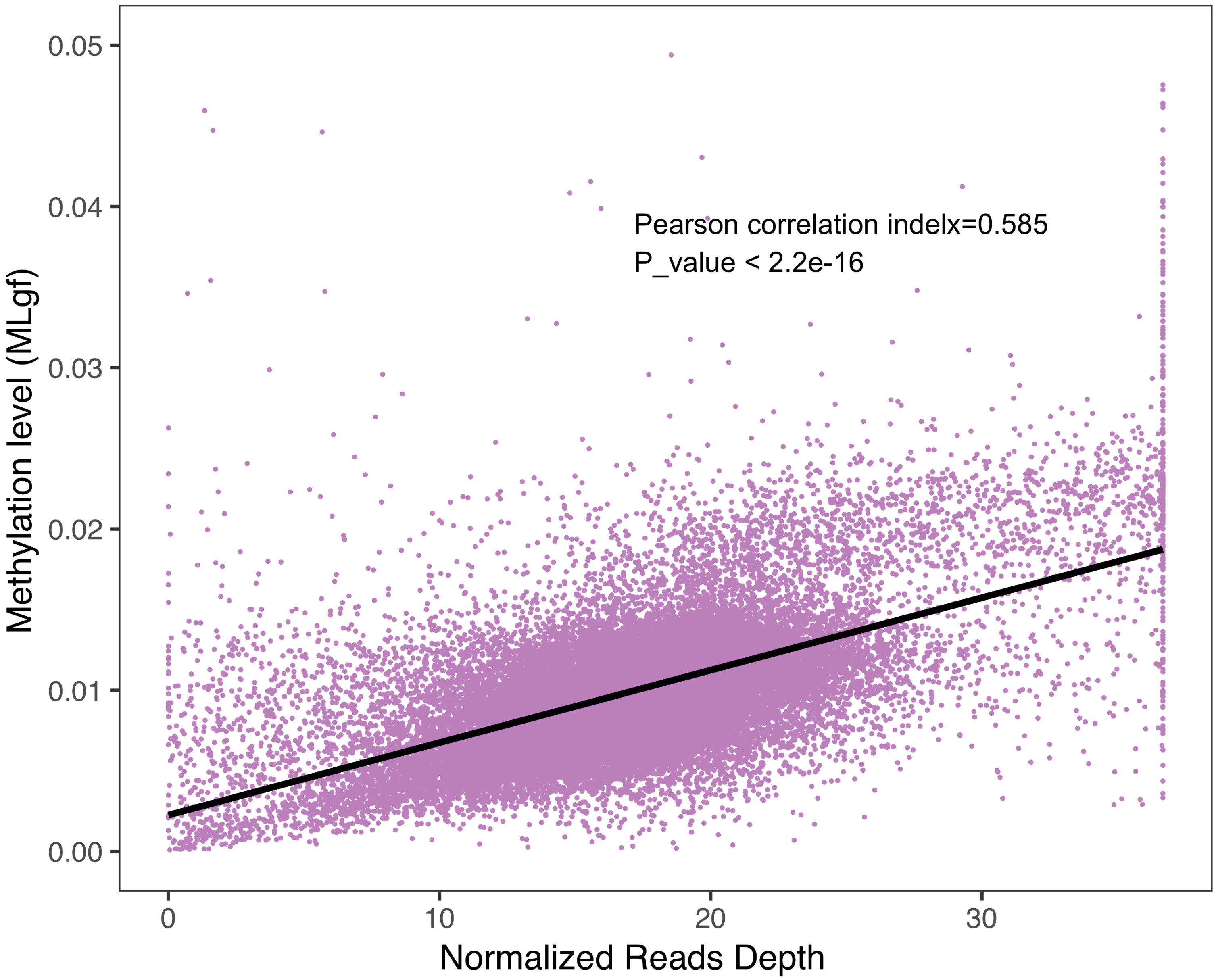
Pearson correlation indelx=0.585
P_value < 2.2e-16
Methylation level (MLgf)
Normalized Reads Depth
0.00
0.01
0.02
0.03
0.04
0.05
0
10
20
30

Fig. S26

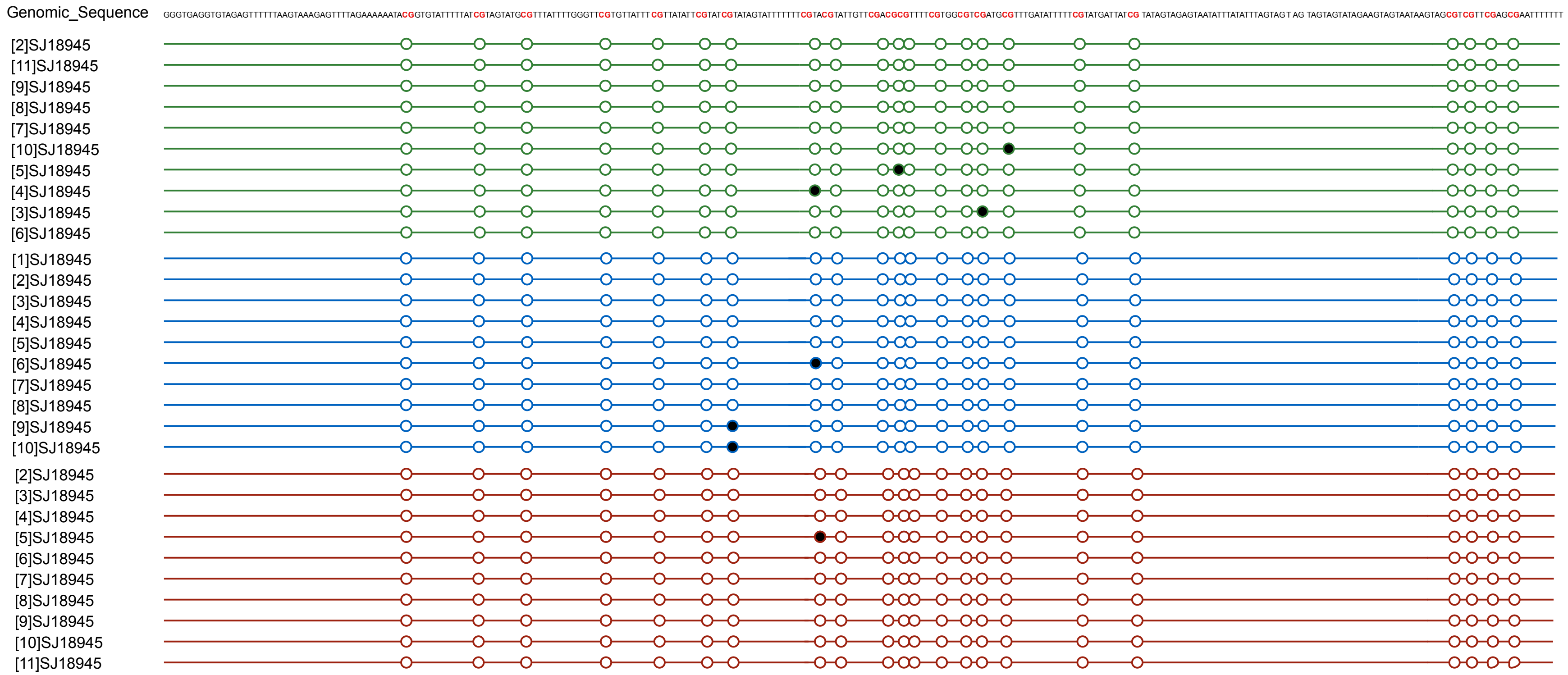
Genomic_Sequence
GGGTGAGGTGTAGAGTTTTTTAAGTAAAGAGTTTTAGAAAAAAATACGGTGTATTTTTATCGTAGTATGCGTTTATTTTGGGTTCGTGTTATTT CGTTATATTCGTATCGTATAGTATTTTTTTCGTACGTATTGTTCGACGCGTTTTCGTGGCGTCGATGCGTTTGATATTTTTCGTATGATTATCG TATAGTAGAGTAATATTTATATTTAGTAGT AG TAGTAGTATAGAAGTAGTAATAAGTAGCGTCGTTCGAGCGAATTTTTTT
[2]SJ18945
[11]SJ18945
[9]SJ18945
[8]SJ18945
[7]SJ18945
[10]SJ18945
[5]SJ18945
[4]SJ18945
[3]SJ18945
[6]SJ18945
[1]SJ18945
[2]SJ18945
[3]SJ18945
[4]SJ18945
[5]SJ18945
[6]SJ18945
[7]SJ18945
[8]SJ18945
[9]SJ18945
[10]SJ18945
[2]SJ18945
[3]SJ18945
[4]SJ18945
[5]SJ18945
[6]SJ18945
[7]SJ18945
[8]SJ18945
[9]SJ18945
[10]SJ18945
[11]SJ18945

Fig. S27

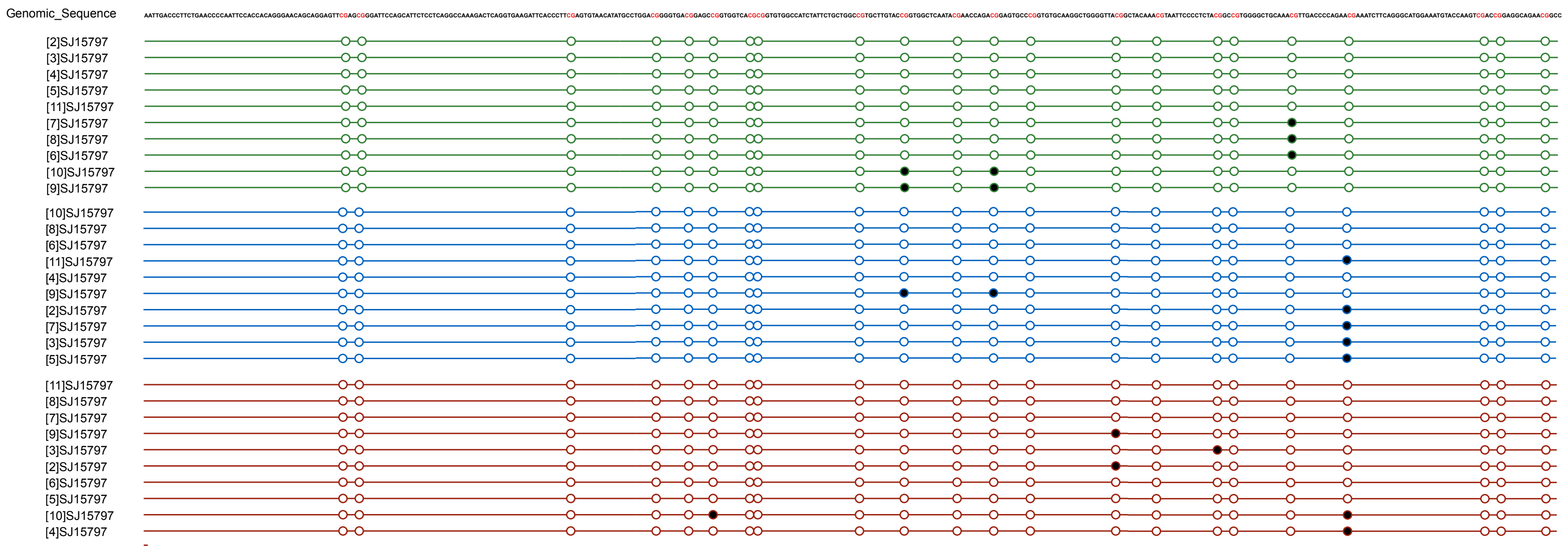
Genomic_Sequence
[2]SJ15797
[3]SJ15797
[4]SJ15797
[5]SJ15797
[11]SJ15797
[7]SJ15797
[8]SJ15797
[6]SJ15797
[10]SJ15797
[9]SJ15797
[10]SJ15797
[8]SJ15797
[6]SJ15797
[11]SJ15797
[4]SJ15797
[9]SJ15797
[2]SJ15797
[7]SJ15797
[3]SJ15797
[5]SJ15797
[11]SJ15797
[8]SJ15797
[7]SJ15797
[9]SJ15797
[3]SJ15797
[2]SJ15797
[6]SJ15797
[5]SJ15797
[10]SJ15797
[4]SJ15797

Fig. S28

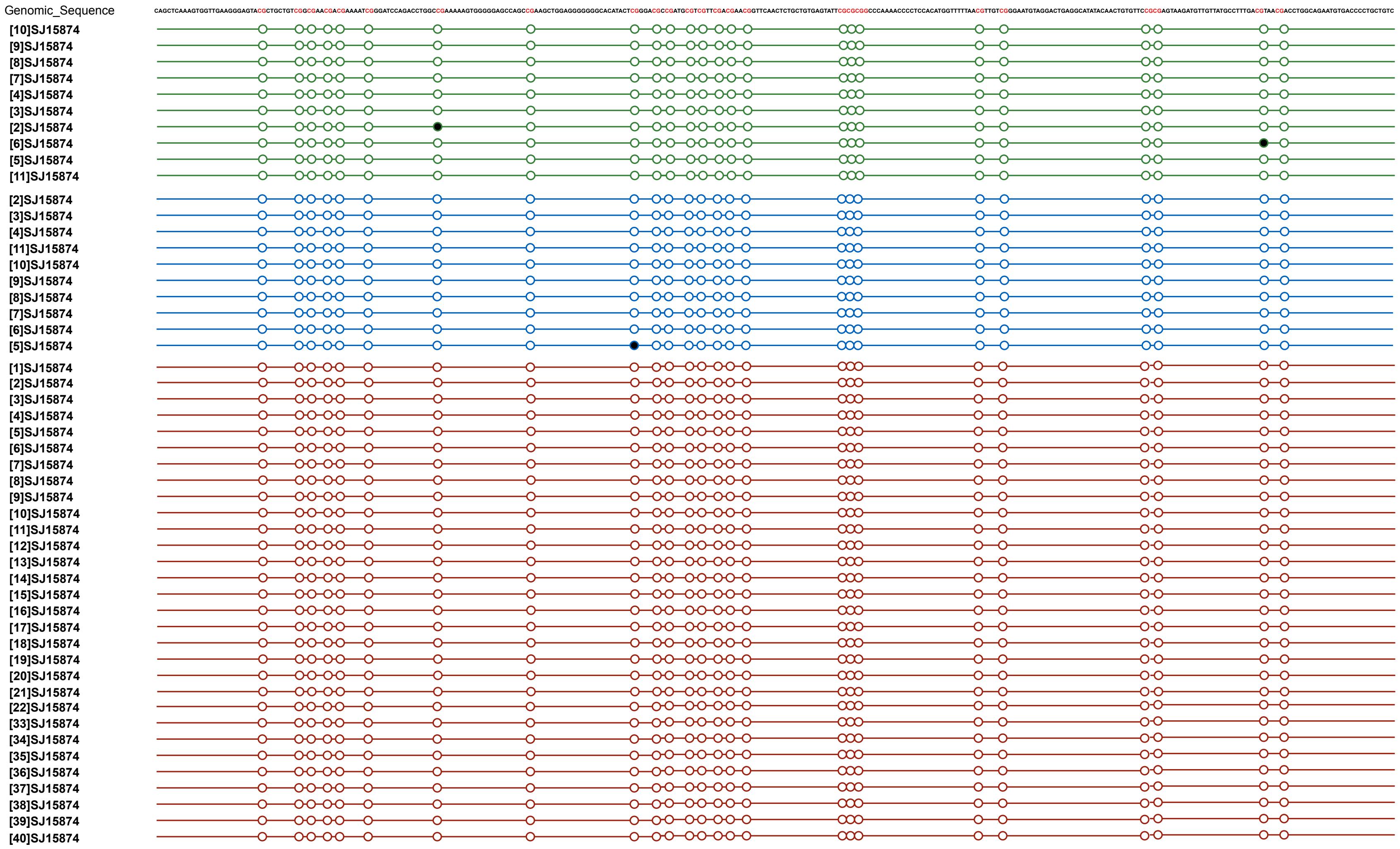
Genomic_Sequence
[10]SJ15874
[9]SJ15874
[8]SJ15874
[7]SJ15874
[4]SJ15874
[3]SJ15874
[2]SJ15874
[6]SJ15874
[5]SJ15874
[11]SJ15874
[2]SJ15874
[3]SJ15874
[4]SJ15874
[11]SJ15874
[10]SJ15874
[9]SJ15874
[8]SJ15874
[7]SJ15874
[6]SJ15874
[5]SJ15874
[1]SJ15874
[2]SJ15874
[3]SJ15874
[4]SJ15874
[5]SJ15874
[6]SJ15874
[7]SJ15874
[8]SJ15874
[9]SJ15874
[10]SJ15874
[11]SJ15874
[12]SJ15874
[13]SJ15874
[14]SJ15874
[15]SJ15874
[16]SJ15874
[17]SJ15874
[18]SJ15874
[19]SJ15874
[20]SJ15874
[21]SJ15874
[22]SJ15874
[33]SJ15874
[34]SJ15874
[35]SJ15874
[36]SJ15874
[37]SJ15874
[38]SJ15874
[39]SJ15874
[40]SJ15874

Fig. S29

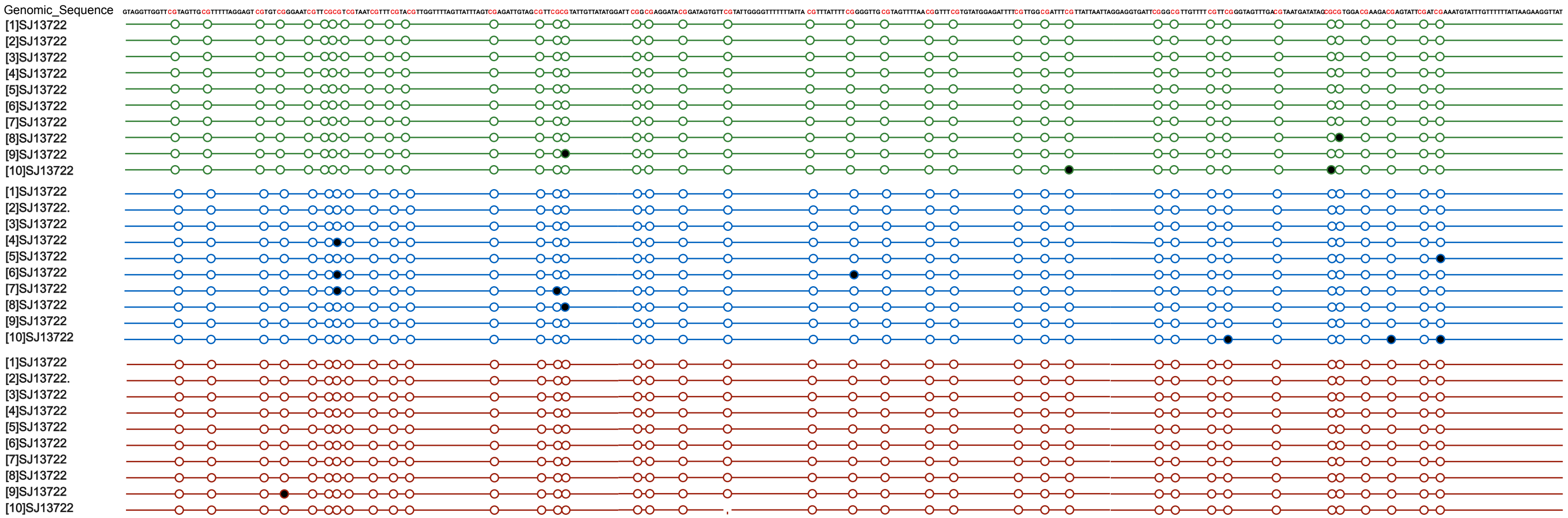
Genomic_Sequence
[1]SJ13722
[2]SJ13722
[3]SJ13722
[4]SJ13722
[5]SJ13722
[6]SJ13722
[7]SJ13722
[8]SJ13722
[9]SJ13722
[10]SJ13722
[1]SJ13722
[2]SJ13722.
[3]SJ13722
[4]SJ13722
[5]SJ13722
[6]SJ13722
[7]SJ13722
[8]SJ13722
[9]SJ13722
[10]SJ13722
[1]SJ13722
[2]SJ13722.
[3]SJ13722
[4]SJ13722
[5]SJ13722
[6]SJ13722
[7]SJ13722
[8]SJ13722
[9]SJ13722
[10]SJ13722

Fig. S30

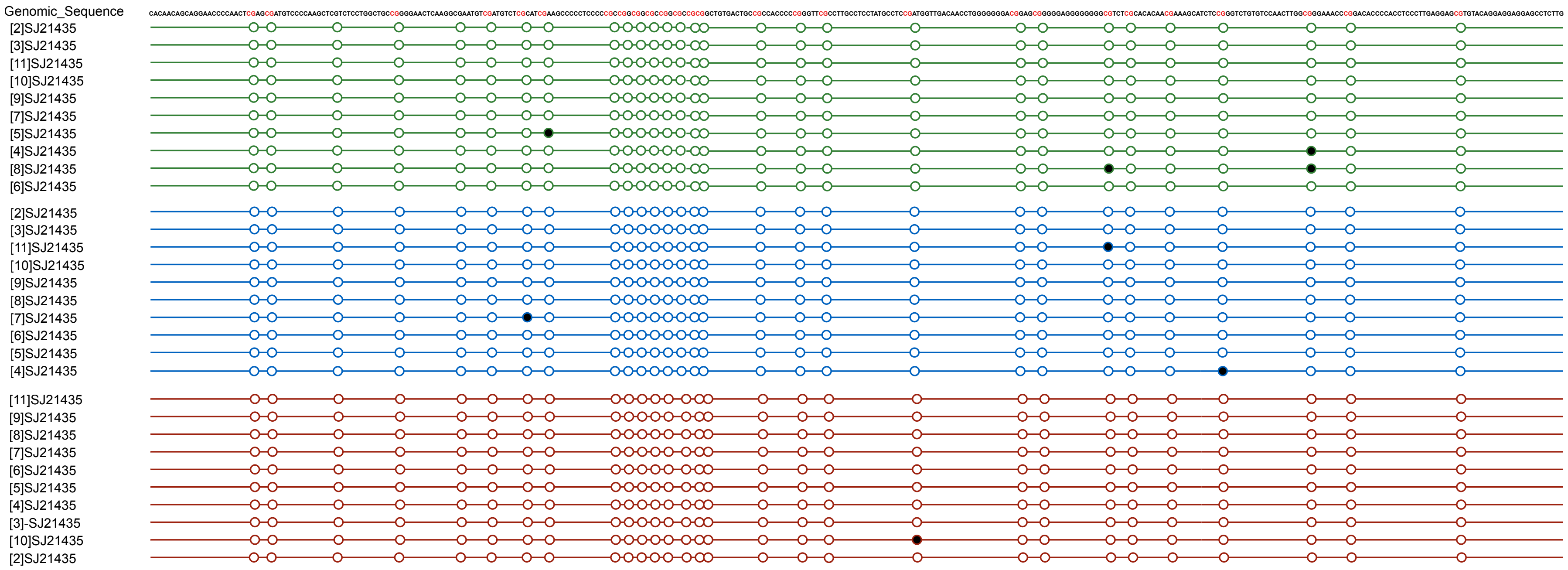

Genomic_Sequence
[2]SJ21435
[3]SJ21435
[11]SJ21435
[10]SJ21435
[9]SJ21435
[7]SJ21435
[5]SJ21435
[4]SJ21435
[8]SJ21435
[6]SJ21435
[2]SJ21435
[3]SJ21435
[11]SJ21435
[10]SJ21435
[9]SJ21435
[8]SJ21435
[7]SJ21435
[6]SJ21435
[5]SJ21435
[4]SJ21435
[11]SJ21435
[9]SJ21435
[8]SJ21435
[7]SJ21435
[6]SJ21435
[5]SJ21435
[4]SJ21435
[3]-SJ21435
[10]SJ21435
[2]SJ21435

Fig. S31

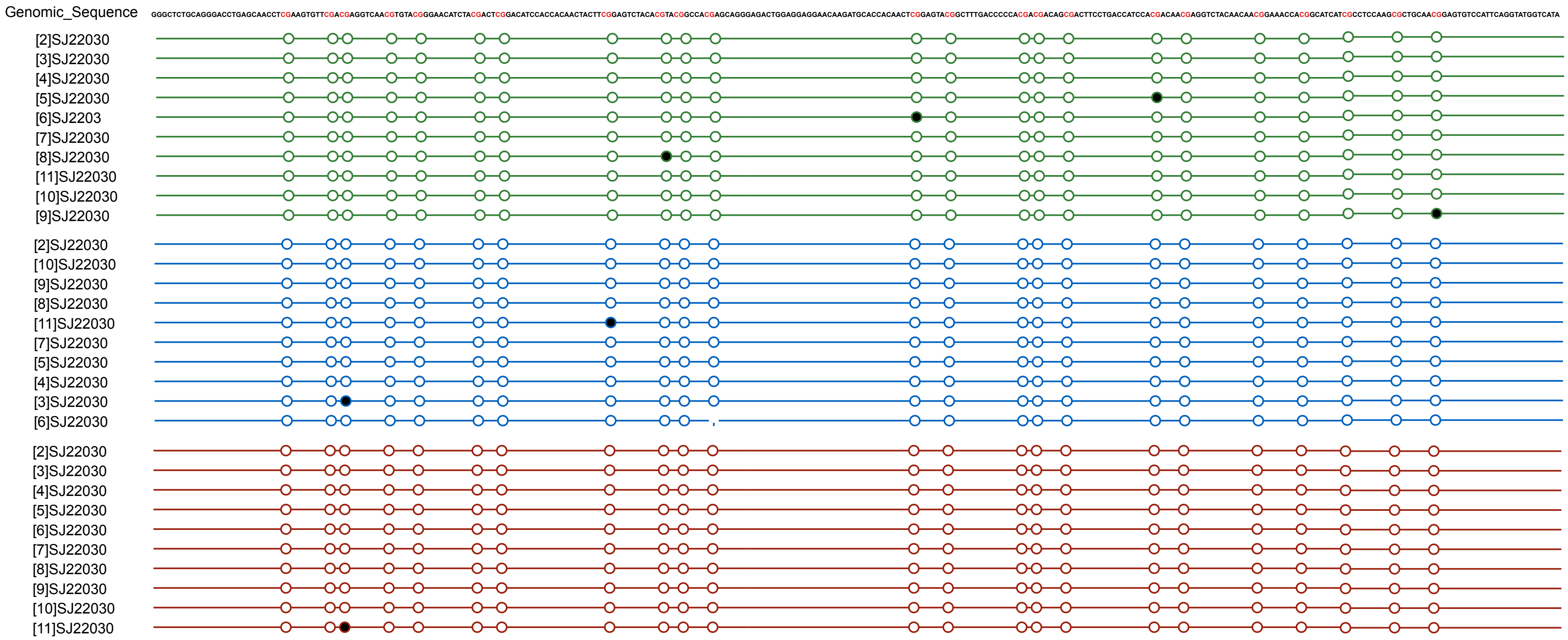
Genomic_Sequence
GGGCTCTGCAGGGACCTGAGCAACCTCGAAGTGTTCGACGAGGTCAACGTGTACGGGAACATCTACGACTCGGACATCCACCACAACTACTTCGGAGTCTACACGTACGGCCACGAGCAGGGAGACTGGAGGAGGAACAAGATGCACCACAACTCGGAGTACGGCTTTGACCCCCACGACGACAGCGACTTCCTGACCATCCACGACAACCGAGGTCTACAACAACGGAAACCACGGCATCATCGCCTCCAAGCGCTGCAACGGAGTGTCCATTCAGGTATGGTCATA
[2]SJ22030
[3]SJ22030
[4]SJ22030
[5]SJ22030
[6]SJ2203
[7]SJ22030
[8]SJ22030
[11]SJ22030
[10]SJ22030
[9]SJ22030
[2]SJ22030
[10]SJ22030
[9]SJ22030
[8]SJ22030
[11]SJ22030
[7]SJ22030
[5]SJ22030
[4]SJ22030
[3]SJ22030
[6]SJ22030
[2]SJ22030
[3]SJ22030
[4]SJ22030
[5]SJ22030
[6]SJ22030
[7]SJ22030
[8]SJ22030
[9]SJ22030
[10]SJ22030
[11]SJ22030

Fig. S32

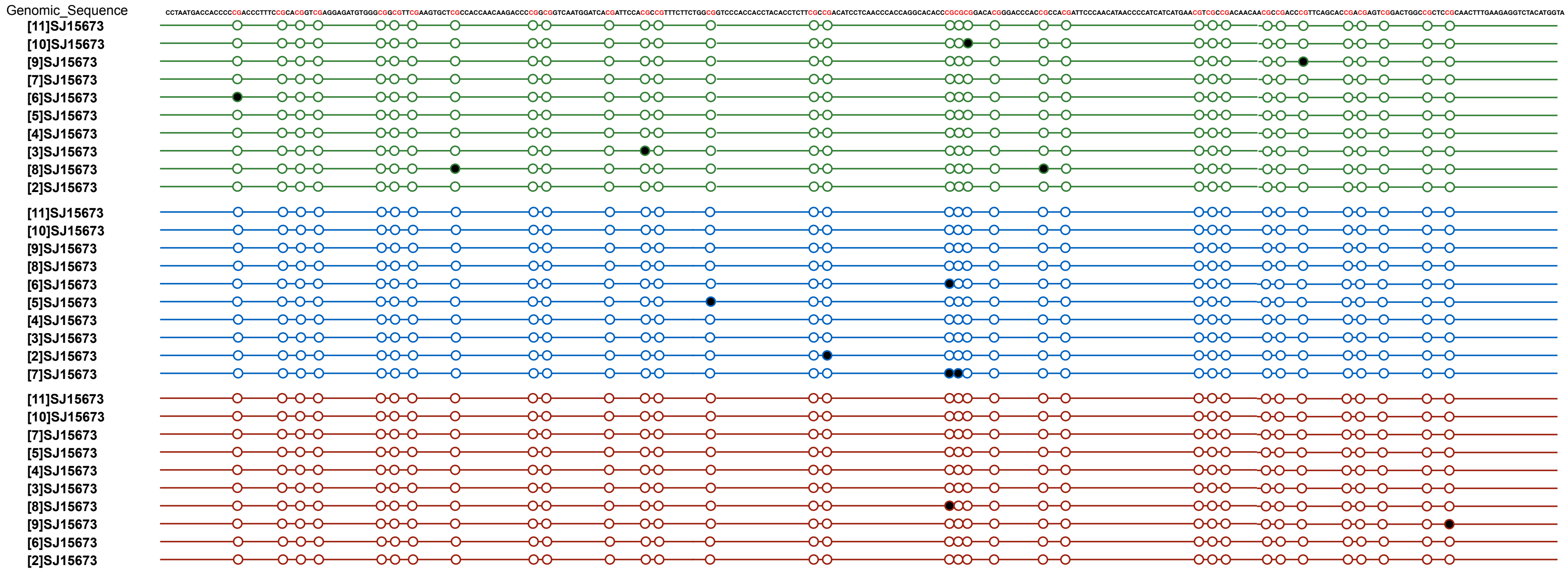
Genomic_Sequence
CCTAATGACCACCCCCGACCCTTTCCGCACGGTCGAGGAGATGTGGGCGGCGTTCGAAGTGCTCGCCACCAACAAGACCCCGGCGGTCAATGGATCACGATTCCACGCCGTTTCTTCTGGCGGTCCCACCACCTACACCTCTTCGCCGACATCCTCAACCCACCAGGCACACCCGCGCGGACACGGGACCCACCGCCACGATTCCCAACATAACCCCATCATCATGAACGTCGCCGACAACAACGCCGACCCGTTCAGCACCGACGAGTCGGACTGGCCGCTCCGCAACTTTGAAGAGGTCTACATGGTA
[11]SJ15673
[10]SJ15673
[9]SJ15673
[7]SJ15673
[6]SJ15673
[5]SJ15673
[4]SJ15673
[3]SJ15673
[8]SJ15673
[2]SJ15673
[11]SJ15673
[10]SJ15673
[9]SJ15673
[8]SJ15673
[6]SJ15673
[5]SJ15673
[4]SJ15673
[3]SJ15673
[2]SJ15673
[7]SJ15673
[11]SJ15673
[10]SJ15673
[7]SJ15673
[5]SJ15673
[4]SJ15673
[3]SJ15673
[8]SJ15673
[9]SJ15673
[6]SJ15673
[2]SJ15673

Fig. S33

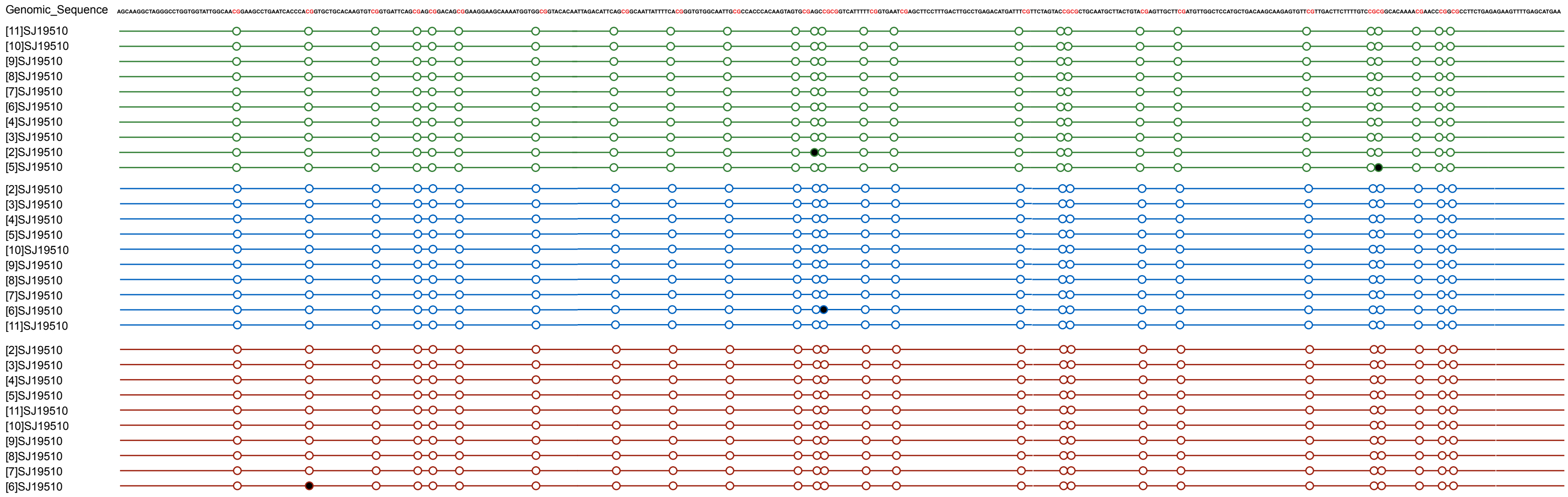
Genomic_Sequence
[11]SJ19510
[10]SJ19510
[9]SJ19510
[8]SJ19510
[7]SJ19510
[6]SJ19510
[4]SJ19510
[3]SJ19510
[2]SJ19510
[5]SJ19510
[2]SJ19510
[3]SJ19510
[4]SJ19510
[5]SJ19510
[10]SJ19510
[9]SJ19510
[8]SJ19510
[7]SJ19510
[6]SJ19510
[11]SJ19510
[2]SJ19510
[3]SJ19510
[4]SJ19510
[5]SJ19510
[11]SJ19510
[10]SJ19510
[9]SJ19510
[8]SJ19510
[7]SJ19510
[6]SJ19510

Fig. S34

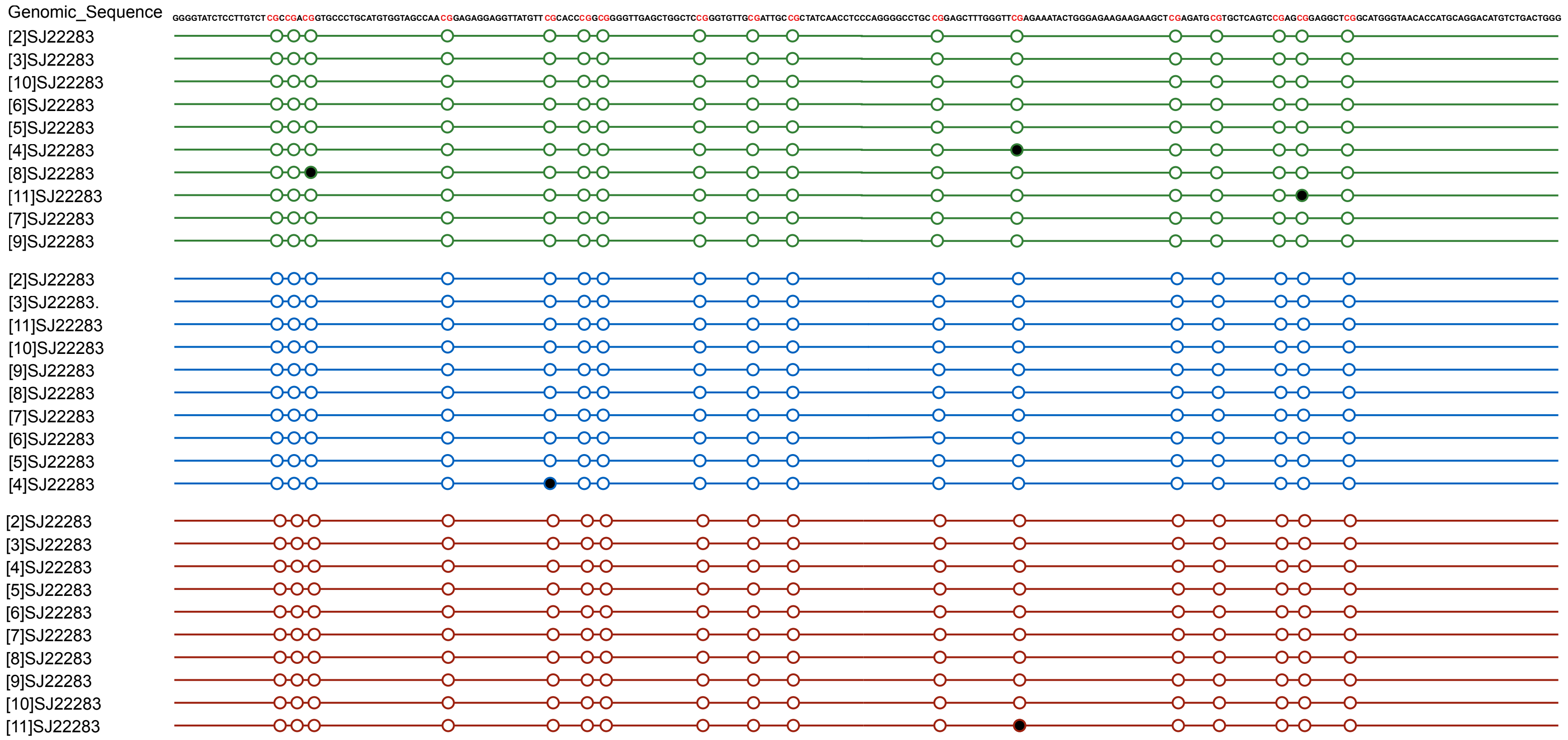
Genomic_Sequence
GGGGTATCTCCTTGTCTCGCCGACGGTGCCCTGCATGTGGTAGCCAACGGAGAGGAGGTTATGTTCGCACCCGGCGGGGTTGAGCTGGCTCCGGGTGTTGCGATTGCCGCTATCAACCTCCCAGGGGCCTGCCGGAGCTTTGGGTTCGAGAAATACTGGGAGAAGAAGAAGCTCGAGATGCGTGCTCAGTCCGAGCGGAGGCTCGGCATGGGTAACACCATGCAGGACATGTCTGACTGGG
[2]SJ22283
[3]SJ22283
[10]SJ22283
[6]SJ22283
[5]SJ22283
[4]SJ22283
[8]SJ22283
[11]SJ22283
[7]SJ22283
[9]SJ22283
[2]SJ22283
[3]SJ22283.
[11]SJ22283
[10]SJ22283
[9]SJ22283
[8]SJ22283
[7]SJ22283
[6]SJ22283
[5]SJ22283
[4]SJ22283
[2]SJ22283
[3]SJ22283
[4]SJ22283
[5]SJ22283
[6]SJ22283
[7]SJ22283
[8]SJ22283
[9]SJ22283
[10]SJ22283
[11]SJ22283

Fig. S35

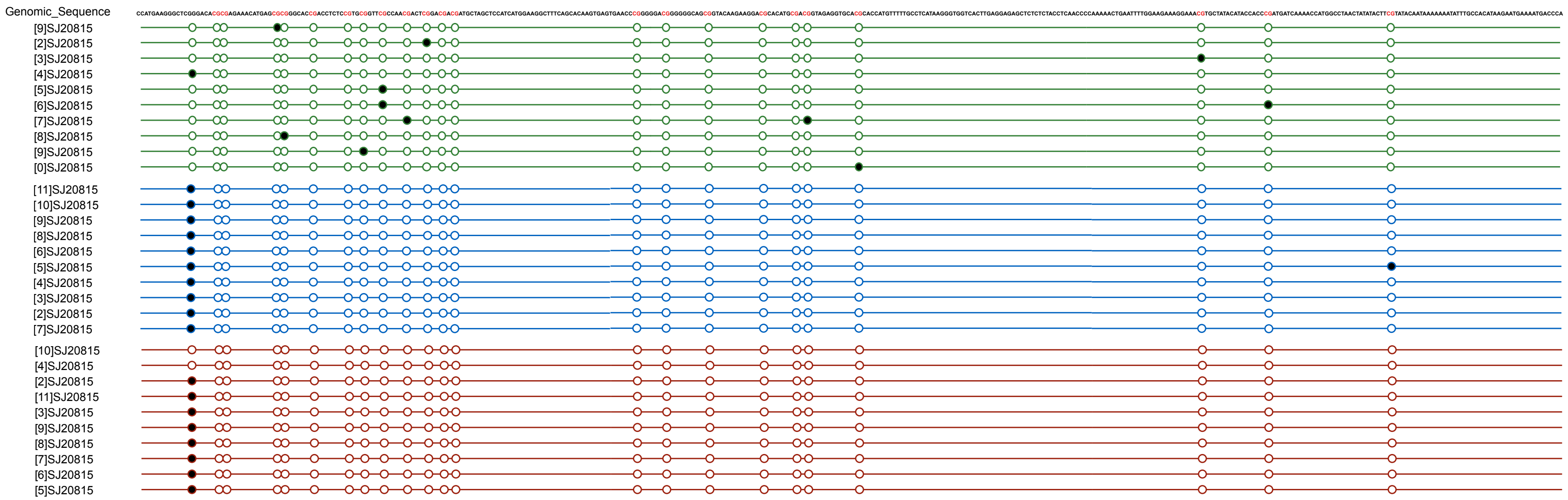
Genomic_Sequence
[9]SJ20815
[2]SJ20815
[3]SJ20815
[4]SJ20815
[5]SJ20815
[6]SJ20815
[7]SJ20815
[8]SJ20815
[9]SJ20815
[0]SJ20815
[11]SJ20815
[10]SJ20815
[9]SJ20815
[8]SJ20815
[6]SJ20815
[5]SJ20815
[4]SJ20815
[3]SJ20815
[2]SJ20815
[7]SJ20815
[10]SJ20815
[4]SJ20815
[2]SJ20815
[11]SJ20815
[3]SJ20815
[9]SJ20815
[8]SJ20815
[7]SJ20815
[6]SJ20815
[5]SJ20815

Fig. S36

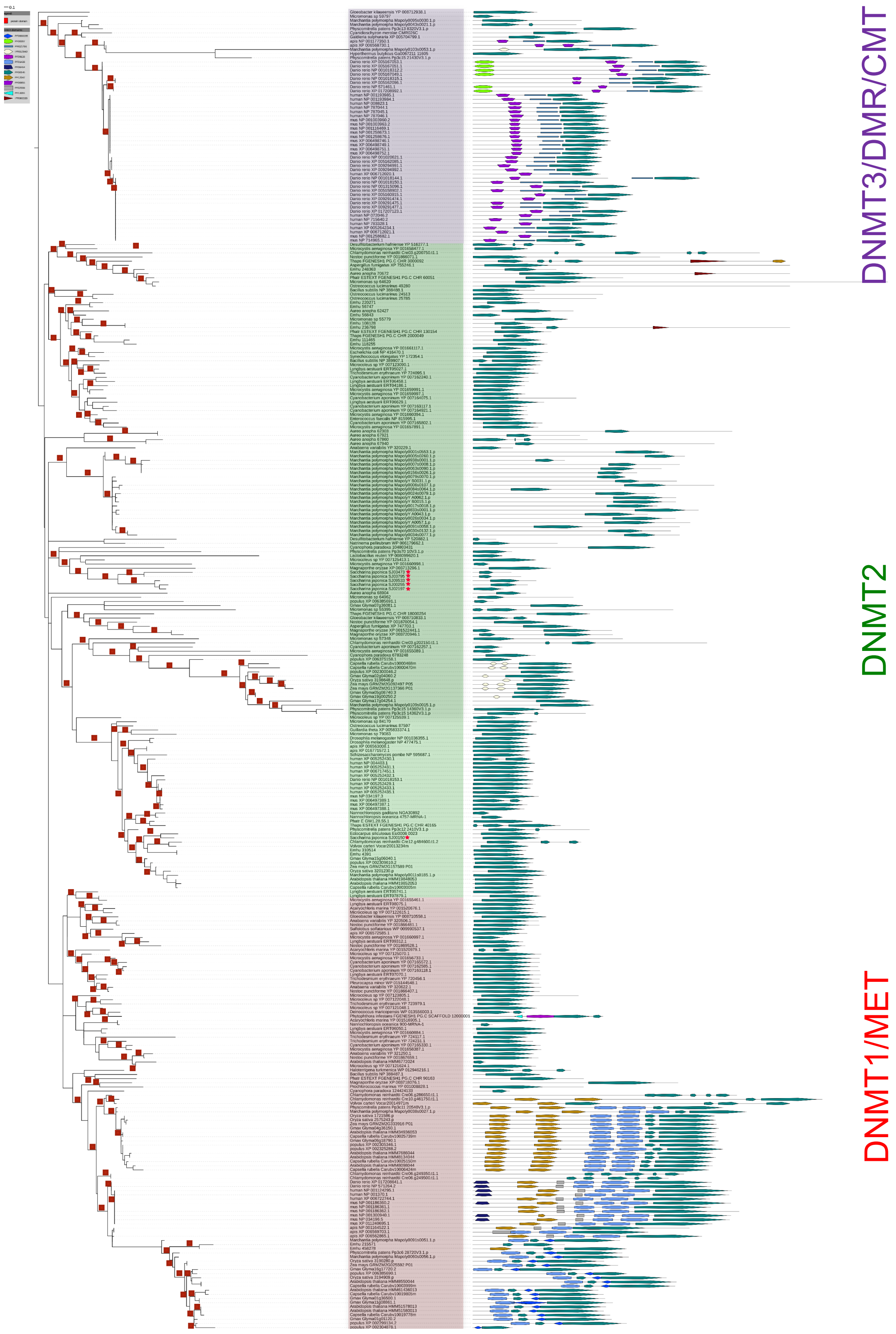

Fig. S37

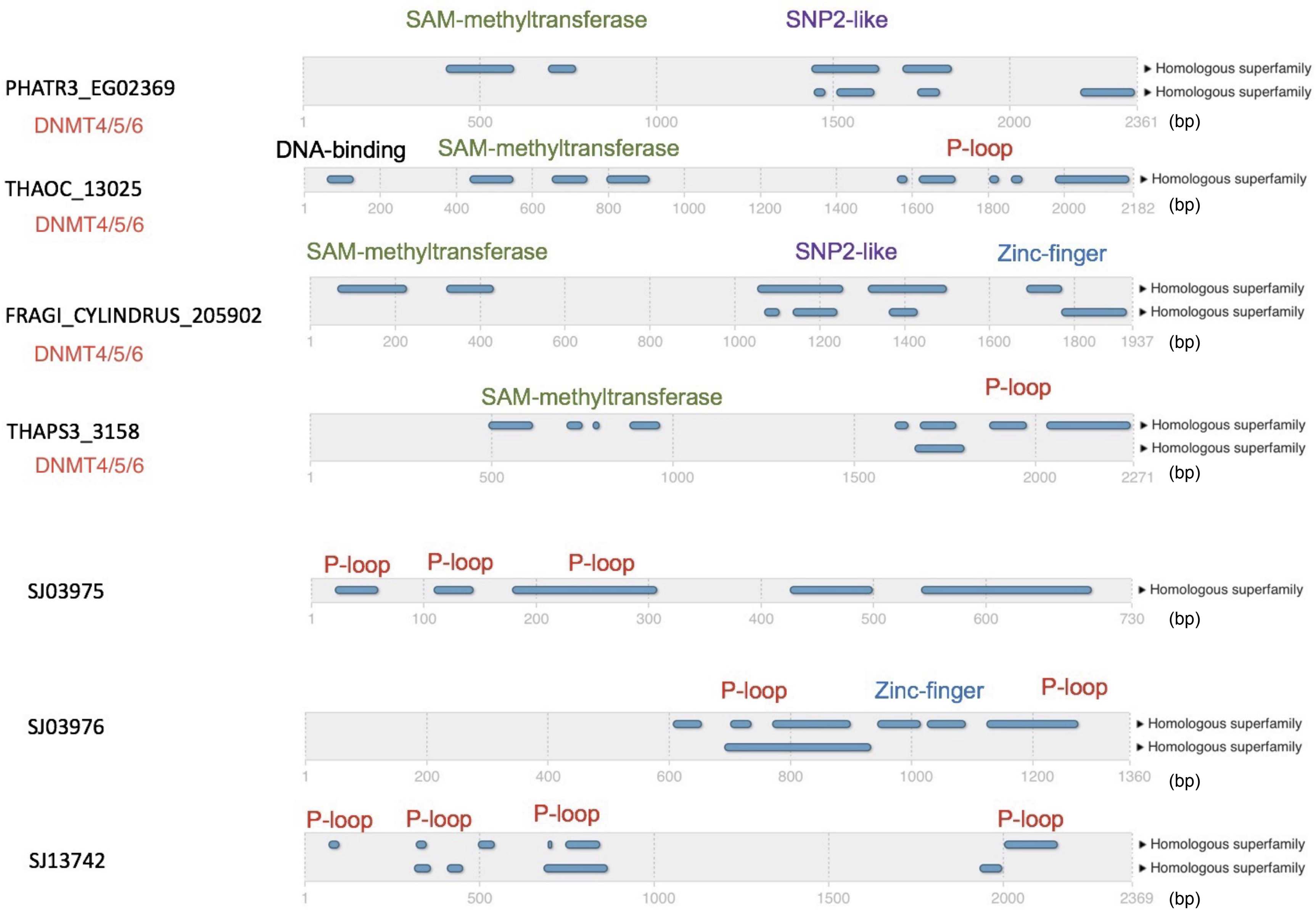
SAM-methyltransferase
SNP2-like
PHATR3_EG02369
DNMT4/5/6
Homologous superfamily
Homologous superfamily
1 500 1000 1500 2000 2361 (bp)
DNA-binding
SAM-methyltransferase
P-loop
THAOC_13025
DNMT4/5/6
Homologous superfamily
1 200 400 600 800 1000 1200 1400 1600 1800 2000 2182 (bp)
SAM-methyltransferase
SNP2-like
Zinc-finger
FRAGI_CYLINDRUS_205902
DNMT4/5/6
Homologous superfamily
Homologous superfamily
1 200 400 600 800 1000 1200 1400 1600 1800 1937 (bp)
SAM-methyltransferase
P-loop
THAPS3_3158
DNMT4/5/6
Homologous superfamily
Homologous superfamily
1 500 1000 1500 2000 2271 (bp)
P-loop
P-loop
P-loop
SJ03975
Homologous superfamily
1 100 200 300 400 500 600 730 (bp)
P-loop
Zinc-finger
P-loop
SJ03976
Homologous superfamily
Homologous superfamily
1 200 400 600 800 1000 1200 1360 (bp)
P-loop
P-loop
P-loop
P-loop
SJ13742
Homologous superfamily
Homologous superfamily
1 500 1000 1500 2000 2369 (bp)

Fig. S38

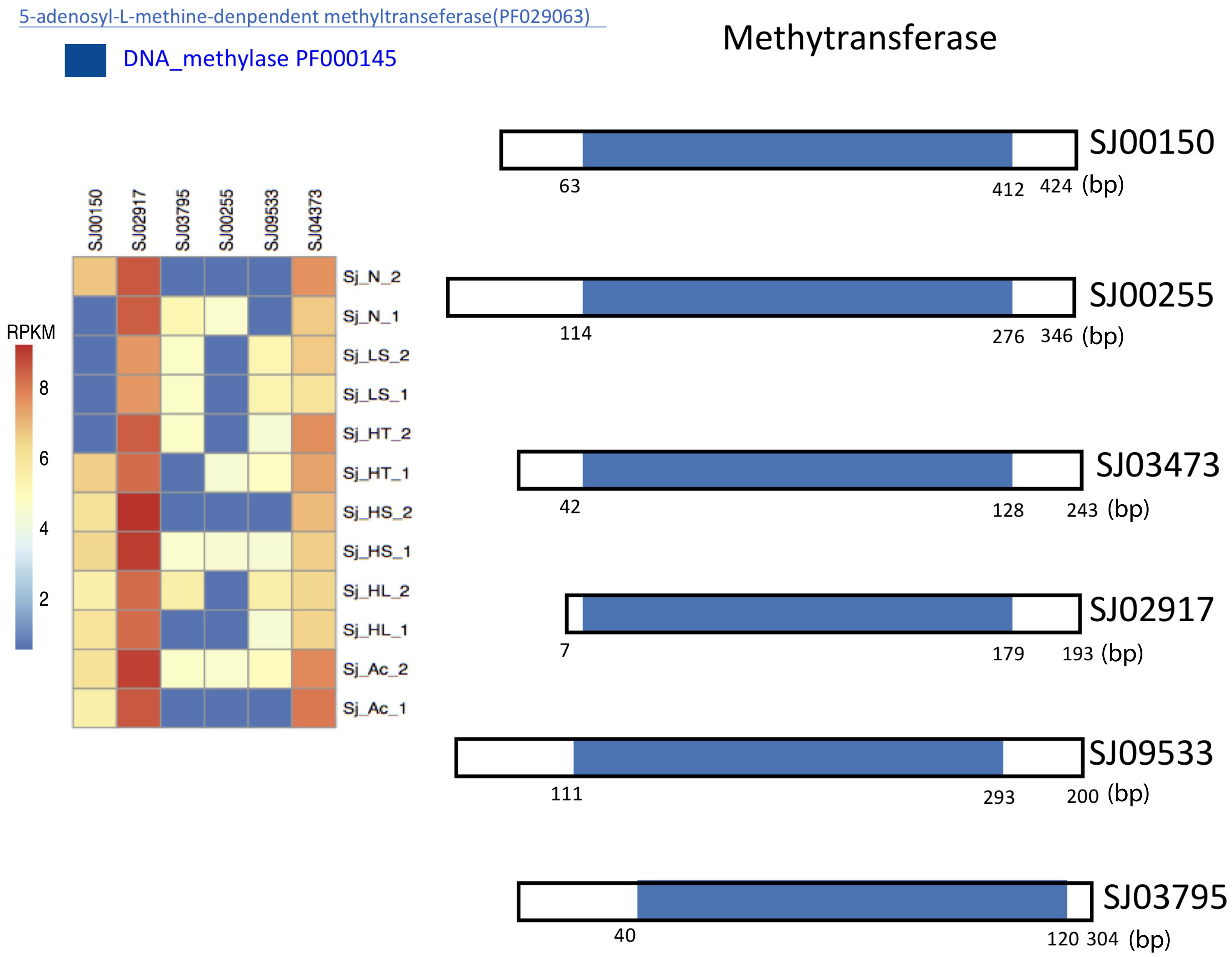
5-adenosyl-L-methine-denpendent methyltranseferase(PF029063)
DNA_methylase PF000145
Methytransferase
SJ00150 SJ02917 SJ03795 SJ00255 SJ09533 SJ04373
Sj_N_2
Sj_N_1
Sj_LS_2
Sj_LS_1
Sj_HT_2
Sj_HT_1
Sj_HS_2
Sj_HS_1
Sj_HL_2
Sj_HL_1
Sj_Ac_2
Sj_Ac_1
RPKM
8
6
4
2
SJ00150
63 412 424 (bp)
SJ00255
114 276 346 (bp)
SJ03473
42 128 243 (bp)
SJ02917
7 179 193 (bp)
SJ09533
111 293 200 (bp)
SJ03795
40 120 304 (bp)

Fig. S39

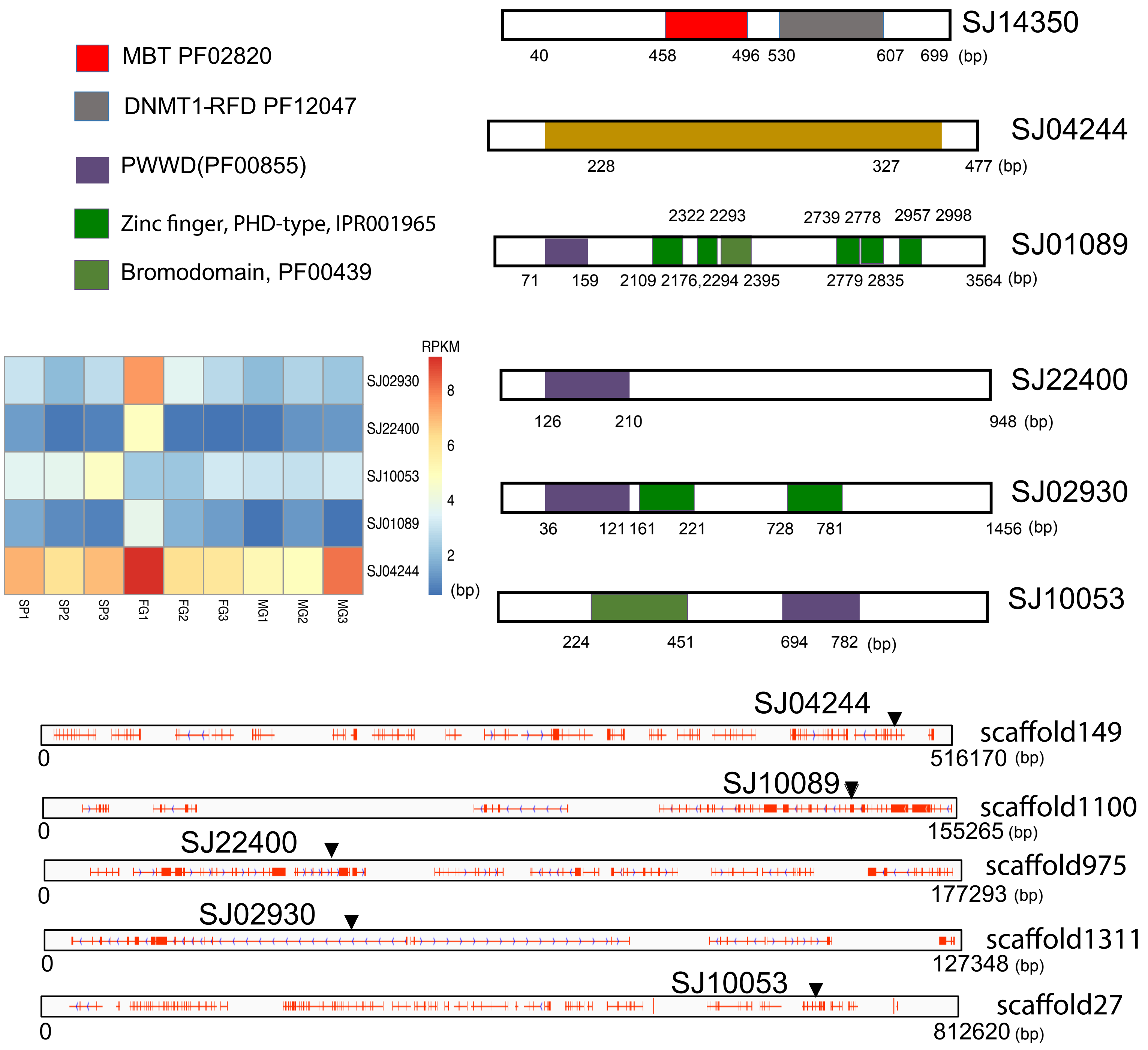
MBT PF02820
DNMT1-RFD PF12047
PWWD(PF00855)
Zinc finger, PHD-type, IPR001965
Bromodomain, PF00439
SJ14350
40 458 496 530 607 699 (bp)
SJ04244
228 327 477 (bp)
SJ01089
2322 2293 2739 2778 2957 2998
71 159 2109 2176,2294 2395 2779 2835 3564 (bp)
RPKM
8
6
4
2
(bp)
SJ02930
SJ22400
SJ10053
SJ01089
SJ04244
SP1 SP2 SP3 FG1 FG2 FG3 MG1 MG2 MG3
SJ22400
126 210 948 (bp)
SJ02930
36 121 161 221 728 781 1456 (bp)
SJ10053
224 451 694 782 (bp)
SJ04244
scaffold149
0 516170 (bp)
SJ10089
scaffold1100
0 155265 (bp)
SJ22400
scaffold975
0 177293 (bp)
SJ02930
scaffold1311
0 127348 (bp)
SJ10053
scaffold27
0 812620 (bp)

Fig. S40

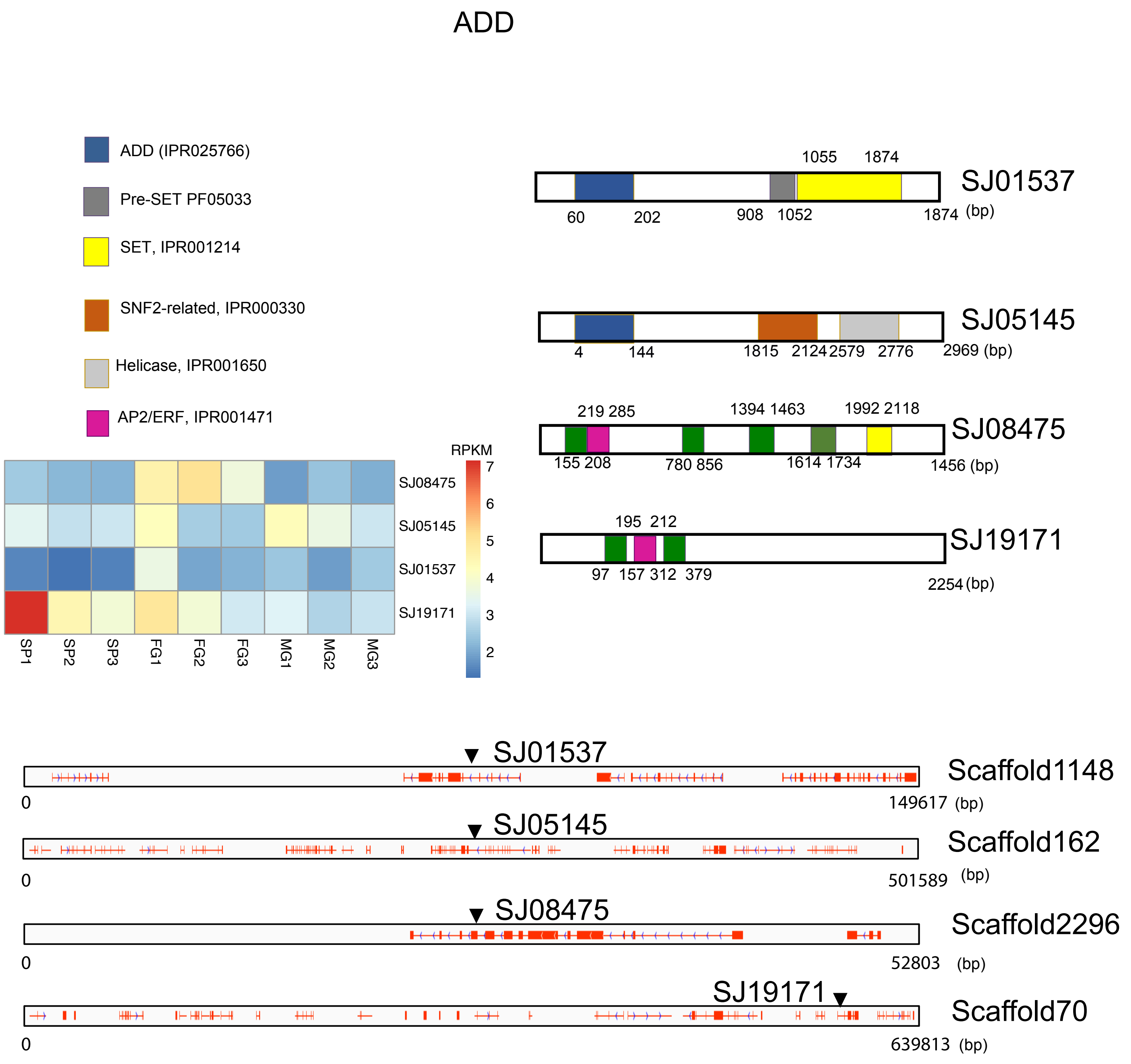
ADD
ADD (IPR025766)
Pre-SET PF05033
SET, IPR001214
SNF2-related, IPR000330
Helicase, IPR001650
AP2/ERF, IPR001471
1055 1874
SJ01537
60 202 908 1052 1874 (bp)
SJ05145
4 144 1815 2124 2579 2776 2969 (bp)
219 285 1394 1463 1992 2118
SJ08475
155 208 780 856 1614 1734 1456 (bp)
195 212
SJ19171
97 157 312 379
2254 (bp)
RPKM
7
6
5
4
3
2
SJ08475
SJ05145
SJ01537
SJ19171
SP1
SP2
SP3
FG1
FG2
FG3
MG1
MG2
MG3
SJ01537
Scaffold1148
0
149617 (bp)
SJ05145
Scaffold162
0
501589 (bp)
SJ08475
Scaffold2296
0
52803 (bp)
SJ19171
Scaffold70
0
639813 (bp)

Fig. S41

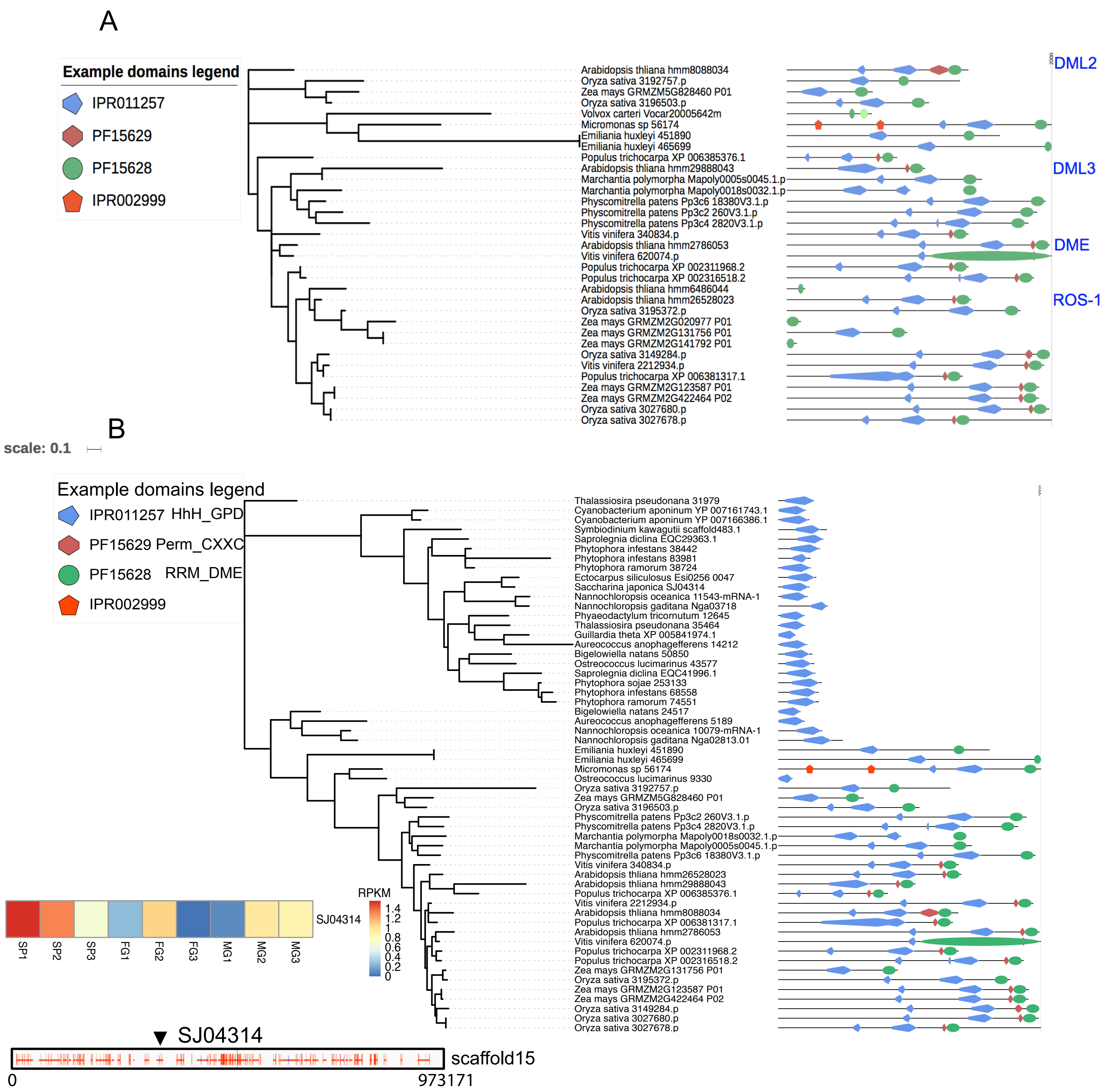
A
Example domains legend
IPR011257
PF15629
PF15628
IPR002999
Arabidopsis thliana hmm8088034
Oryza sativa 3192757.p
Zea mays GRMZM5G828460 P01
Oryza sativa 3196503.p
Volvox carteri Vocar20005642m
Micromonas sp 56174
Emiliania huxleyi 451890
Emiliania huxleyi 465699
Populus trichocarpa XP 006385376.1
Arabidopsis thliana hmm29888043
Marchantia polymorpha Mapoly0005s0045.1.p
Marchantia polymorpha Mapoly0018s0032.1.p
Physcomitrella patens Pp3c6 18380V3.1.p
Physcomitrella patens Pp3c2 260V3.1.p
Physcomitrella patens Pp3c4 2820V3.1.p
Vitis vinifera 340834.p
Arabidopsis thliana hmm2786053
Vitis vinifera 620074.p
Populus trichocarpa XP 002311968.2
Populus trichocarpa XP 002316518.2
Arabidopsis thliana hmm6486044
Arabidopsis thliana hmm26528023
Oryza sativa 3195372.p
Zea mays GRMZM2G020977 P01
Zea mays GRMZM2G131756 P01
Zea mays GRMZM2G141792 P01
Oryza sativa 3149284.p
Vitis vinifera 2212934.p
Populus trichocarpa XP 006381317.1
Zea mays GRMZM2G123587 P01
Zea mays GRMZM2G422464 P02
Oryza sativa 3027680.p
Oryza sativa 3027678.p
DML2
DML3
DME
ROS-1
scale: 0.1
B
Example domains legend
IPR011257 HhH_GPD
PF15629 Perm_CXXC
PF15628 RRM_DME
IPR002999
Thalassiosira pseudonana 31979
Cyanobacterium aponinum YP 007161743.1
Cyanobacterium aponinum YP 007166386.1
Symbiodinium kawagutii scaffold483.1
Saprolegnia diclina EQC29363.1
Phytophora infestans 38442
Phytophora infestans 83981
Phytophora ramorum 38724
Ectocarpus siliculosus Esi0256 0047
Saccharina japonica SJ04314
Nannochloropsis oceanica 11543-mRNA-1
Nannochloropsis gaditana Nga03718
Phyaeodactylum tricornutum 12645
Thalassiosira pseudonana 35464
Guillardia theta XP 005841974.1
Aureococcus anophagefferens 14212
Bigelowiella natans 50850
Ostreococcus lucimarinus 43577
Saprolegnia diclina EQC41996.1
Phytophora sojae 253133
Phytophora infestans 68558
Phytophora ramorum 74551
Bigelowiella natans 24517
Aureococcus anophagefferens 5189
Nannochloropsis oceanica 10079-mRNA-1
Nannochloropsis gaditana Nga02813.01
Emiliania huxleyi 451890
Emiliania huxleyi 465699
Micromonas sp 56174
Ostreococcus lucimarinus 9330
Oryza sativa 3192757.p
Zea mays GRMZM5G828460 P01
Oryza sativa 3196503.p
Physcomitrella patens Pp3c2 260V3.1.p
Physcomitrella patens Pp3c4 2820V3.1.p
Marchantia polymorpha Mapoly0018s0032.1.p
Marchantia polymorpha Mapoly0005s0045.1.p
Physcomitrella patens Pp3c6 18380V3.1.p
Vitis vinifera 340834.p
Arabidopsis thliana hmm26528023
Arabidopsis thliana hmm29888043
Populus trichocarpa XP 006385376.1
Vitis vinifera 2212934.p
Arabidopsis thliana hmm8088034
Populus trichocarpa XP 006381317.1
Arabidopsis thliana hmm2786053
Vitis vinifera 620074.p
Populus trichocarpa XP 002311968.2
Populus trichocarpa XP 002316518.2
Zea mays GRMZM2G131756 P01
Oryza sativa 3195372.p
Zea mays GRMZM2G123587 P01
Zea mays GRMZM2G422464 P02
Oryza sativa 3149284.p
Oryza sativa 3027680.p
Oryza sativa 3027678.p
RPKM
1.4
1.2
1
0.8
0.6
0.4
0.2
0
SJ04314
SP1
SP2
SP3
FG1
FG2
FG3
MG1
MG2
MG3
SJ04314
scaffold15
0
973171

Fig. S42

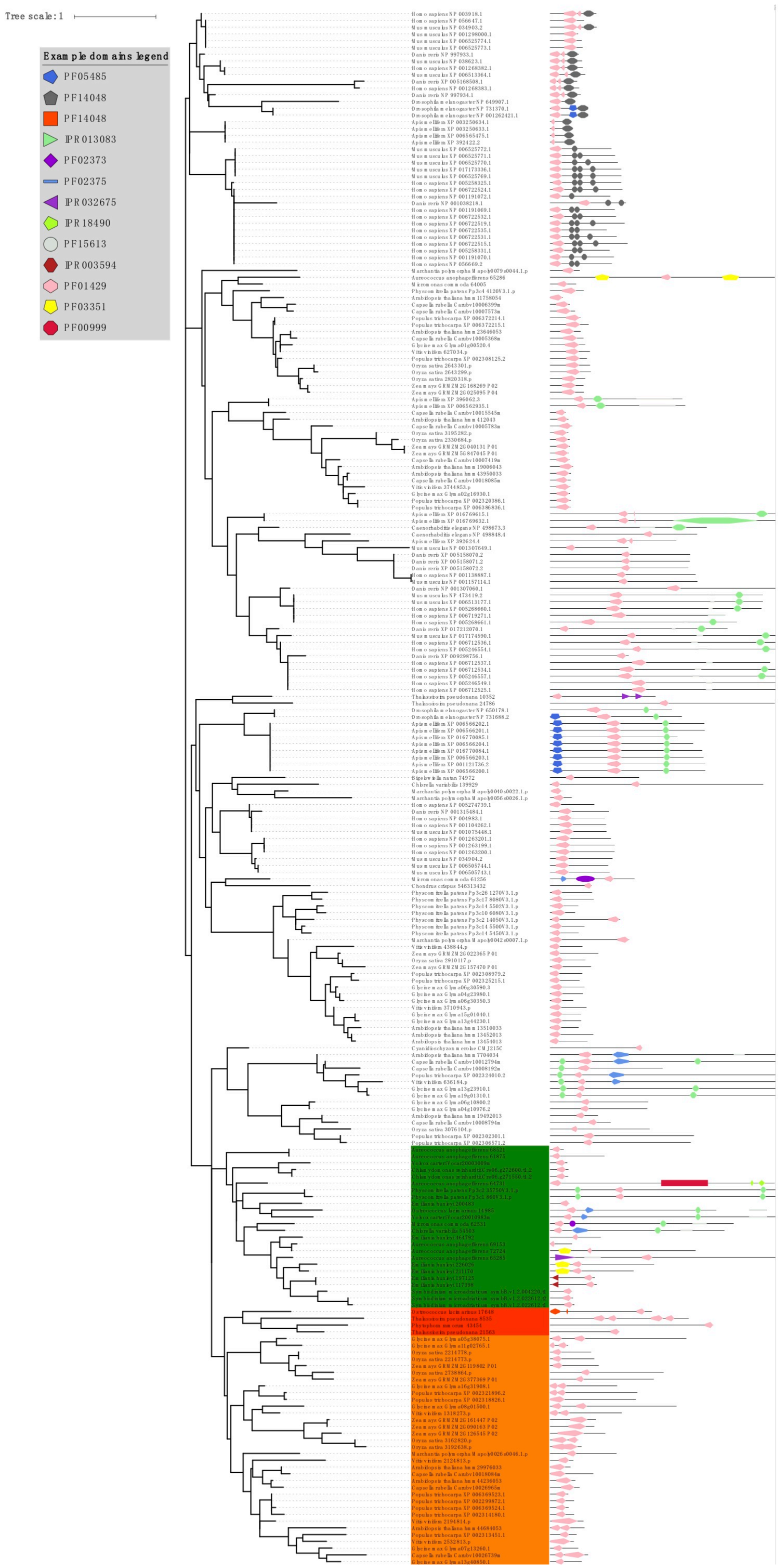

MBD domain:
methyl-CpG-binding domain

Methylation at CpG dinucleotide, the most common DNA modification in eukaryotes, has been correlated with gene silencing associated with various phenomena such as genomic imprinting, transposon and chromosome X inactivation, differentiation, and cancer. Effects of DNA methylation are mediated through proteins which bind to symmetrically methylated CpGs. Such proteins contain a specific domain of ~70 residues, the methyl-CpG-binding domain (MBD), which is linked to additional domains associated with chromatin, such as the bromodomain, the AT hook motif, The SET domain, or the PHD finger. MBD-containing proteins appear to act as structural proteins, which recruit a variety of histone deacetylase (HDAC) complexes and chromatin remodelling factors, leading to chromatin compaction and, consequently, to transcriptional repression. The MBD of MeCP2, MBD1, MBD2, MBD4 and BAZ2 mediates binding to DNA, in case of MeCP2, MBD1 and MBD2 preferentially to methylated CpG.

Fig. S43

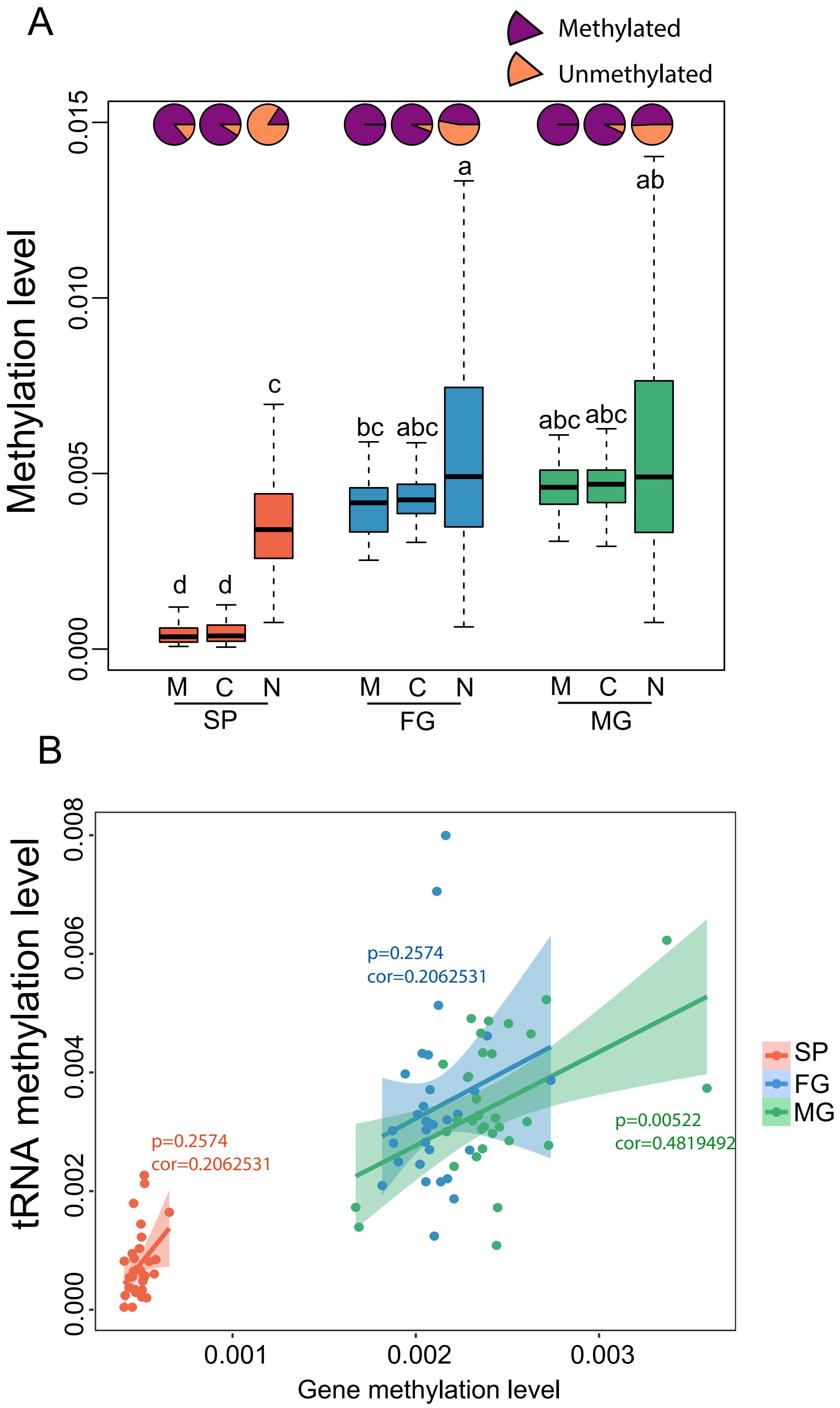
A
Methylated
Unmethylated
Methylation level
0.000
0.005
0.010
0.015
d
d
c
bc
abc
a
abc
abc
ab
M
C
N
SP
M
C
N
FG
M
C
N
MG
B
tRNA methylation level
0.000
0.002
0.004
0.006
0.008
p=0.2574
cor=0.2062531
p=0.2574
cor=0.2062531
p=0.00522
cor=0.4819492
SP
FG
MG
0.001
0.002
0.003
Gene methylation level
