# Supplementary material for: Single‐base methylome profiling of the giant kelp Saccharina japonica reveals significant differences in DNA methylation to microalgae and plants
Source: New Phytol. 2019 Sep 27;225(1):234–49. doi: 10.1111/nph.16125 (PMC6916402; doi:10.1111/nph.16125)
Supplement: Supplementary file 27 — Methods S1 1, Constructing the pseudo‐chromosomes using a genetic linkage map; 2, methylation profiles of sex‐determining regions and their gene expression; 3, DNA methyltransferases evolution and their putative function; 4, other methods in this article. [file NPH-225-234-s027.pdf]

Supporting Information

Title:

Single-base methylome profiling of the giant kelp *Saccharina japonica* reveals significant differences in DNA methylation to microalgae and plants

Authors:

Xiao Fan[1,2,3], Wentao Han[1], Linhong Teng[1,4,5], Peng Jiang[6], Xiaowen Zhang[1], Dong Xu[1], Chang Li[7], Matteo Pellegrini[8], Chunhui Wu[6], Yitao Wang[1], Michelle Joyce Slade Kaczurowski[9], Xin Lin[10], Leila Tirichine[11], Thomas Mock[12*], Naihao Ye[1,2*]

Affiliations:

[1]Yellow Sea Fisheries Research Institute, Chinese Academy of Fishery Sciences, Qingdao, China

[2]Function Laboratory for Marine Fisheries Science and Food Production Processes, Qingdao, China

[3]Key Laboratory of Exploration and Utilization of Aquatic Genetic Resources, Ministry of Education, Shanghai Ocean University, Shanghai 201306, China

[4]College of Life Science, Dezhou University, Dezhou 253023, China

[5]Shandong Key Laboratory of Biophysics, Dezhou University, Dezhou 253023, China

[6]Chinese Academy of Sciences, Qingdao 266071, China.

[7]BGI Education Center, University of Chinese Academy of Sciences, Shenzhen, China

[8]Department of Molecular, Cell and Developmental Biology, and Institute for Genomics and Proteomics, University of California, Los Angeles, CA 90095, USA

[9]Biological Sciences, Flinders University, GPO Box 2100, Adelaide, SA 5001, Australia

[10]State Key Laboratory of Marine Environmental Science, College of Ocean & Earth Sciences, Xiamen University, China

[11]CNRS UMR 6286, Faculté des Sciences et des Techniques, Université de Nantes, 2 rue de la Houssinière, 44322, Nantes, France

[12]School of Environmental Sciences, University of East Anglia, Norwich Research Park, NR4 7TJ Norwich, UK

Corresponding authors[*]:

N. Ye: yenh@ysfri.ac.cn; T. Mock: t.mock@uea.ac.uk

Article acceptance date:

6 August 2019

Keywords:

DNA methylation, WGBS-seq, MeDIP-seq, BS-PCR, brown algae, life-cycle stages, gene expression, DNMT2

Supplementary Methods I:

Part 1: Constructing the pseudo-chromosomes using a genetic linkage map

4994 markers of 31 linkage groups from the genetic linkage map analysis (1) were integrated into the assembled *Saccharina japonica* scaffolds to construct a physical map at the chromosomal level. All markers were aligned against the assembled kelp scaffolds using BLASTN (2) with an e-value cut-off 1E-15. Markers that mapped to more than one scaffold (150x) were discarded. A total of 88 scaffolds had markers from more than one linkage group, which could have been either the consequence of incorrect assembly or an incorrect linkage group. To correct the incompatibility between the physical map and linkage groups, reads from two mate-paired libraries (3kb and 5kb) were aligned to the assembled scaffolds with BWA (3). If the incompatible positions on scaffolds were not covered by mate-paired reads, the scaffold was splited at the gaps (intergenic). If the incompatible positions were mapping to paired-end reads, we selected the linkage group with a larger number of markers and discarded markers in other linkage groups. Six scaffolds were split and 4687 markers were used to construct the physical map of *S. japonica.*

The order of scaffolds on chromosomes were sorted by mean value of markers on the same scaffolds. In order to determine the orientation of scaffolds, we compared the physical order of the markers with their linkage-map order. If the markers on a scaffold had the same order between the physical and the linkage map order, the orientation of the scaffold was considered in positive orientation and *vice versa*. At the end, scaffolds were concatenated into chromosomes with 200 bp gaps.

A total of 1,576 scaffold were anchored to the genetic map, accounting for 64.69 % (352.93 Mbp) of the assembled kelp scaffolds. The remaining scaffolds were used to construct the artificial chromosome 0.

Part 2: Methylation profiles of sex-determining regions and their gene expression

Sex-determining regions were well studied in XY and ZW systems. Similar sex-determining processes were recently reported for the UV system that brown algae possess (4). To study the influence of DNA methylation on sex determination in *S. japonica*, we looked at genes and their regulatory elements involved in the UV system in relation to their expression and MLgf (Fig. S20, Table S23). In *S. japonica*, there are 18 genes known to be involved in sex determination of which, 12 are located in the sex-determining region (SDR) and 6 have moved out to auto-chromosomes (named as PAR) (Fig. S20). Of the 12 genes located in the SDR, 6 are for female and 6 are for male gametophytes. Interestingly, 4 genes that left the SDR were highly expressed in SP and had a significantly negative correlation with their MLgf in all life-cycle stages. These four genes include a MEMO domain (SJ13722), a glycosyltransferase family (PF01697, SJ18945), a RING-type zinc finger (15797) and

a Rab-GTPase-TBC domain (PF00566, SJ15874). The male genes inside the SDR show no correlation between the MLgf and their expression. However, they have a much lower MLgf in FG than in MG (Fig. S20).

Part 3: DNA methyltransferases evolution and their putative function in *S. japonica*

Recent rapid advances in next-generation sequencing technologies have enabled the establishment of methylation maps at a single-base resolution (6). To date, whole-genome methylomes have been reported from a range of most major eukaryotic groups (7) such as the early-diverging vascular plants *Selaginella moellendorffii*, the moss *Physcomitrella patens*, the silkworm *Bombyx mori* and honey bee *Apis mellifera* (8). Furthermore, several model eukaryotes are devoid of DNA methylation altogether including the yeast *Saccharomyces cerevisiae*, the nematode *Caenorhabditis elegans*, the fruit fly *Drosophila melanogaster* (except in the early stages of embryogenesis) and the brown alga *Ectocarpus siliculosus* (7). DNA methyltransferases (DNMTs or DNA MTases) usually methylated DNA in all of these organisms. These enzymes catalyze the transfer of the methyl group from a cofactor molecule S-adenosyl-l-methionine (AdoMet or SAM) to the C5 position of the cytosine residues. To uncover DNMTs in *S. japonica*, we identified and compared the DNMTs family of *S. japonica* with DNMTs from other eukaryotes. Potential candidates were identified and downloaded by using a hidden Markov model (HMM) corresponding to the C5-cytosine–specific DNA MTase domain in Pfam (9) (PF00145, named DNA_methylase in http://pfam.xfam.org). All proteins with a score above 125 were regarded as members of the MTase family. This threshold was used instead of the default cutoff defined in Pfam (http://pfam.xfam.org) in order to increase the sensitivity. The presence of the crucial MTase domain was verified by using the Interpro database (10). For assessing phylogenetic relationships, only MTase domains were used to construct phylogenetic trees. Over 50 species with approximately 130 DNMTsbwere clustered into three main clades based on the MTase domain. Six different DNMTs were identified in *S. japonica* and all of them seem to belong to the DNMT2 family (Fig. 4*A*). To shed further light on the structure of DNMT2 in *S. japonica,* we identified every domain of the human DNMT3 and DNMT1 genes to see if any of those domains were associated with the DNMT2 domains in *S. japonica* (Fig. S26). Genes containing PWWP (PF00855) and ADD (IPR025766) were found in *S. japonica*, together with domains PHD (IPR001965) and the Bromodomain (PF00439). However, none of these domains were associated with any of the DNMT2 domains in *S. japonica* (11), (13), (Fig. S25-27).

However, DNA methylation can also be influenced by processes that demethylate either passively or actively by demethylases. For instance, a passive loss of 5mC can be achieved through successive cycles of DNA replication in the absence of functional DNMT1/MET1 (16,17). Furthermore, demeter (DME) / demeter-like1-3

(DML) / repressor of silencing 1 (ROS) family of DNA glycosylases could rapidly erase 5mC independent of DNA replication in specific biological settings in plants as they share the RRM-fold domain (PF15628) and DNA glycosylases domain (IPR011257), the first one is reported to catalyze the removal of the 5mC base and subsequently cleave the backbone through lyase activity and the second act to repair oxidative damage in DNA (16, 18-21). The methyl-CpG-binding domain protein 2 (MBD2, PF01429) was also reported to be able to catalyze the reaction of removal the methyl group (16). To uncover proteins involved in active DNA demethylation, we searched for DME, DML, ROS and MBD domains in coding genes of *S. japonica*. We found an RRM fold domain and a DNA glycosylase domain (Fig. S28, S29).

Part 4: Other Methods

DNA methylation

Quality control

We use FastQC to perform basic statistics on the quality of the raw reads. Then, those read sequences produced by the Illumina pipeline in FASTQ format were pre-processed through Trimmomatic software which can be summarized as below:
(1) Remove low-quality reads: scan the read with a 4-base wide sliding window, cutting when the average quality per base drops below 15 (SLIDINGWINDOW: 4:15);
(2) Remove leading low quality or N bases (below quality 3) and trailing low quality or N bases (below quality 3) (LEADING:3, TRAILING:3);
(3) Remove adapters: there are two modes to remove the adapter sequence: a. alignment with the adapter sequence, the number of matching bases were greater than 10 and mismatch=2; b. when read1 and read2 overlapping base scoring greater than 30, removed non-overlapping portions (ILLUMINACLIP: adapter.fa: 2: 30: 10);
(4) Drop reads below the 36 bases long;
(5) Discard those reads that cannot form pairs;
The remaining reads that passed all the filtering steps were counted as clean reads and all subsequent analyses were based on them. We use FastQC to perform basic statistics on the quality of the final reads.

Reads mapping to the reference genome

Bismark software (22) was used to perform alignments of bisulfite-treated reads to a reference genome using default parameters. The reference genome was transformed to a bisulfite-converted version (C-to-T and G-to-A converted) and then indexed using bowtie2 (23). Sequence reads were also transformed into fully bisulfite-converted versions (C-to-T and G-to-A converted) before they were aligned to similarly converted versions of the genome in a directional manner. Sequence reads that produced a unique best alignment from the two alignment processes (original top and

bottom strand) were then compared to the genomic sequence and the methylation state of all cytosine positions in the reads were inferred. Read pairs that shared the same coordinates in genome were regarded as duplicated ones, which were removed before methylation state calling was conducted, thus avoiding a potential methylation level calculation bias. The results of the methylation extractor were transformed to bigWig format for visualizations using IGV browser. The bisulfite non-conversion rate was calculated as the percentage of cytosines sequenced at cytosine reference positions in the lambda genome.

Transcriptome analysis

Sequencing libraries were generated using the rRNA-depleted RNA by NEBNext® UltraTM Directional RNA Library Prep Kit for Illumina® (NEB, USA) following manufacturer's recommendations. Briefly, fragmentation was carried out using divalent cations under elevated temperature in NEBNext First Strand Synthesis Reaction Buffer (5X). First-strand cDNA was synthesized using random hexamer primer and M-MuLV Reverse Transcriptase (RNaseH). Second-strand cDNA synthesis was subsequently performed using DNA Polymerase I and RNase H. In the reaction buffer, dNTPs with dTTP were replaced by dUTP. Remaining overhangs were converted into blunt ends via exonuclease/polymerase activities. After adenylation of 3' ends of DNA fragments, NEBNext Adaptor with hairpin loop structure were ligated to prepare for hybridization. In order to select cDNA fragments of preferentially 150~200 bp in length, the library fragments were purified with AMPure XP system (Beckman Coulter, Beverly, USA). Then 3μl USER Enzyme (NEB，USA) was used with size-selected, adaptor-ligated cDNA at 37°C for 15 min followed by 5 min at 95°C before PCR. Then PCR was performed with Phusion High-Fidelity DNA polymerase, Universal PCR primers and Index (X) Primer. At last, products were purified (AMPure XP system) and library quality was assessed on the Agilent Bioanalyzer 2100 system. After cluster generation, the libraries were sequencing on an illumine Hiseq 2500 platform and 125bp paired-end reads were generated. After quality control of the raw reads, clean data were mapped to the reference genome using Bowtie v2.0.6 and TopHat v2.0.9(25). The mapped reads of each sample were assembled by both Scripture (beta2) (26) and Cufflinks (v2.1.1) (27) in a reference-based approach.

References

1. Zhang N, *et al.* (2015) Construction of a high density SNP linkage map of kelp (Saccharina japonica) by sequencing Taq I site associated DNA and mapping of a sex determining locus. *BMC Genomics* 16(1):189.
2. Altschul SF, Madden TL, & Schäffer AA (1997) Jinghui Zhang, Zheng Zhang, Webb Miller, and David J. Lipman (1997)," Gapped BLAST and PSI-BLAST: a new generation of protein database search.

3. Li H & Durbin R (2010) Fast and accurate short read alignment with Burrows-Wheeler transform. *Bioinformatics* 25(5):1754-1760.
4. Cokus SJ*, et al.* (2008) Shotgun bisulphite sequencing of the Arabidopsis genome reveals DNA methylation patterning. *Nature* 452(7184):215-219.
5. Lipinska AP*, et al.* (2017) Multiple gene movements into and out of haploid sex chromosomes. *Genome Biology* 18(1):104.
6. Feng S*, et al.* (2010) Conservation and divergence of methylation patterning in plants and animals. *Proceedings of the National Academy of Sciences of the United States of America* 107(19):8689.
7. Takuno S, Ran JH, & Gaut BS (2016) Evolutionary patterns of genic DNA methylation vary across land plants. *Nature Plants* 2(2):15222.
8. Veluchamy A*, et al.* (2014) Insights into the role of DNA methylation in diatoms by genome-wide profiling in Phaeodactylum tricornutum. *Nature communications* 4(7):2091-2091.
9. Punta M*, et al.* (2004) The Pfam protein families database. *Nucleic Acids Research* 32(1):D138.
10. Quevillon E*, et al.* (2005) InterProScan: protein domains identifier. *Nucleic Acids Research* 33(Web Server issue):W116-W120.
11. Ponger L & Li W-H (2005) Evolutionary diversification of DNA methyltransferases in eukaryotic genomes. *Molecular biology and evolution* 22(4):1119-1128.
12. Jurkowska RZ, Jurkowski TP, & Jeltsch A (2011) Structure and function of mammalian DNA methyltransferases. *Chembiochem* 12(2):206–222.
13. Goll MG & Bestor TH (2005) Eukaryotic cytosine methyltransferases. *Annual Review of Biochemistry* 74(74):481.
14. Feng S & Jacobsen SE (2011) Epigenetic modifications in plants: an evolutionary perspective. *Current opinion in plant biology* 14(2):179-186.
15. Dirk W, Francesca P, & Monika JB (2011) Epigenetics in C. elegans: facts and challenges. *Genesis* 49(8):647.
16. Wu H & Zhang Y (2014) Reversing DNA methylation: mechanisms, genomics, and biological functions. *Cell* 156(1-2):45.
17. He XJ, Chen T, & Zhu JK (2011) Regulation and function of DNA methylation in plants and animals. *Cell Research* 21(3):442.
18. Law JA & Jacobsen SE (2010) Establishing, maintaining and modifying DNA methylation patterns in plants and animals. *Nature Reviews Genetics* 11(3):204-220.
19. Ortegagalisteo AP, Moralesruiz T, Ariza RR, & Roldánarjona T (2008) Arabidopsis DEMETER-LIKE proteins DML2 and DML3 are required for appropriate distribution of DNA methylation marks. *Plant Molecular Biology* 67(6):671-681.
20. Ooi SKT & Bestor TH (2008) The Colorful History of Active DNA Demethylation. *Cell* 133(7):1145-1148.

21. Lang Z, *et al.* (2017) Critical roles of DNA demethylation in the activation of ripening-induced genes and inhibition of ripening-repressed genes in tomato fruit. *Proceedings of the National Academy of Sciences*:201705233.
22. Krueger F & Andrews SR (2011) Bismark: a flexible aligner and methylation caller for Bisulfite-Seq applications. *Bioinformatics* 27(11):1571-1572.
23. Langmead B & Salzberg SL (2012) Fast gapped-read alignment with Bowtie 2. *Nature Methods* 9(4):357-359.
24. Wang Z, *et al.* (2015) swDMR: A Sliding Window Approach to Identify Differentially Methylated Regions Based on Whole Genome Bisulfite Sequencing. *Plos One* 10(7).
25. Trapnell C, Pachter L, & Salzberg SL (2009) TopHat: discovering splice junctions with RNA-Seq. *Bioinformatics* 25(9):1105-1111.
26. Guttman M, *et al.* (2010) Ab initio reconstruction of cell type–specific transcriptomes in mouse reveals the conserved multi-exonic structure of lincRNAs. *Nature Biotechnology* 28(5):503-510.
27. Trapnell C, *et al.* (2010) Transcript assembly and quantification by RNA-Seq reveals unannotated transcripts and isoform switching during cell differentiation. *Nature Biotechnology* 28(5):511-515.
28. Memczak S, *et al.* (2014) *Circular RNAs are a large class of animal RNAs with regulatory potency* (Springer International Publishing) pp 333-338.
29. Sun L, *et al.* (2013) Utilizing sequence intrinsic composition to classify protein-coding and long non-coding transcripts. *Nucleic Acids Research* 41(17):e166-e166.
30. Kong L, *et al.* (2007) CPC: assess the protein-coding potential of transcripts using sequence features and support vector machine. *Nucleic Acids Research* 35(Web Server issue):W345.
31. Biswas S & Akey JM (2006) Genomic insights into positive selection. *Trends in Genetics* 22(8):437-446.
32. Chen F, Mackey AJ, Jr SC, & Roos DS (2006) OrthoMCL-DB: querying a comprehensive multi-species collection of ortholog groups. *Nucleic Acids Research* 34(Database issue):363-368.
33. Edgar RC (2004) MUSCLE: Multiple Sequence Alignment with Improved Accuracy and Speed. *Computational Systems Bioinformatics Conference, 2004. Csb 2004. Proceedings*, pp 728-729.
34. Yang Z (2007) Yang ZH.. PAML 4: Phylogenetic analysis by maximum likelihood. Mol Biol Evol 24: 1586-1591. 24(8):1586-1591.

Supporting Information legends to Figs S1-S43 – see separate files for the figures

Fig. S1. Quantify the sequencing libraries constructed using DNA fragments_derived from MeDIP. A) Fluorescence scanning of the sequencing libraries of MeDIP of *S. japonica*. The peak occurs on the excepted range of DNA fragments. B) Fluorescence scanning of the sequencing libraries of MeDIP of bovine which is cited here as control.

Fig. S2. Methylation profiles of *Saccharina japonica* male gametophyte (MG) based on MeDIP-seq. A) Chromosome-specific profiles for *S. japonica* MG methylomes based on MeDIP-seq. For each track, upper panel present the peaks sites along the chromosome; color depth of each peak indicates one's false discovery rate (FDR). Middle panel present the CpG o/e ratio along the chromosome; the down panel present the normalized read depth (NRD) of each 10k bin along chromosome, black line for input sample while green lines for 3 MG samples. B) Normalized absolute read depth (NARD) of all genomic elements including genome wide methylation (GENOME), TSSUP (transcript start site), TESDOWN (transcript stop site), exon, intron, transposable elements (TEs), genes encoding for long non-coding RNAs (lncRNAs), circular RNAs (circRNAs) and tRNAs. See methods for definition of NARD. Statistics among replicates were based on Duncans and Student t-tests, the lowercase letters notations associated with plots indicate the level of significance among different groups. C) Meta-plots of DNA NARD across genomic elements and their 2k up and 2k down stream. Lines in different colors represents for different elements.

Fig. S3. Profiles of *S. japonica* sporophyte (SP, red track), male (MG, green track), and female (FG, blue track) gametophyte methylomes and transcriptome on

Chromosome 0, an artificial chromosome constructed by scaffolds that were not linked to other genetic linkages.

Fig. S4. Chromosome-specific profiles for *S. japonica* SP, MG, FG methylomes and transcriptomes. For each track, upper panel present the MLgf (average of the three duplicates) along the chromosome; middle panel present the CpG o/e ratio along the chromosome; the down panel present the transcriptome reads counts along chromosome. Chromosomes are split into 10k bins to calculate the MLgf. Lines for SP, FG and MG is colored as red, blue and green respectively. Green boxes represent regions where MG is hyper-methylated whereas blue boxes represent regions where FG is hyper-methylated.

Fig. S5. GC characteristics and methylation levels among chromosomes. A) Characteristics of GC content including CpG Observe/Except value, C+G ratio, CpG ratio, CHG ratio and CHH ratio for each chromosome; B) Methylation level of each chromosome for SP (red plots), MG (green plots) and FG (blue plots). Error bars marked on each plot represent for the standard deviation among three replicates of each life stage.

Fig. S6. Comparison (box plots) of methylation levels of individual cytosines for different contexts (CG, CHG, CHH) of SP, FG and MG. N = 3. P-values were calculated according to Kruskal-Wallis.

Fig. S7. Correlation between CpGo/e (observed/expected) and the methylation level of all life-cycle stages (SP, FG, MG). Genomes were split into 10kb bins according to their CpGo/e and the average methylation level. Each plot represents a 10kb bin of the genome.

Fig. S8. Correlation between C+G percentage (C+G ratio) and the methylation level

of all life-cycle stages. Each plot represents a 10kb bin of the genome.

Fig. S9. Correlation between CpG percentage (CpG ratio) and the methylation level of all life-cycle stages. Each plot represents a 10kb bin of the genome.

Fig. S10. Correlation between CHG percentage (CHG ratio) and the methylation level of life-cycle stages. Each plot represents a 10kb bin of the genome.

Fig. S11. Correlation between CHH percentage (CHH ratio) and the methylation level of all life-cycle stages. Each plot represents a 10kb bin of the genome.

Fig. S12. Correlation between CpG islands ratio and the methylation level of all life-cycle stages. Each plot represents a 10kb bin of the genome.

Fig. S13. Difference of methylation level for types of genomic element across stages. Methylation level for each type of genomic element (e.g. TE, gene, tRNA) was calculated. Error bars denote standard deviation.

Fig. S14. Normalized methylation level (methylation level of genomic elements divided by whole-genome methylation level) for each type of genomic element for SP, FG and MG separately. Different letters indicate significant differences between life-cycle stages (p-value < 0.05).

Fig. S15. A) Methylation for all methylation contexts (e.g. CG, CHG, CHH) of lncRNA genes including their up- and down-stream flanks in SP, FG and MG. B) Methylation for all methylation contexts (e.g. CG, CHG, CHH) of cirRNA genes including their 2K up- and down-stream flanks in SP, FG and MG.

Fig. S16. Most enriched (top 20) KEGG pathways for methylated genes in *S. japonica* female gametophytes. Red color indicates significant enrichment (P-value ≤ 0.05). Richfactor is calculated as the number of methylated genes belong to a KEGG pathway / the number of all genes belong to this pathway. The bigger Richfactor indicate the more obvious enrichment.

Fig. S17. Most enriched (top 20) KEGG pathways for methylated genes in *S. japonica* male gametophytes. Red color indicates significant enrichment (P-value ≤ 0.05). Richfactor is calculated as the number of methylated genes belong to a KEGG pathway / the number of all genes belong to this pathway. The bigger Richfactor indicate the more obvious enrichment.

Fig. S18. Most enriched (top 20) KEGG pathways for methylated genes in *S. japonica* sporophytes. Richfactor is calculated as the number of methylated genes belong to a KEGG pathway / the number of all genes belong to this pathway. The bigger Richfactor indicate the more obvious enrichment.

Fig. S19. Upper panel: Genome-wide gene expression profiling integrating the expression of all genes (values given as FPKM+1 log10) in SP, MG and FG. Lower panel: DNA methylation profiles and expression levels (high, medium, low, none) of each methylation context and life-cycle stage (SP, FG, MG).

Fig. S20. Methylation profiles of the sex-determining region in *S. japonica* including gene expression data. A) Sex-determination regions and their genomic loci in *S. japonica.* Genes inside the oval box are on the autosomal chromosomes, of which, the gray ones having recently been transferred out. The genes in the rectangle box are sex-determining genes, among which the green gene is the male-specific gene and the blue gene is the female-specific gene. The dotted line gene is one that exists but is

missing from the current version of the genome. B) MLgf and gene expression of sex-determination genes. It is noteworthy that it is the absence of the SJ05808 and SJ000352 in FG that lead to the trace-level methylation and gene expression in the stage.

Fig. S21. Base-specific methylation and transcriptional coverage of selected metabolic genes (e.g. cellulose synthase) in *S. japonica*. The different tracks correspond to different life-cycle stages (SP, MG, FG) and biological replicates (1-3). The bottom part shows the gene structure including all exons (thick parts in lines).

Fig. S22. Correlation between the MLgf and gene expression for lncRNA. Dash line indicate the 90% of all data.

Fig. S23. Correlation between the MLgf and gene expression for circRNA. Dash line indicate the 90% of all data.

Fig. S24. The correlation ship between the methylation level (MLgf) of TEs and their expression level. The abbreviation of different TE class: LINE (long interspersed nuclear element), LTR (Long terminal repeat), RC (rolling circle), SINE (short interspersed nuclear element).

Fig. S25. Consistency assessment the result of MeDIP-seq and WGBS by correlating the MLgf of 10k bins from WGBS (Fig. 2 and Fig. S2) with their corresponding NRD from MeDIP-seq (data in Fig1. A and chromosome 0). Only data of MG were used because of the absence of MeDIPseq in SP and FG. Pearson correlation index is employed.

Fig. S26. Validation of the methylation of WGBS data for SJ18945 using BS-PCR. The tracks in 3 colors are Green tracks are the result of Sanger sequence, of which, green tracks stand for the MG, blue tracks stand for FG and red tracks stand for FG. The circle stand for the CpG site and the black plots mean this site are detected as methylated site.

Fig. S27. Validation of the methylation of WGBS data for SJ15797 using BS-PCR. The tracks in 3 colors are Green tracks are the result of Sanger sequence, of which, green tracks stand for the MG, blue tracks stand for FG and red tracks stand for FG. The circle stand for the CpG site and the black plots mean this site are detected as methylated site.

Fig. S28. Validation of the methylation of WGBS data for SJ15874 using BS-PCR. The tracks in 3 colors are Green tracks are the result of Sanger sequence, of which, green tracks stand for the MG, blue tracks stand for FG and red tracks stand for FG. The circle stand for the CpG site and the black plots mean this site are detected as methylated site.

Fig. S29. Validation of the methylation of WGBS data for SJ13722 using BS-PCR. The tracks in 3 colors are Green tracks are the result of Sanger sequence, of which, green tracks stand for the MG, blue tracks stand for FG and red tracks stand for FG. The circle stand for the CpG site and the black plots mean this site are detected as methylated site.

Fig. S30. Validation of the methylation of WGBS data for SJ21435 using BS-PCR. The tracks in 3 colors are Green tracks are the result of Sanger sequence, of which, green tracks stand for the MG, blue tracks stand for FG and red tracks stand for FG. The circle stand for the CpG site and the black plots mean this site are detected as

methylated site.

Fig. S31. Validation of the methylation of WGBS data for SJ22030 using BS-PCR. The tracks in 3 colors are Green tracks are the result of Sanger sequence, of which, green tracks stand for the MG, blue tracks stand for FG and red tracks stand for FG. The circle stand for the CpG site and the black plots mean this site are detected as methylated site.

Fig. S32. Validation of the methylation of WGBS data for SJ15673 using BS-PCR. The tracks in 3 colors are Green tracks are the result of Sanger sequence, of which, green tracks stand for the MG, blue tracks stand for FG and red tracks stand for FG. The circle stand for the CpG site and the black plots mean this site are detected as methylated site.

Fig. S33. Validation of the methylation of WGBS data for SJ19510 using BS-PCR. The tracks in 3 colors are Green tracks are the result of Sanger sequence, of which, green tracks stand for the MG, blue tracks stand for FG and red tracks stand for FG. The circle stand for the CpG site and the black plots mean this site are detected as methylated site.

Fig. S34. Validation of the methylation of WGBS data for SJ22283 using BS-PCR. The tracks in 3 colors are Green tracks are the result of Sanger sequence, of which, green tracks stand for the MG, blue tracks stand for FG and red tracks stand for FG. The circle stand for the CpG site and the black plots mean this site are detected as methylated site.

Fig. S35. Validation of the methylation of WGBS data for SJ20815 using BS-PCR. The tracks in 3 colors are Green tracks are the result of Sanger sequence, of which,

green tracks stand for the MG, blue tracks stand for FG and red tracks stand for FG. The circle stand for the CpG site and the black plots mean this site are detected as methylated site.

Fig. S36. Phylogeny of methytransferases based on Pfam PF00145. Bootstrap values bigger than 50 are marked using red rectangles.

Fig. S37. Domain architecture of candidate methytransferases (DNMT4/5/6) in diatoms (UP5 genes) and homologs in *S. japonica.*

Fig. S38. Methytransferases (DNA methylase domain PF000145) (6x) and their expression under different stress conditions in SP, FG, MG. N (Control), LS (Low salinity), HT (High temperature), HS (High salinity), HL (High light), AC (acidification).

Fig. S39. Genes structure, expression pattern and chromosomal location of genes containing MBT, PWWD, DNMT1, Zinc finger PHD-type and bromodomains in the genome of *S. japonica.*

Fig. S40. Genes Structure, expression pattern and chromosomal location of genes containing ADD, Pre_SET, SET, SNF, helicase and AP2/ERF domains in the genome of *S. japonica*.

Fig. S41. Genes structure (e.g. DME, ROS domains) and phylogeny of demethyltransferases.

Fig. S42. Genes structure of MBD domains and their phylogeny.

Fig. S43. Methylation levels of tRNAs and their correlation with the level of gene methylation. A) Upper panel show the ratio of unmethylated to methylated tRNA genes. Lower panel: MLgf of tRNAs in mitochondria (M), chloroplasts (C) and the nucleus (N) for all life-cycle stages. B) MLgf of tRNA in relation to the gene expression for all three life-cycle stages. Correlation analysis was done using the R package.
